# A four-part working bibliography of neuroethics: part 3 – “second tradition neuroethics” – ethical issues in neuroscience

**DOI:** 10.1186/s13010-016-0037-1

**Published:** 2016-09-19

**Authors:** Amanda Martin, Kira Becker, Martina Darragh, James Giordano

**Affiliations:** 1 Neuroethics Studies Program, Pellegrino Center for Clinical Bioethics, Georgetown University Medical Center, Bldg D, Rm 238, 4000 Reservoir Road, Washington, DC 20057 USA; 2 Department of Neuroscience, Amherst College, Amherst, MA USA; 3 Bioethics Research Library, Kennedy Institute of Ethics, Georgetown University, Washington, DC USA; 4 Departments of Neurology and Biochemistry, Georgetown University Medical Center, Washington, DC USA

**Keywords:** Neuroethics, Neuroscience, Neurotechnology, Ethics, Bibliography

## Abstract

**Background:**

Neuroethics describes several interdisciplinary topics exploring the application and implications of engaging neuroscience in societal contexts. To explore this topic, we present Part 3 of a four-part bibliography of neuroethics’ literature focusing on the “ethics of neuroscience.”

**Methods:**

To complete a systematic survey of the neuroethics literature, 19 databases and 4 individual open-access journals were employed. Searches were conducted using the indexing language of the U.S. National Library of Medicine (NLM). A Python code was used to eliminate duplications in the final bibliography.

**Results:**

This bibliography consists of 1137 papers, 56 books, and 134 book chapters published from 2002 through 2014, covering ethical issues in neuroimaging, neurogenetics, neurobiomarkers, neuro-psychopharmacology, brain stimulation, neural stem cells, neural tissue transplants, pediatric-specific issues, dual-use, and general neuroscience research issues. These works contain explanations of recent research regarding neurotechnology, while exploring ethical issues in future discoveries and use.

## Introduction and background

As a discipline, neuroethics addresses and engages a number of topics that are generated by the intersection of brain science and applications in philosophy, medicine, law, public life, and society (on the local and global scales). In Part 2 of this bibliography [1], we provided a list of works in the scholarly literature that address the neuroscientific basis of moral decision-making and actions, viz.- what Roskies somewhat colloquially refers to as the “neuroscience of ethics” [2]. In Part 3, we herein present a listing of works that discuss the “ethics of neuroscience,” namely those issues, questions and dilemmas generated by current and proposed neuroscientific research and its varied uses. The following bibliographies provide systematic surveys from 2002 to 2014 of the neuroethics literature on translational neuroscience, from “bench to bedside – and beyond” [3, 4] To be sure, the topics are numerous, and list of works is long, and include the following subjects:

- General issues in neuroscience research
- Neuroimaging
- Neurogenetics
- Neurobiomarkers
- Neuro-psychopharmacology
  - anti-depressants; antipsychotic and nootropic agents
  - anti-anxiety agents
  - analgesics
- Brain stimulation/neuromodulation
  - neurofeedback
  - transcranial brain stimulation/neuromodulation
    (i.e.: transcranial direct current stimulation,tDCS; transcranial alternating current stimulation, tACS; and transcranial magnetic stimulation, TMS)
  - deep brain stimulation (DBS)
  - brain-machine interfaces and neural prosthetics
- Neural stem cells and tissue transplants
- Issues concerning pediatric subjects/patients
- Dual-use neuroscientific research

The topics covered involve defining the ethical issues at hand, assessing patient outcomes, and deliberating on quality of life considerations. These neuroethical deliberations also focus on philosophical concepts such as personhood, authenticity, and identity [5, 6]. As an analytical approach and discipline, neuroethics is both pragmatic and pluralistic in its focus and scope [7]. The pragmatism inherent in neuroethics acknowledges “…the contingency of any/all neuroscientific knowledge, and an advocacy for its use *mutatis mutandis*, irrespective of whatever field” to which brain science is applied [8]. In its pluralistic stance, neuroethics is increasingly oriented toward, acknowledging and respectful of, and employing multi-cultural perspectives, ideas, values, needs, contexts and methods, as relevant to the realities of international neuroscientific research and use upon the twenty first century world stage [9–13].

As with Parts 1 and 2, we regard this as a “participatory bibliography” to which readers are encouraged to submit additional cites via the “Comments” section of this paper, or by contacting the bibliographic manager at bioethics@georgetown.edu. Through this process, the bibliography can be and remain as current and accurate as possible, so as to keep pace with ongoing developments in neuroscience, their translational use(s) in practice, and the ethical discourses and debates they foster.

## Methods

Methods for systematically searching relevant literature devoted to neuroethics are identical to those utilized in Parts 1 and 2 of this bibliography [1, 14]. The United States National Library of Medicine’s (NLM) indexing language–MeSH (Medical Subject Headings)–was used to generate the basic search strategy for each topic. MeSH contains ethics-related terms developed for BIOETHICSLINE, a specialty database devoted to bioethical issues produced for NLM by the Kennedy Institute of Ethics from 1975 to 2000.

The following databases were utilized:

1. PubMed (https://pubmed.gov)
2. The NLM Catalog (http://www.ncbi.nlm.nih.gov/nlmcatalog)
3. Academic Search Premier
4. Proquest Research Library
5. JSTOR
6. LexisNexis Academic
7. WorldCat (http://www.worldcat.org)
8. Philosopher’s Index
9. Embase
10. BELIT (http://www.drze.de/belit/)
11. Web of Knowledge/Web of Science (WoS)
12. Digital Public Library of America (DPLA) (https://dp.la/)
13. Directory of Open Access Journals (DOAJ) (https://doaj.org/)
14. Hathi Trust Digital Library (https://www.hathitrust.org/)
15. European Library (http://www.theeuropeanlibrary.org/tel4/)
16. Internet Archive (https://archive.org/ )
17. Globethics.net (http://www.globethics.net/)
18. Neuroethics-Wikiography (https://teamweb.uni-mainz.de/fb05/Neuroethics)
19. Law and Neuroscience Bibliography (http://www.lawneuro.org/bibliography.php)

Open access bioethics journals not contained in the Directory of Open Access Journals (DOAJ) were individually accessed and searched; these included:

1. Journal of Ethics and Social Philosophy from the University of Southern California (http://www.jesp.org/)
2. Journal of Mental Health Ethics from McMaster University (http://www.jemh.ca/)
3. Journal of Practical Ethics (http://www.jpe.ox.ac.uk/) from the Oxford Uehiro Centre for Practical Ethics at the University of Oxford; and
4. Philosophers’ Imprint from the University of Michigan (http://www.philosophersimprint.org/).

## Results

The following bibliographies provide listings of 1137 articles, 56 books, and 134 book chapters published from 2002 through 2014 that focus on the ethical issues involved in translating neuroscientific research to clinical practice.

***Research general issues:***

- Abi-Rached JM: **The implications of the new brain sciences. The 'Decade of the Brain' is over but its effects are now becoming visible as neuropolitics and neuroethics, and in the emergence of neuroeconomies**. *EMBO Rep* 2008, **9**(12):1158-1162. doi: 10.1038/embor.2008.211.
- Alpert S: **Total information awareness—forgotten but not gone: lessons for neuroethics**. *Am J Bioeth* 2007, **7**(5): 24-26.
- Anderson JA, Eijkholt M, Illes J: **Neuroethical issues in clinical neuroscience research**. *Handb Clin Neurol* 2013, **118**: 335-343. doi: 10.1016/B978-0-444-53501-6.00028-7.
- Bergareche AM, da Rocha AC: **Autonomy beyond the brain: what neuroscience offers to a more interactive, relational bioethics**. *AJOB Neurosci* 2011, **2**(3): 54-56. doi: 10.1080/21507740.2011.584948.
- Bird S: **Potential for bias in the context of neuroethics: commentary on “Neuroscience, neuropolitics and neuroethics: the complex case of crime, deception and FMRI”**. *Sci Eng Ethics* 2012, **18**(3): 593-600. doi: 10.1007/s11948-012-9399-y.
- Blakemore C et al.: **Implementing the 3Rs in neuroscience research: a reasoned approach**. *Neuron* 2012, **75**(6):948-950. doi: 10.1016/j.neuron.2012.09.001.
- Brosnan C, Cribb A, Wainwright SP, Williams C: **Neuroscientists’ everyday experiences of ethics: the interplay of regulatory, professional, personal and tangible ethical spheres.***Sociol Health Illn* 2013, **35**(8): 1133-1148. doi: 10.1111/1467-9566.12026.
- Caulfield T, Ogbogu U: **Biomedical research and the commercialization agenda: a review of main considerations for neuroscience**. *Account Res* 2008, **15**(4): 303-320. doi: 10.1080/08989620802388788.
- Chatterjee A: **The ethics of neuroenhancement**. *Handb Clin Neurol* 2013, **118**: 323-34. doi: 10.1016/B978-0-444-53501-6.00027-5.
- Cheshire WP: **Neuroscience, nuance, and neuroethics**. *Ethics Med* 2006, **22**(2): 71-3.
- Cheung EH: **A new ethics of psychiatry: neuroethics, neuroscience, and technology**. *J Psychiatr Pract* 2009, **15**(5): 391-401. doi: 10.1097/01.pra.0000361279.11210.14.
- Choudhury S, Nagel SK, Slaby J: **Critical neuroscience: linking neuroscience and society through critical practice**. *Biosocieties* 2009, **4**:61-77. doi:10.1017/S1745855209006437.
- Cohen PD et al.: **Ethical issues in clinical neuroscience research: a patient’s perspective.***Neurotherapeutics* 2007, **4**(3): 537-544. doi: 10.1016/j.nurt.2007.04.008.
- Crozier S: **[Neuroethics: ethical issues in neurosciences]**. *Rev Prat* 2013, **63**(5): 666-669.
- Decker M, Fleischer T: **Contacting the brain—aspects of a technology assessment of neural implants**. *Biotechnol J* 2008, **3**(12): 1502-1510. doi: 10.1002/biot.200800225.
- Di Luca M et al.: **Consensus document on European brain research**. *Eur J Neurosci* 2011, **33**(5):768-818. doi: 10.1111/j.1460-9568.2010.07596.x.
- Eaton ML, Illes J: **Commercializing cognitive neurotechnology--the ethical terrain**. *Nat Biotechnol* 2007, **25**(4): 393-397. doi:10.1038/nbt0407-393.
- Farah MJ: **Neuroethics: the practical and the philosophical**. *Trends Cogn Sci* 2005, **9**(1): 34-40. doi: 10.1016/j.tics.2004.12.00.
- Farah MJ: **Social, legal, and ethical implications of cognitive neuroscience: “neuroethics” for short**. *J Cogn Neurosci* 2007, **19**(3): 363-4. doi: 10.1162/jocn.2007.19.3.363.
- Fellows LK, Stark M, Berg A, Chatterjee A: **Patient registries in cognitive neuroscience research: advantages, challenges, and practical advice**. *J Cogn Neurosci* 2008, **20**(6): 1107-1113. doi: 10.1162/jocn.2008.20065.
- Fins JJ: **From psychosurgery to neuromodulation and palliation: history’s lessons for the ethical conduct and regulation of neuropsychiatricresearch**. *Neurosurg Clin N Am* 2003, **14**(2): 303-19. doi: 10.1016/S1042-3680(02)00118-3.
- Fischer MMJ: **The BAC [bioethics advisory committee] consultation on neuroscience and ethics: an anthropologist’s perspective**. *Innovation* 2013, **11**(2): 3-5.
- Ford PJ: **Special section on clinical neuroethics consultation: introduction**. *HEC Forum* 2008, **20**(3): 311-4. doi: 10.1007/s10730-008-9081-6.
- Fry CL: **A descriptive social neuroethics is needed to reveal lived identities**. *Am J Bioeth* 2009, **9**(9): 16-7. doi: 10.1080/15265160903098580
- Fuchs T: **Ethical issues in neuroscience**. *Curr Opin Psychiatry* 2006, **19**(6): 600-607. doi: 10.1097/01.yco.0000245752.75879.26.
- Fukushi T, Sakura O, Koizumi H: **Ethical considerations of neuroscience research: the perspectives on neuroethics in Japan**. *Neurosci Res* 2007, **57**(1): 10-16. doi: 10.1016/j.neures.2006.09.004.
- Fukushi T, Sakura O: **Exploring the origin of neuroethics: from the viewpoints of expression and concepts**. *Am J Bioeth* 2008, **8**(1): 56-57. doi: 10.1080/15265160701839672.
- Fukushi T, Sakura O: **[Introduction of neuroethics: out of clinic, beyond academia in human brain research]**. *Rinsho Shinkeigaku* 2008, **48**(11): 952-954. doi: 10.5692/clinicalneurol.48.952.
- Galpern WR et al.: **Sham neurosurgical procedures in clinical trials for neurodegenerative diseases: scientific and ethical considerations**. *Lancet Neurol* 2012, **11**(7): 643-650. doi: 10.1016/S1474-4422(12)70064-9.
- Glannon W: **Neuroethics**. *Bioethics* 2006, **20**(1): 37-52. doi: 10.1111/j.1467-8519.2006.00474.x.
- Gray JR, Thompson PM: **Neurobiology of intelligence: science and ethics**. *Nat Rev Neurosci* 2004, **5**(6): 471-482. doi: 10.1038/nrn1405.
- Gutiérrez G: **Neurobiología y contenido material universal de la ética: reflexiones a partir del modelo neurobiológico de Antonio Damasio**. *Utop Prax Latinoam* 2006, **11**(33): 9-38.
- Hauser SL: **What ethics integration looks like in neuroscience research**. *Ann Neurol* 2014, **75**(5): 623-624. doi: 10.1002/ana.24177.
- Henry S, Plemmons D: **Neuroscience, neuropolitics and neuroethics: the complex case of crime, deception and FMRI**. *Sci Eng Ethics* 2012, **18**(3): 573-591. doi: 10.1007/s11948-012-9393-4.
- Illes J: **Empirical neuroethics: can brain imaging visualize human thought? why is neuroethics interested in such a possibility?***EMBO Rep* 2007, **8**: S57-S60. doi: 10.1038/sj.embor.7401007.
- Illes J: **Empowering brain science with neuroethics**. *Lancet* 2010, **376**(9749): 1294-1295. doi: 10.1016/S0140-6736(10)61904-6.
- Illes J et al.: **International perspectives on engaging the public in neuroethics**. *Nat Rev Neurosci* 2005, **6**(12): 977-982. doi:10.1038/nrn1808.
- Illes J, Raffin TA: **Neuroethics: an emerging new discipline in the study of brain and cognition**. *Brain Cogn* 2002, **50**(3): 341-344. doi: 10.1016/s0278-2626(02)00522-5.
- Illes J, Bird SJ: **Neuroethics: a modern context for ethics in neuroscience**. *Trends Neurosci* 2006, **29**(9): 511-517. doi: 10.1016/j.tins.2006.07.002.
- Illes J et al.: **Neurotalk: improving the communication of neuroscience research**. *Nat Rev Neurosci* 2010, **11**(1): 61-69. doi: 10.1038/nrn2773.
- Illes J et al.: **Reducing barriers to ethics in neuroscience**. *Front Hum Neurosci* 2010, **4**: 167. doi: 10.3389/fnhum.2010.00167.
- Jonsen AR: **What it means to “map” the field of neuroethics**. *Cerebrum* 2002, **4**(3): 71-72.
- Jox RJ, Schöne-Seifert B, Brukamp K: **[Current controversies in neuroethics]**. *Nervenarzt* 2013, **84**(10):1163-1164. doi: 10.1007/s00115-013-3731-x.
- Justo L, Erazun F: **Neuroethics needs an international human rights deliberative frame.***AJOB Neurosci* 2010, **1**(4): 17-18. doi: 10.1080/21507740.2010.515559.
- Kirschenbaum SR: **Patenting basic research: myths and realities**. *Nat Neurosci* 2002, **5**: Suppl 1025-1027. doi: 10.1038/nn932.
- Klein E: **Is there a need for clinical neuroskepticism?***Neuroethics* 2011, **4**(3): 251-259. doi: 10.1007/s12152-010-9089-x
- Kretzschmar H: **Brain banking: opportunities, challenges and meaning for the future**. *Nat Rev Neurosci* 2009, **10**(1): 70-78. doi: 10.1038/nrn2535.
- Labuzetta JN, Burnstein R, Pickard J: **Ethical issues in consenting vulnerable patients for neuroscience research**. *J Psychopharmacol* 2011, **25**(2): 205-210. doi: 10.1177/0269881109349838.
- Lanzilao E, Shook JR, Benedikter R, Giordano J: **Advancing neuroscience on the 21st-century world stage: the need for and a proposed structure of an internationally relevant neuroethics**. *Ethics Biol Eng Med* 2013, **4**(3), 211-229. doi: 10.1615/EthicsBiologyEngMed.2014010710.
- Leonardi M et al.: **Pain, suffering and some ethical issues in neuroscience research**. *J Headache Pain* 2004, **5**(2): 162-164. doi: 10.1007/s10194-004-0088-3.
- Leshner AI: **Ethical issues in taking neuroscience research from bench to bedside.***Cerebrum* 2004, **6**(4): 66-72.
- Lieberman MD: **Social cognitive neuroscience: a review of core processes**. *Annu Rev Psychol* 2007, **58**: 259-289. doi: 10.1146/annurev.psych.58.110405.085654.
- Lombera S, Illes J: **The international dimensions of neuroethics**. *Dev World Bioeth* 2009, **9**(2):57-64. doi: 10.1111/j.1471-8847.2008.00235.x.
- Mandel RJ, Burger C: **Clinical trials in neurological disorders using AAV vectors: promises and challenges**. *Curr Opin Mol Ther* 2004, **6**(5):482-490.
- Mauron A: **[Neuroethics]**. *Rev Med Suisse* 2006, **2**(74): 1816.
- Miller FG, Kaptchuk TJ: **Deception of subjects in neuroscience: an ethical analysis**. *J Neurosci* 2008, **28**(19): 4841-4843. doi: 10.1523/JNEUROSCI.1493-08.2008.
- Morein-Zamir S, Sahakian BJ: **Neuroethics and public engagement training needed for neuroscientists**. *Trends Cogn Sci* 2010, **14**(2): 49-51. doi: 10.1016/j.tics.2009.10.007.
- Northoff G: **[Methodological deficits in neuroethics: do we need theoretical neuroethics?]**. *Nervenarzt* 2013, **84**(10):1196-1202. doi: 10.1007/s00115-013-3732-9.
- Nutt DJ, King LA, Nichols DE: **Effects of Schedule 1 drug laws on neuroscience research and treatment innovation**. *Nat Rev Neurosci* 2013, **14**(8): 577-585. doi: 10.1038/nrn3530.
- Olesen J: **Consensus document on European brain research**. *J Neurol Neurosurg Psychiatry* 2006, **77** (Supp 1):i1-i49.
- Parens E, Johnston J: **Does is make any sense to speak of neuroethics: three problems with keying ethics to hot new science and technology**. *EMBO Rep* 2007, **8**: S61-S64. doi:10.1038/sj.embor.7400992.
- Parker LS, Kienholz ML: **Disclosure issues in neuroscience research**. *Account Res* 2008, **15**(4): 226-241. doi: 10.1080/08989620802388697.
- Paylor B, Longstaff H, Rossi F, Illes J: **Collision or convergence: beliefs and politics in neuroscience discovery, ethics, and intervention**. *Trends Neurosci* 2014, **37**(8): 409-412. doi: 10.1016/j.tins.2014.06.001.
- Perrachione TK, Perrachione JR: **Brains and brands: developing mutually informative research in neuroscience and marketing**. *J Consumer Behav* 2008, **7**: 303-318. doi: 10.1002/cb.253.
- Pfaff DW, Kavaliers M, Choleris E: **Response to Peer Commentaries on Mechanisms Underlying an Ability to Behave Ethically—Neuroscience Addresses Ethical Behaviors: Transitioning From Philosophical Dialogues to Testable Scientific Theories of Brain and Behavior**. *Am J Bioeth* 2008, **8**(5): W1-W3. doi: 10.1080/15265160802180117.
- Pickersgill M: **Ordering disorder: knowledge production and uncertainty in neuroscience research**. *Sci Cult (Lond)* 2011, **20**(1): 71-87. doi: 10.1080/09505431.2010.508086.
- Pierce R: **What a tangled web we weave: ethical and legal implications of deception in recruitment**. *Account Res* 2008, **15**(4): 262-282. doi: 10.1080/08989620802388713.
- Racine E, Waldman S, Rosenberg J, Illes J: **Contemporary neuroscience in the media**. *Soc Sci Med* 2010, **71**(4): 725-733. doi: 10.1016/j.socscimed.2010.05.017.
- Racine E: **Identifying challenges and conditions for the use of neuroscience in bioethics**. *Am J Bioeth* 2007, **7**(1): 74-76. doi: 10.1080/15265160601064363.
- Racine E, Illes J: **Responsabilités neuroéthiques/neuroethical responsibilities**. *Can J Neurol Sci* 2006, **33**(3): 260-268, 269-277. doi: 10.1017/S0317167100005126.
- Ramos-Zúñiga R: **[Neuroethics as a new epistemological perspective in neuroscience]**. *Rev Neurol* 2014, **58**(4): 145-146.
- Robillard JM et al.: **Untapped ethical resources for neurodegeneration research**. *BMC Med Ethics* 2011, **12**: 9. doi: 10.1186/1472-6939-12-9.
- Rose N: **The human brain project: social and ethical challenges**. *Neuron* 2014, **82**(6):1212-1215. doi:10.1016/j.neuron.2014.06.001.
- Rose N: **The human sciences in a biological age**. *Theory Cult Soc* 2013, **30**(1): 3-34. doi: 10.1177/0263276412456569.
- Roskies A: **Neuroethics for the new millenium**. *Neuron* 2002, **35**(1): 21-23. doi: 10.1016/S0896-6273(02)00763-8.
- Schreiber D: **On social attribution: implications of recent cognitive neuroscience research for race, law, and politics**. *Sci Eng Ethics* 2012, **18**(3): 557-566. doi: 10.1007/s11948-012-9381-8.
- Shook JR, Giordano J: **A principled and cosmopolitan neuroethics: considerations for international relevance**. *Philos Ethics Humanit Med* 2014, **9**:1. doi: 10.1186/1747-5341-9-1.
- Stevenson S et al.: **Neuroethics, confidentiality, and a cultural imperative in early onset Alzheimer’s disease: a case study with a First Nation population**. *Philos Ethics Humanit Med* 2013, **8**: 15. doi: 10.1186/1747-5341-8-15.
- Synofzik M: **Interventionen zwischen gehirn und geist: eine ethische analyse der neuen moglichkeiten der neurowissenschaften [Intervening between brain and mind: an ethical analysis of the new possibilities of the neurosciences]**. *Fortschr Neurol Psychiatr* 2005, **73**(10):596-604. doi:10.1055/s-2004-830292.
- Swift TL: **Sham surgery trial controls: perspectives of patients and their relatives**. *J Empir Res Hum Res Ethics* 2012, **7**(3): 15-28. doi: 10.1525/jer.2012.7.3.15.
- Weisberg DS et al.: **The seductive allure of neuroscience explanations**. *J Cogn Neurosci* 2008, **20**(3): 470-477. doi: 10.1162/jocn.2008.20040.
- Winslade W: **Severe brain injury: recognizing the limits of treatment and exploring the frontiers of research**. *Camb Q Healthc Ethics* 2007, **16**(2): 161-168. doi: 10.1017/S0963180107070181.
- Wolpe PR: **Ethics and social policy in research on the neuroscience of human sexuality**. *Nature Neuroscience* 2004, **7**(10): 1031-1033. 10.1038/nn1324.
- Zimmerman E, Racine E: **Ethical issues in the translation of social neuroscience: a policy analysis of current guidelines for public dialogue in human research**. *Account Res* 2012, **19**(1): 27-46. doi: 10.1080/08989621.2012.650949.

Books:

- Ackerman S: *Hard Science, Hard Choices: Facts, Ethics, and Policies Guiding Brain Science Today*. New York: Dana Press; 2006.
- Centre for Educational Research and Innovation, Organisation for Economic Co-operation and Development. *Understanding the Brain: The Birth of a Learning Science*. Paris: OECD; 2007.
- Harrington M: *The Design of Experiments in Neuroscience*. Thousand Oaks, CA: Sage; 2011.
- National Research Council (U.S.). Committee on Military and Intelligence Methodology for Emergent Neurophysiological and Cognitive/Neural Science Research in the Next Two Decades. *Emerging Cognitive Neuroscience and Related Technologies*. Washington, DC: National Academies Press; 2008.
- Sousa DA: *Brainwork: The Neuroscience Behind How We Lead Others*. Bloomington, IN: Triple Nickel Press; 2012.
- Taylor KE: *The Brain Supremacy: Notes from the Frontiers of Neuroscience*. Oxford: Oxford University Press; 2012.

Book chapters:

- Alves WM: **Ethical considerations in neuroemergency clinical trials**. In *Handbook of Neuroemergency Clinical Trials*. Edited by Wayne M. Alves. Amsterdam: Elsevier Academic Press; 2006: 257-273.
- Anderson JA, Eijkholt M, Illes J: **Neuroethical issues in clinical neuroscience research**. In *Ethical and Legal Issues in Neurology*. Edited by James L. Bernat, H. Richard Beresford. Edinburgh: Elsevier; 2013: 335-344.
- Birge S: **Brainhood, selfhood, or “meat with a point of view”: the value of fiction for neuroscientific research and neurological medicine**. In *Neuroscientific Turn: Transdisciplinarity in the Age of the Brain*. Edited by Melissa M. Littlefield, Jenell M. Johnson. Ann Arbor, MI: University of Michigan Press; 2012: 89-104.
- Borck C: **Toys are us: models and metaphors in brain research**. In *Critical Neuroscience: A Handbook of the Social and Cultural Contexts of Neuroscience*. Edited by Suparna Choudhury, Jan Slaby. Chichester, West Sussex: Wiley-Blackwell; 2012: 113-134.
- Detre J, Bockow TB: **Incidental findings in magnetic resonance imaging research**. In *Neuroethics in Practice*. Edited by Anjan Chatterjee, Martha J. Farah. New York: Oxford University Press; 2012: 120-127.
- Ford PJ, Deshpande A: **The ethics of surgically invasive neuroscience research.** In *Ethical and Legal Issues in Neurology*. Edited by James L. Bernat, H. Richard Beresford. Edinburgh: Elsevier; 2013: 315-322.
- Giordano J: **Integrative convergence in neuroscience: trajectories, problems and the need for a progressive neurobioethics**. In *Technological Innovations in Sensing and Detection of Chemical, Biological, Radiological, Nuclear Threats and Ecological Terrorism*. Edited by A. Vaseashta, Eric Braman, Philip Susmann. Dordrecht: Springer; 2012: 115-130.
- Giordano J, Waters P: **What might renewed focus on brain research mean for the prevention and care of brain injury and its effects?** In their *Brain Injury: Spectrum Effects and Implications*. Edited by James Giordano and Patrick Waters. Arlington, VA: Potomac Institute Press; 2013: xiii-xxiv.
- Hochberg L, Cochrane T: **Implanted neural interfaces: ethics in treatment and research**. In *Neuroethics in Practice*. Edited by Anjan Chatterjee, Martha J. Farah. New York: Oxford University Press; 2012: 234-250.
- Hurst S: **Clinical research on conditions affecting cognitive capacity**. In *The Oxford Handbook of Neuroethics*. Edited by Judy Illes and Barbara J. Sahakian. Oxford: Oxford University Press; 2011: 513-528.
- Kim SYH: **Competence for informed consent for treatment and research**. In *Neuroethics in Practice*. Edited by Anjan Chatterjee, Martha J. Farah. New York: Oxford University Press; 2012: 83-95.
- Linden D: **The ethics and politics of brain control**. In his *Brain Control: Developments in Therapy and Implications for Society*. London: Palgrave Macmillan; 2014: 131-171.
- Miller FG, Fins JJ: **Protecting human subjects in brain research: a pragmatic perspective**. In *Neuroethics: Defining the Issues in Theory, Practice, and Policy*. Edited by Judy Illes. Oxford: Oxford University Press; 2006: 123-140.
- Murray S, Yanagi MA: **Transitioning brain research: from bench to battlefield**. In *Neurotechnology in National Security and Defense: Practical Considerations, Neuroethical Concerns*. Edited by James Giordano. Boca Raton: CRC Press; 2014 [2015]:11-22.
- Racine E: **Public understanding of neuroscience innovation and emerging interpretations of neuroscience research**. In his *Pragmatic Neuroethics: Improving Treatment and Understanding of the Mind-Brain*. Cambridge, Mass.: MIT Press; 2010: 97-120.
- Wolf SM: **Incidental findings in neuroscience research: a fundamental challenge to the structure of bioethics and health law**. In *The Oxford Handbook of Neuroethics*. Edited by Judy Illes and Barbara J. Sahakian. Oxford: Oxford University Press; 2011: 623-634.

***Neuroimaging:***

- Aggarwal NK: **Neuroimaging, culture, and forensic psychiatry**. *J Am Acad Psychiatry Law* 2009, **37**(2): 239-244.
- Aktunç E.: **Tackling Duhemian problems: an alternative to skepticism of neuroimaging in philosophy of cognitive science**. *Rev Philos Psychol* 2014, **5**(4): 449-464. doi: 10.1007/s13164-014-0186-3.
- Al-Delaimy WK: **Ethical concepts and future challenges of neuroimaging: an Islamic perspective**. *Sci Eng Ethics* 2012, **18**(3): 509-518. doi: 10.1007/s11948-012-9386-3.
- Alpert S: **The SPECTer of commercial neuroimaging**. *AJOB Neurosci* 2012, **3**(4): 56-58. doi: 10.1080/21507740.2012.721450.
- Anderson JA, Illes J: **Neuroimaging and mental health: drowning in a sea of acrimony**. *AJOB Neurosci* 2012, **3**(4): 42-43. doi: 10.1080/21507740.2012.721454.
- Anderson J, Mizgalewicz A, Illes J: **Reviews of functional MRI: the ethical dimensions of methodological critique**. *PLoS ONE* 2012, **7**(8): e42836. doi: 10.1371/journal.pone.0042836.
- Anderson JA, Mizgalewicz A, Illes J: **Triangulating perspectives on functional neuroimaging for disorders of mental health**. *BMC Psychiatry* 2013, **13**: 208. doi: 10.1186/1471-244X-13-208.
- Annas G: **Foreword: imagining a new era of neuroimaging, neuroethics, and neurolaw**. *Am J Law Med* 2007, **33**(2-3): 163-170. doi:10.1177/009885880703300201.
- Bluhm R: **New research, old problems: methodological and ethical issues in fMRI research examining sex/gender differences in emotion processing**. *Neuroethics* 2013, **6**(2): 319-330. doi: 10.1007/s12152-011-9143-3.
- Borgelt EL, Buchman DZ, Illes J: **Neuroimaging in mental health care: voices in translation**. *Front Hum Neurosci* 2012, **6**: 293. doi: 10.3389/fnhum.2012.00293.
- Borgelt E, Buchman DZ, Illes J: “**This Is why you’ve been suffering”: reflections of providers on neuroimaging in mental health care**. *J Bioeth Inq* 2011, **8**(1): 15-25. doi: 10.1007/s11673-010-9271-1.
- Boyce AC: **Neuroimaging in psychiatry: evaluating the ethical consequences for patient care**. *Bioethics* 2009, **23**(6): 349-359. doi: 10.1111/j.1467-8519.2009.01724.x. 
- Brakewood B, Poldrack RA: **The ethics of secondary data analysis: considering the application of Belmont principles to the sharing of neuroimaging data**. *Neuroimage* 2013, **82**: 671-676. doi:10.1016/j.neuroimage.2013.02.040.
- Brindley T, Giordano J: **Neuroimaging: correlation, validity, value, and admissibility: Daubert—and reliability—revisited**. *AJOB Neurosci* 2014, **5**(2): 48-50. doi: 10.1080/21507740.2014.884186.
- Canli T, Amin Z: **Neuroimaging of emotion and personality: scientific evidence and ethical considerations**. *Brain Cogn* 2002, **50**(3): 414-431. doi:10.1016/S0278-2626(02)00517-1.
- Cheshire WP: **Can grey voxels resolve neuroethical dilemmas?***Ethics Med* 2007, **23**(3): 135-140.
- Coch D: **Neuroimaging research with children: ethical issues and case scenarios**. *J Moral Educ* 2007, **36**(1): 1-18. doi: 10.1080/03057240601185430.
- d’Abrera JC et al.: **A neuroimaging proof of principle study of Down’s syndrome and dementia: ethical and methodological challenges in intrusive research**. *J Intellect Disabil Res* 2013, **57**(2): 105-118. doi: 10.1111/j.1365-2788.2011.01495.x.
- de Champlain J, Patenaude J: **Review of a mock research protocol in functional neuroimaging by Canadian research ethics boards**. *J Med Ethics* 2006, **32**(9): 530-534. doi: 10.1136/jme.2005.012807.
- Deslauriers C et al.: **Perspectives of Canadian researchers on ethics review of neuroimaging research**. *J Empir Res Hum Res Ethics* 2010, **5**(1): 49-66. doi: 10.1525/jer.2010.5.1.49.
- Di Pietro NC, Illes J: **Disclosing incidental findings in brain research: the rights of minors in decision-making**. *J Magn Reason Imaging* 2013, **38**(5): 1009-1013. doi: 10.1002/jmri.24230.
- Downie J, Hadskis M: **Finding the right compass for issue-mapping in neuroimaging**. *Am J Bioeth* 2005, **5**(2): 27-29. doi: 10.1080/15265160590960285.
- Downie J et al.: **Paediatric MRI research ethics: the priority issues**. *J Bioeth Inq* 2007, **4**(2): 85-91. doi: 10.1007/s11673-007-9046-5.
- Downie J, Marshall J: **Pediatric neuroimaging ethics***. Camb Q Healthc Ethics* 2007, **16**(2): 147-160. doi: 10.1017/S096318010707017X 
- Eaton ML, Illes J: **Commercializing cognitive neurotechnology – the ethical terrain**. *Nat Biotechnol* 2007, **25**(4): 393-397. doi: 10.1038/nbt0407-393.
- Evers K, Sigman M: **Possibilities and limits of mind-reading: a neurophilosophical perspective**. *Conscious Cogn* 2013, **22**(3):887-897. doi: 10.1016/j.concog.2013.05.011.
- Farah MJ: **Brain images, babies, and bathwater: critiquing critiques of functional neuroimaging**. *Hastings Cent Rep* 2014, **44**(S2): S19-S30. doi: 10.1002/hast.295.
- Farah MJ et al.: **Brain imaging and brain privacy: a realistic concern?***J Cogn Neurosci* 2009, **21**(1): 119-127. doi: 10.1162/jocn.2009.21010.
- Farah MJ, Wolpe PR: **Monitoring and manipulating brain function: new neuroscience technologies and their ethical implications**. *Hastings Cent Rep* 2004, **34**(3): 35-45. doi: 10.2307/3528418.
- Farah MJ, Gillihan SJ: **The puzzle of neuroimaging and psychiatric diagnosis: technology and nosology in an evolving discipline**. *AJOB Neurosci* 2012, **3**(4): 31-41. doi: 10.1080/21507740.2012.713072.
- Farah MJ, Hook CJ: **The seductive allure of “seductive allure”.***Perspect Psychol Sci* 2013, **8**(1): 88-90. doi: 10.1177/1745691612469035.
- Fine C: **Is there neurosexism in functional neuroimaging investigations of sex differences?***Neuroethics* 2013, **6**(2): 369-409. doi: 10.1007/s12152-012-9169-1.
- Fins JJ: **The ethics of measuring and modulating consciousness: the imperative of minding time**. *Prog Brain Res* 2009, **177**: 371-382. doi: 10.1016/S0079-6123(09)17726-9.
- Fins JJ, Illes J: **Lights, camera, inaction? neuroimaging and disorders of consciousness**. *Am J Bioeth* 2008, **8**(9): W1-W3. doi: 10.1080/15265160802479568.
- Fins JJ: **Neuroethics and neuroimaging: moving towards transparency**. *Am J Bioeth* 2008, **8**(9): 46-52. doi: 10.1080/15265160802334490.
- Fins JJ: **Neuroethics, neuroimaging, and disorders of consciousness: promise or peril?***Trans Am Clin Climatol Assoc* 2011, **122**: 336-346.
- Fins JJ et al.: **Neuroimaging and disorders of consciousness: envisioning an ethical research agenda**. *Am J Bioeth* 2008, **8**(9): 3-12. doi: 10.1080/15265160802318113.
- Fins JJ, Shapiro ZE: **Neuroimaging and neuroethics: clinical and policy considerations**. *Curr Opin Neurol* 2007, **20**(6): 650-654. doi: 10.1097/WCO.0b013e3282f11f6d.
- Fins JJ: **Rethinking disorders of consciousness: new research and its implications**. *Hastings Cent Rep* 2005, **35**(2): 22-24. doi: 10.1353/hcr.2005.0020.
- Fisher CE: **Neuroimaging and validity in psychiatric diagnosis**. *AJOB Neurosci* 2012, **3**(4): 50-51. doi: 10.1080/21507740.2012.721471.
- Fisher DB, Truog RD: **Conscientious of the conscious: interactive capacity as a threshold marker for consciousness**. *AJOB Neurosci* 2013, **4**(4): 26-33. doi: 10.1080/21507740.2013.819391.
- Fitsch H: **(A)e(s)th(et)ics of brain imaging: visibilities and sayabilities in functional magnetic resonance imaging**. *Neuroethics* 2012, **5**(3): 275-283. doi: 10.1007/s12152-011-9139-z.
- Friedrich O: **Knowledge of partial awareness in disorders of consciousness: implications for ethical evaluations?***Neuroethics* 2013, **6**(1): 13-23. doi: 10.1007/s12152-011-9145-1.
- Garnett A, Lee G, Illes J: **Publication trends in neuroimaging of minimally conscious states**. *PeerJ* 2013 **1**:e155. doi: 10.7717/peerj.155.
- Gauthier S, Leuzy A, Racine E, Rosa-Neto P: **Diagnosis and management of Alzheimer’s disease: past, present and future ethical issues.***Prog Neurobiol* 2013, **110**: 102-113. doi: 10.1016/j.pneurobio.2013.01.003.
- Giordano J: **Neuroimaging in psychiatry: approaching the puzzle as a piece of the bigger picture(s)**. *AJOB Neurosci* 2012, **3**(4): 54-56. doi: 10.1080/21507740.2012.721469.
- Giordano J, DuRousseau D: **Toward right and good use of brain-machine interfacing neurotechnologies: ethical issues and implications for guidelines and policy**. *Cog Tech* 2010, **15**(2): 5-10.
- Gjoneska B: **Neuroimaging and neuroethics: imaging the ethics of neuroscience**. *Prilozi* 2012, **33**(1): 419-424.
- Gruber D, Dickerson JA: **Persuasive images in popular science: testing judgments of scientific reasoning and credibility**. *Public Underst Sci* 2012, **21**(8): 938-948. doi: 10.1177/0963662512454072.
- Hadskis M et al.: **The therapeutic misconception: a threat to valid parental consent for pediatric neuroimaging research**. *Account Res* 2008, **15**(3): 133-151. doi: 10.1080/08989620801946917.
- Heinemann T et al.: **Incidental findings in neuroimaging: ethical problems and solutions**. *Dtsch Arztebl* 2007, **104**(27): A-1982-1987.
- Heinrichs B: **A new challenge for research ethics: incidental findings in neuroimaging**. *J Bioeth Inq* 2011, **8**(1): 59-65. doi: 10.1007/s11673-010-9268-9.
- Hinton VJ: **Ethics of neuroimaging in pediatric development**. *Brain Cogn* 2002, **50**(3): 455-468. doi:10.1016/S0278-2626(02)00521-3.
- Hook CJ, Farah MJ: **Look again: effects of brain images and mind-brain dualism on lay evaluations of research**. *J Cogn Neurosci* 2013, **25**(9): 1397-1405. doi: 10.1162/jocn_a_00407.
- Huber CG, Huber J: **Epistemological considerations on neuroimaging—a crucial prerequisite for neuroethics**. *Bioethics* 2009, **23**(6): 340-348. doi: 10.1111/j.1467-8519.2009.01728.x.
- Huber CG, Kummer C, Huber J: **Imaging and imagining: current positions on the epistemic priority of theoretical concepts and data psychiatric neuroimaging**. *Curr Opin Psychiatry* 2008, **21**(6): 625-629. doi: 10.1097/YCO.0b013e328314b7a1.
- Ikeda K et al.: **Neuroscientific information bias in metacomprehension: the effect of brain images on metacomprehension judgment of neuroscience research***Psychon Bull Rev* 2013, **20**(6): 1357-1363. doi: 10.3758/s13423-013-0457-5.
- Illes J et al.: **Discovery and disclosure of incidental findings in neuroimaging research.***J Magn Reson Imaging* 2004, **20**(5): 743-747. doi: 10.1002/jmri.20180.
- Illes J et al.: **Ethical consideration of incidental findings on adult brain MRI in research**. *Neurology* 2004, **62**(6): 888-890. doi: 10.1212/01.WNL.0000118531.90418.89.
- Illes J, Kirschen MP, Gabrieli JD: **From neuroimaging to neuroethics**. *Nat Neurosci* 2003, **6**(3): 205. doi: 10.1038/nn0303.205.
- Illes J, Racine E: **Imaging or imagining? a neuroethics challenge informed by genetics**. *Am J Bioeth* 2005, **5**(2): 5-18. doi: 10.1080/15265160590923358.
- Illes J, Lombera S, Rosenberg J, Arnow B: **In the mind’s eye: provider and patient attitudes on functional brain imaging**. *J Psychiatr Res* 2008, **43**(2): 107-114. doi: 10.1016/j.jpsychires.2008.02.008.
- Illes J: **Neuroethics in a new era of neuroimaging**. *AJNR Am J Neuroradiol* 2003, **24**(9): 1739-1741.
- Illes J, Kirschen M: **New prospects and ethical challenges for neuroimaging within and outside the health care system**. *AJNR Am J Neuroradiol* 2003, **24**(10): 1932-1934.
- Illes J, Rosen A, Greicius M, Racine E: **Prospects for prediction: ethics analysis of neuroimaging in Alzheimer’s disease**. *Ann N Y Acad Sci* 2007, **1097**: 278-295. doi: 10.1196/annals.1379.030.
- Intriago AR: **Neuroimaging and causal responsibility**. *AJOB Neurosci* 2012, **3**(4): 60-62. doi: 10.1080/21507740.2012.721463.
- Jox RJ: **Interface cannot replace interlocution: why the reductionist concept of neuroimaging-based capacity determination fails**. *AJOB Neurosci* 2013, **4**(4): 15-17. doi: 10.1080/21507740.2013.827279.
- Kabraji S, Naylor E, Wood D: **Reading minds? ethical implications of recent advances in neuroimaging**. *Penn Bioeth J* 2008, **4**(2): 9-11.
- Kahane G: **Brain imaging and the inner life**. *Lancet* 2008, **371**(9624): 1572-1573. doi: 10.1016/S0140-6736(08)60679-0.
- Kaposy C: **Ethical muscle and scientific interests: a role for philosophy in scientific research**. *Q Rev Biol* 2008, **83**(1): 77-86. doi: 10.1086/529565.
- Keehner M, Mayberry L, Fischer MH: **Different clues from different views: the role of image format in public perceptions of neuroimaging results**. *Psychon Bull Rev* 2011, **18**(2): 422-428. doi: 10.3758/s13423-010-0048-7.
- Kehagia AA et al.: **More education, less administration: reflections of neuroimagers’ attitudes to ethics through the qualitative looking glass**. *Sci Eng Ethics* 2012, **18**(4): 775-788. doi: 10.1007/s11948-011-9282-2.
- Kirschen MP, Jaworska A, Illes J: **Subjects’ expectations in neuroimaging research**. *J Magn Reson Imaging* 2006, **23**(2): 205-209. doi: 10.1002/jmri.20499.
- Klein DA, Russell M: **Include objective quality-of-life assessments when making treatment decisions with patients possessing covert awareness**. *AJOB Neurosci* 2013, **4**(4): 19-21. doi: 10.1080/21507740.2013.827277.
- Kulynych J: **Legal and ethical issues in neuroimaging research: human subjects protection, medical privacy, and the public communication of research results**. *Brain Cogn* 2002, **50**(3): 345-357. doi: 10.1016/s0278-2626(02)00518-3.
- Kumra S et al**.: Ethical and practical considerations in the management of incidental findings in pediatric MRI studies**. *J Am Acad Child Adolesc Psychiatry* 2006, **45**(8): 1000-1006. doi: 10.1097/01.chi.0000222786.49477.a8.
- Lee N, Chamberlain L: **Neuroimaging and psychophysiological measurement in organizational research: an agenda for research in organizational cognitive neuroscience**. *Ann N Y Acad Sci* 2007, **1118**: 18-42. doi: 10.1196/annals.1412.003.
- Leung L: **Incidental findings in neuroimaging: ethical and medicolegal considerations**. *Neurosci J* 2013, **439145**: 1-7. doi:10.1155/2013/439145.
- Lifshitz M, Margulies DS, Raz A: **Lengthy and expensive? why the future of diagnostic neuroimaging may be faster, cheaper, and more collaborative than we think**. *AJOB Neurosci* 2012, **3**(4): 48-50. doi: 10.1080/21507740.2012.721466.
- Linden DE: **The challenges and promise of neuroimaging in psychiatry**. *Neuron* 2012, **73**(1): 8-22. doi: 10.1016/j.neuron.2011.12.014.
- McCabe DP, Castel AD: **Seeing is believing: the effect of brain images on judgments of scientific reasoning**. *Cognition* 2008, **107**(1): 343-352. doi: 10.1016/j.cognition.2007.07.017.
- Meegan DV: **Neuroimaging techniques for memory detection: scientific, ethical, and legal issues**. *Am J Bioeth* 2008, **8**(1): 9-20. doi: 10.1080/15265160701842007.
- Metlzer CC et al.: **Guidelines for the ethical use of neuroimages in medical testimony: report of a multidisciplinary consensus conference**. AJNR Am *J Neuroradiol* 2014, **35**(4): 632-637. doi: 10.3174/ajnr.A3711.
- Moosa E: **Translating neuroethics: reflections from Muslim ethics: commentary on “ethical concepts and future challenges of neuroimaging: an Islamic perspective”**. *Sci Eng Ethics* 2012, **18**(3): 519-528. doi: 10.1007/s11948-012-9392-5.
- Morgan A: **Representations gone mental**. *Synthese* 2014, **191**(2): 213-244. doi: 10.1007/s11229-013-0328-7.
- O’Connell G et al.: **The brain, the science and the media: the legal, corporate, social and security implications of neuroimaging and the impact of media coverage**. *EMBO Rep* 2011, **12**(7): 630-636. doi: 10.1038/embor.2011.115.
- Parens E, Johnston J: **Does it make sense to speak of neuroethics? three problems with keying ethics to hot new science and technology**. *EMBO Rep* 2007, **8**:S61-S64. doi: 10.1038/si.embor.7400992.
- Parens E, Johnston J: **Neuroimaging: beginning to appreciate its complexities**. *Hastings Cent Rep* 2014, **44**(2): S2-S7. doi: 10.1002/hast.293.
- Peterson A et al.: **Assessing decision-making capacity in the behaviorally nonresponsive patient with residual covert awareness**. *AJOB Neurosci* 2013, **4**(4): 3-14. doi: 10.1080/21507740.2013.821189.
- Pixten W, Nys H, Dierickx K: **Ethical and regulatory issues in pediatric research supporting the non-clinical application of fMR imaging**. *Am J Bioeth* 2009, **9**(1): 21-23. doi: 10.1080/15265160802627018.
- Poldrack RA, Gorgolewski KJ: **Making big data open: data sharing in neuroimaging**. *Nat Neurosci* 2014, **17**(11): 1510-1517. doi: 10.1038/nn.3818.
- Racine E et al.: **A Canadian perspective on ethics review and neuroimaging: tensions and solutions**. *Can J Neurol Sci* 2011, **38**(4): 572-579. doi: 10.1017/S0317167100012117.
- Racine E, Illes J: **Emerging ethical challenges in advanced neuroimaging research: review, recommendations and research agenda**. *J Empir Res Hum Res Ethics* 2007, **2**(2): 1-10. doi: 10.1525/jer.2007.2.2.1.
- Racine E, Bar-llan O, Illes J: **fMRI in the public eye***. Nat Rev Neurosci* 2005, **6**(2): 159-164. doi: 10.1038/nrn1609.
- Racine E, Bar-llan O, Illes J: **Brain imaging – a decade of coverage in the print media**. *Sci Commun* 2006, **28**(1): 122-143. doi: 10.1177/1075547006291990.
- Ramos RT: **The conceptual limits of neuroimaging in psychiatric diagnosis**. *AJOB Neurosci* 2012, **3**(4): 52-53. doi: 10.1080/21507740.2012.721856.
- Robert JS: **Gene maps, brain scans, and psychiatric nosology**. *Camb Q Healthc Ethics* 2007, **16**(2): 209-218. doi: 10.1017/S0963180107070223.
- Rodrique C, Riopelle RJ, Bernat J, Racine E: **Perspectives and experience of healthcare professionals on diagnosis, prognosis, and end-of-life decision making in patients with disorders of consciousness.***Neuroethics* 2013, **6**(1): 25-36. doi: 10.1007/s12152-011-9142-4.
- Roskies AL: **Neuroimaging and inferential distance**. *Neuroethics* 2008, **1**(1): 19-30. doi: 10.1007/s12152-007-9003-3.
- Rusconi E, Mitchener-Nissen T: **The role of expectations, hype and ethics in neuroimaging and neuromodulation futures**. *Front Syst Neurosci* 2014, **8**: 214. doi: 10.3389/fnsys.2014.00214.
- Sample M: **Evolutionary, not revolutionary: current prospects for diagnostic neuroimaging**. *AJOB Neurosci* 2012, **3**(4): 46-48. doi: 10.1080/21507740.2012.721461.
- Samuel G: “Popular demand”—constructing an imperative for fMRI. *AJOB Neurosci* 2013, 4(4): 17-18. doi: 10.1080/21507740.2013.827280.
- Scott NA, Murphy TH, Illes J: **Incidental findings in neuroimaging research: a framework for anticipating the next frontier**. *J Empir Res Hum Res Ethics* 2012, **7**(1): 53-57. doi: 10.1525/jer.2012.7.1.53.
- Seixas D, Basto MA: **Ethics in fMRI studies: a review of the EMBASE and MEDLINE literature**. *Clin Neuroradiol* 2008, **18**(2): 79-87. doi: 10.1007/s00062-008-8009-5.
- Shaw RL et al.: **Ethical issues in neuroimaging health research: an IPA study with research participants**. *J Health Psychol* 2008, **13**(8): 1051-1059. doi: 10.1177/1359105308097970.
- Stevenson DK, Goldworth A: **Ethical considerations in neuroimaging and its impact on decision-making for neonates**. *Brain Cogn* 2002, **50**(3): 449-454. doi:10.1016/S0278-2626(02)00523-7.
- Synofzik M: **Was passiert im Gehirn meines Patienten? Neuroimaging und Neurogenetik als ethische Herausforderungen in der Medizin. [What happens in the brain of my patients? neuroimaging and neurogenetics as ethical challenges in medicine.]***Dtsch Med Wochenschr* 2007, **132**(49), 2646-2649. doi:10.1055/s-2007-993114.
- Turner DC, Sahakian BJ: **Ethical questions in functional neuroimaging and cognitive enhancement**. *Poiesis Prax* 2006, **4**:81-94. doi: 10.1007/s10202-005-0020-1.
- van Hooff JC: **Neuroimaging techniques for memory detection: scientific, ethical, and legal issues**. *Am J Bioeth* 2008, **8**(1): 25-26. doi: 10.1080/15265160701828501.
- Valerio J, Illes J: **Ethical implications of neuroimaging in sports concussion**. *J Head Trauma Rehabil* 2012, **27**(3): 216-221. doi: 10.1097/HTR.0b013e3182229b6c.
- Vincent NA: **Neuroimaging and responsibility assessments**. *Neuroethics* 2011, **4**(1): 35-49. doi: 10.1007/s12152-008-9030-8.
- Wardlaw JM**: “Can it read my mind?” – what do the public and experts think of the current (mis)uses of neuroimaging?***PLoS One* 2011, **6**(10): e25829. doi: 10.1371/journal.pone.0025829.
- Wasserman D, Johnston J: **Seeing responsibility: can neuroimaging teach us anything about moral and legal responsibility?***Hastings Cent Rep* 2014, **44** (s2): S37-S49. doi:10.1002/hast.297.
- Weijer C et al.: **Ethics of neuroimaging after serious brain injury**. *BMC Med Ethics* 2014, **15**:41. doi: 10.1186/1472-6939-15-41.
- White T et al.: **Pediatric population-based neuroimaging and the Generation R study: the intersection of developmental neuroscience and epidemiology**. *Eur J Epidemiol* 2013, **28**(1): 99-111. doi: 10.1007/s10654-013-9768-0.
- Whiteley L: **Resisting the revelatory scanner? critical engagements with fMRI in popular media**. *Biosocieties* 2012, **7**(3): 245-272. doi: 10.1057/biosoc.2012.21.
- Wu KC: **Soul-making in neuroimaging?***Am J Bioeth* 2008, **8**(9): 21-22. doi: 10.1080/15265160802412536.
- Zarzeczny A, Caulfield T: **Legal liability and research ethics boards: the case of neuroimaging and incidental findings**. *Int J Law Psychiatry* 2012, **35**(2): SI137-SI145. doi: 10.1016/j.ijlp.2011.12.005.

Books:

- Demertzi A: *Ain’t No Rest for the Brain: Neuroimaging and Neuroethics in Dialogue for Patients with Disorders of Consciousness*. Saarbr̈ucken, Germany: LAP Lambert Academic Publishing; 2012.
- Dumit J: *Picturing Personhood: Brain Scans and Biomedical Identity*. Princeton, NJ: Princeton University Press; 2004.
- Joyce KA: *Magnetic Appeal: MRI and the Myth of Transparency*. Ithaca, NY: Cornell University Press; 2008.
- Lanzerath D, Rietschel M, Heinrichs B, Schmäl C, Baldwin T: *Incidental Findings: Scientific, Legal and Ethical Issues*. Köln : Deutscher Ärzte-Verlag; 2014.
- Richmond S, Rees G, Edwards SJL: *I Know What You’re Thinking: Brain Imaging and Mental Privacy*. Oxford: Oxford University Press; 2012.

Book Chapters:

- Arentshorst ME, Broerse JEW, Roelofsen A, de Cock Buning T: **Towards responsible neuroimaging applications in health care: guiding visions of scientists and technology developers**. In *Responsible Innovation 1: Innovative Solutions for Global Issues*. Edited by Jeroen van den Hoven. Dordrecht: Springer; 2014: 255-280.
- Canli T, Amin Z: **Neuroimaging of emotion and personality: ethical considerations**. In *Neuroethics: An Introduction with Readings*. Edited by Martha J. Farah. Cambridge, Mass.: MIT Press; 2010:147-154.
- Canli T: **When genes and brains unite: ethical implications of genomic neuroimaging**. In *Neuroethics: Defining the Issues in Theory, Practice and Policy*. Edited by Judith Illes. Oxford: Oxford University Press; 2006: 169-184.
- Farrah MJ, Gillihan SJ: **Neuroimaging in clinical psychiatry**. In *Neuroethics in Practice*. Edited by Anjan Chatterjee, Martha J. Farah. New York: Oxford University Press; 2013: 128-148.
- Frederico C, Lombera S, Illes J: **Intersecting complexities in neuroimaging and neuroethics**. In *The Oxford Handbook of Neuroethics*. Edited by Judy Illes, Barbara J. Sahakian. Oxford: Oxford University Press; 2011: 377-387.
- Hadskis MR, Schmidt MH: **Pediatric neuroimaging research**. In *The Oxford Handbook of Neuroethics*. Edited by Judy Illes, Barbara J. Sahakian. Oxford: Oxford University Press; 2011:389-404.
- Illes J et al.: **Ethical and practical considerations in managing incidental findings in functional magnetic resonance imaging**. In *Defining Right and Wrong in Brain Science: Essential Readings in Neuroethics*. Edited by Walter Glannon. New York: Dana Press; 2007: 104-114.
- Illes J, Racine E: **Imaging or imagining? A neuroethics challenge informed by genetics**. In *Defining Right and Wrong in Brain Science: Essential Readings in Neuroethics*. Edited by Walter Glannon. New York: Dana Press; 2007: 140-162.
- Illes J: **Neuroethics in a new era of neuroimaging**. In *Defining Right and Wrong in Brain Science: Essential Readings in Neuroethics*. Edited by Walter Glannon. New York: Dana Press; 2007: 99-103.
- Illes J, Rosen A, Greicius M, Racine E: **Prospects for prediction: ethics analysis of neuroimaging in Alzheimer’s disease.** In *Imaging and the Aging Brain*. Edited by Mony J. de Leon, Donald A. Snider, Howard Federoff. Boston: Blackwell; 2007: 278-295.
- Kahlaoui K et al.: **Neurobiological and neuroethical perspectives on the contribution of functional neuroimaging to the study of aging in the brain**. In *The Oxford Handbook of Neuroethics*. Edited by Judy Illes, Barbara J. Sahakian. Oxford: Oxford University Press; 2011: 495-512.
- Karanasion IS, Biniaris CG, Marsh AJ: **Ethical issues of brain functional imaging: reading your mind**. In *Medical and Care Compunetics 5*. Edited by Lodewijk Bos. Amsterdam: IOS Press; 2008: 310-320.
- Koch T: **Images of uncertainty: two cases of neuroimages and what they cannot show**. In *Neurotechnology: Premises, Potential, and Problems*. Edited by James Giordano. Boca Raton: CRC Press; 2012: 47-57.
- Kulynych J: **Legal and ethical issues in neuroimaging research: human subjects protection, medical privacy, and the public communication of research results**. In *Defining Right and Wrong in Brain Science: Essential Readings in Neuroethics*. Edited by Walter Glannon. New York: Dana Press; 2007: 115-133.
- Mamourian A: **Incidental findings on research functional MR images: should we look?** In *Defining Right and Wrong in Brain Science: Essential Readings in Neuroethics*. Edited by Walter Glannon. New York: Dana Press; 2007: 134-139.
- Owen AM: **Functional magnetic resonance imaging, covert awareness, and brain injury**. In *The Oxford Handbook of Neuroethics*. Edited by Judy Illes, Barbara J. Sahakian. Oxford: Oxford University Press; 2011:135-147.
- Phelps EA, Thomas LA: **Race, behavior, and the brain: the role of neuroimaging in understanding complex social behaviors**. In *Neuroethics: An Introduction with Readings*. Edited by Martha J. Farah. Cambridge, Mass.: MIT Press; 2010: 191-200.
- Racine E, Bell E, Illes J: **Can we read minds? ethical challenges and responsibilities in the use of neuroimaging research**. In *Scientific and Philosophical Perspectives in Neuroethics*. Edited by James J. Giordano, Bert Gordijn. Cambridge: Cambridge University Press; 2010: 244-270.
- Raschle N et al.: **Pediatric neuroimaging in early childhood and infancy: challenges and practical guidelines**. In *The Neurosciences and Music IV: Learning and Memory*. Edited by Katie Overy. Boston, Mass.: Blackwell; 2012: 43-50.
- Toole C, Zarzeczny A, Caulfield T: **Research ethics challenges in neuroimaging research: a Canadian perspective**. In *International Neurolaw: A Comparative Analysis*. Edited by Tade Matthias Spranger. Berlin: Springer; 2012: 89-101.
- Ulmer S et al.: **Incidental findings in neuroimaging research: ethical considerations**. In *fMRI: Basics and Clinical Applications*. Edited by Stephan Ulmer, Olav Jansen. Berlin: Springer; 2013: 311-318.
- Vanmeter J: **Neuroimaging: thinking in pictures**. In *Scientific and Philosophical Perspectives in Neuroethics*. Edited by James J. Giordano, Bert Gordijn. Cambridge: Cambridge University Press; 2010: 230-243.

***Neurogenetics:***

- **Choosing deafness**. *Arch Dis Child* 2003, **88**(1):24.
- Abreu Alves FR, Quintanilha Ribeiro Fde A: **Diagnosis routine and approach in genetic sensorineural hearing loss**. *Braz J Otorhinolaryngol* 2007, **73**(3):412-417.
- Alcalay RN et al.: **Michael J. Fox Foundation LRRK2 Consortium: geographical differences in returning genetic research data to study participants**. *Genet Med* 2014, **16**(8):644-645. doi:10.1038/gim.2014.55.
- Alonso ME et al.: **Homozygosity in Huntington's disease: new ethical dilemma caused by molecular diagnosis**. *Clin Genet* 2002, **61**(6):437-442.
- Anido A, Carlson LM, Sherman SL: **Attitudes toward Fragile X mutation carrier testing from women identified in a general population survey**. *J Genet Couns* 2007, **16**(1):97-104. doi:10.1007/s10897-006-9049-0.
- Arnos KS: **The implications of genetic testing for deafness**. *Ear Hear* 2003, **24**(4):324-331. doi:10.1097/01.AUD.0000079800.64741.CF.
- Arnos KS: **Ethical and social implications of genetic testing for communication disorders**. *J Commun Disord* 2008, **41**(5):444-457. doi:10.1016/j.jcomdis.2008.03.001.
- Asscher E, Koops BJ: **The right not to know and preimplantation genetic diagnosis for Huntington's disease**. *J Med Ethics* 2010, **36**(1):30-33. doi:10.1136/jme.2009.031047.
- Avard DM, Knoppers BM: **Ethical dimensions of genetics in pediatric neurology: a look into the future**. *Semin Pediatr Neurol* 2002, **9**(1):53-61.
- Barrett SK, Drazin T, Rosa D, Kupchik GS: **Genetic counseling for families of patients with Fragile X syndrome**. *JAMA* 2004, **291**(24): 2945. doi:10.1001/jama.291.24.2945-a.
- Bassett SS, Havstad SL, Chase GA: **The role of test accuracy in predicting acceptance of genetic susceptibility testing for Alzheimer's disease**. *Genet Test* 2004, ***8***(2):120-126. doi:10.1089/1090657041797383.
- Bechtel K, Geschwind MD: **Ethics in prion disease**. *Prog Neurobiol* 2013, **110**:29-44. doi:10.1016/j.pneurobio.2013.07.001.
- Blasé T et al.: **Sharing GJB2/GJB6 genetic test information with family members**. *J Genet Couns* 2007, **16**(3):313-324. doi:10.1007/s10897-006-9066-z.
- Bombard Y et al.: **Beyond the patient: the broader impact of genetic discrimination among individuals at risk of Huntington disease**. *Am J Med Genet B Neuropsychiatr Genet* 2012, **159B**(2):217-226. doi:10.1002/ajmg.b.32016.
- Bombard Y et al.: **Factors associated with experiences of genetic discrimination among individuals at risk for Huntington disease**. *Am J Med Genet B Neuropsychiatr Genet* 2011, **156B**(1):19-27. doi:10.1002/ajmg.b.31130.
- Bombard Y et al.: **Engagement with genetic discrimination: concerns and experiences in the context of Huntington disease**. *Eur J Hum Genet* 2008, **16**(3): 279-289. doi: 10.1038/sj.ejhg.5201937.
- Bombard Y, Semaka A, Hayden MR: **Adoption and the communication of genetic risk: experiences in Huntington disease**. *Clin Genet* 2012, **81**(1), 64-69. doi:10.1111/j.1399-0004.2010.01614.x.
- Borry P, Clarke A, Dierickx K: **Look before you leap: carrier screening for type 1 Gaucher disease: difficult questions**. *Eur J Hum Genet* 2008, **16**(2)139-140. doi: 10.1038/sj.ejhg.5201960.
- Boudreault P et al.: **Deaf adults' reasons for genetic testing depend on cultural affiliation: results from a prospective, longitudinal genetic counseling and testing study**. *J Deaf Stud Deaf Educ* 2010, **15**(3):209-227. doi:10.1093/deafed/enq012.
- Breakefield XO, Sena-Esteves M: **Healing genes in the nervous system**. *Neuron* 2010, **68**(2):178-181. doi:10.1016/j.neuron.2010.10.005.
- Brief E, Illes J: **Tangles of neurogenetics, neuroethics, and culture**. *Neuron* 2010, **68**(2):174-177. doi:10.1016/j.neuron.2010.09.041.
- Buchman DZ, Illes J: **Imaging genetics for our neurogenetic future**. *Minn J L Sci & Tech* 2010, **11**(1):79-97.
- Burton SK et al.: **A focus group study of consumer attitudes toward genetic testing and newborn screening for deafness**. *Genet Med* 2006, **8**(12): 779-783. doi:10.109701.gim.0000250501.59830.ff.
- Cabanillas Farpón R, Cadinanos Banales J: **Hereditary hearing loss: Genetic counselling**. [Hipoacusias hereditarias: asesoramiento genetico] *Acta Otorrinolaringol Esp* 2012, **63**(3):218-229. doi:10.1016/j.otorri.2011.02.006.
- Campbell E, Ross LF: **Parental attitudes regarding newborn screening of PKU and DMD**. *Am J Med Genet A* 2003, **120A**(2):209-214. doi:10.1002/ajmg.a.20031.
- Camporesi S: **Choosing deafness with preimplantation genetic diagnosis: an ethical way to carry on a cultural bloodline?***Camb Q Healthc* 2010, **19**(1):86-96. doi:10.1017/S0963180109990272.
- Caron L et al.: **Nicotine addiction through a neurogenomic prism: ethics, public health, and smoking**. *Nicotine Tob Res* 2005, **7**(2): 181-197. doi:10.1080/14622200500055251.
- Chen DT et al.: **Impact of restricting enrollment in stroke genetics research to adults able to provide informed consent**. *Stroke* 2008, **39**(3):831-837. doi:10.1161/STROKEAHA.107.494518.
- Chen DT et al.: **Stroke genetic research and adults with impaired decision-making capacity: a survey of IRB and investigator practices**. *Stroke* 2008, **39**(10): 2732-2735. doi:10.1161/STROKEAHA.108.515130.
- Chen DT et al.: **The impact of privacy protections on recruitment in a multicenter stroke genetics study**. *Neurology* 2005, **64**(4): 721-724. doi: 10.1212/01.WNL.0000152042.07414.CC.
- Cleary-Goldman J et al.: **Screening for Down syndrome: practice patterns and knowledge of obstetricians and gynecologists**. *Obstet Gynecol* 2006, **107**(1): 11-17. doi: 10.1097/01.AOG.0000190215.67096.90.
- Corcia P: **Methods of the announcement of amyotrophic lateral sclerosis diagnosis in familial forms**. [Contenu et modalites de l'annonce du diagnostic de SLA dans un contexte familial]. *Rev Neurol (Paris)* 2006, **162 Spec No 2**, 4S122-4S126. doi:MDOI-RN-06-2006-162-HS2-0035-3787-101019-200509367.
- Czeisler CA: **Medical and genetic differences in the adverse impact of sleep loss on performance: ethical considerations for the medical profession**. *Trans Am Clin Climatol Assoc* 2009, **120**:249-285.
- de Die-Smulders CE et al.: **Reproductive options for prospective parents in families with Huntington's disease: clinical, psychological and ethical reflections**. *Hum Reprod Update* 2013, **19**(3):304-315. doi:10.1093/humupd/dms058.
- de Luca D et al.: **Heterologous assisted reproduction and kernicterus: the unlucky coincidence reveals an ethical dilemma**. *J Matern Fetal Neonatal Med* 2008, **21**(4): 219-222. doi:10.1080/14767050801924811.
- Decruyenaere M et al.: **The complexity of reproductive decision-making in asymptomatic carriers of the Huntington mutation**. *Eur J Hum Genet* 2007, **15**(4): 453-462. doi: 10.1038/sj.ejhg.5201774.
- Dekkers W, Rikkert MO: **What is a genetic cause? the example of Alzheimer's disease**. *Med Health Care Philos* 2006, **9**(3): 273-284. doi:10.1007/s11019-006-9005-7.
- Dennis C: **Genetics: deaf by design**. *Nature* 2004, **431**(7011):894-896. doi: 10.1038/431894a.
- Diniz D: **Reproductive autonomy: a case study on deafness [Autonomia reprodutiva: um estudo de caso sobre a surdez]**. *Cad Saude Publica* 2003, **19**(1):175-181.
- Donaldson ZR, Young LJ: **Oxytocin, vasopressin, and the neurogenetics of sociality**. *Science* 2008, **322**(5903):900-904. doi: 10.1126/science.1158668.
- Dorsey ER et al.: **Knowledge of the Genetic Information Nondiscrimination Act among individuals affected by Huntington disease**. *Clin Genet* 2013, **84**(3): 251-257. doi:10.1111/cge.12065.
- Duncan RE et al.: **"You're one of us now": young people describe their experiences of predictive genetic testing for Huntington disease (HD) and Familial Adenomatous Polyposis (FAP)**. *Am J Med Genet C Semin Med Genet* 2008, **148C**(1): 47-55. doi:10.1002/ajmg.c.30158.
- Duncan RE et al.: **An international survey of predictive genetic testing in children for adult onset conditions**. *Genet Med* 2005, **7**(6): 390-396. doi: 10.109701.GIM.0000170775.39092.44.
- Edge K: **The benefits and potential harms of genetic testing for Huntington's disease: a case study**. *Hum Reprod Genet Ethics* 2008, **14**(2):14-19. doi:10.1558/hrge.v14i2.14.
- Eggert K et al.: **Data protection in biomaterial banks for Parkinson's disease research: the model of GEPARD (Gene Bank Parkinson's Disease Germany)**. *Mov Disord* 2007, **22**(5): 611-618. doi:10.1002/mds.21331.
- Eisen A et al.: **SOD1 gene mutations in ALS patients from British Columbia, Canada: clinical features, neurophysiology and ethical issues in management**. *Amyotroph Lateral Scler* 2008, **9**(2): 108-119. doi:10.1080/17482960801900073.
- Erez A Plunkett K, Sutton VR, McGuire AL: **The right to ignore genetic status of late onset genetic disease in the genomic era: prenatal testing for Huntington disease as a paradigm.***Am J Med Genet A* 2010, **152A**(7): 1774-1780. doi:10.1002/ajmg.a.33432.
- Erwin C Hersch S, Event Monitoring Committee of the Huntington Study Group: **Monitoring reportable events and unanticipated problems: the PHAROS and PREDICT studies of Huntington disease**. *IRB* 2007, **29**(3): 11-16.
- Erwin C et al.: **Perception, experience, and response to genetic discrimination in Huntington disease: the international RESPOND-HD study**. *Am J Med Genet B Neuropsychiatr Genet* 2010, **153B**(5): 1081-1093. doi:10.1002/ajmg.b.31079.
- Etchegary H: **Discovering the family history of Huntington disease**. *J Genet Couns* 2006, **15**(2): 105-117. doi:10.1007/s10897-006-9018-7.
- Fahmy MS: **On the supposed moral harm of selecting for deafness**. *Bioethics* 2011, **25**(3):128-136. doi:10.1111/j.1467-8519.2009.01752.x.
- Fanos JH, Gelinas DF, Miller RG: **"You have shown me my end": attitudes toward presymptomatic testing for familial Amyotrophic Lateral Sclerosis**. *Am J Med Genet A* 2004, **129A**(3):248-253. doi:10.1002/ajmg.a.30178.
- Fins JJ: **"Humanities are the hormones:" Osler, Penfield and "neuroethics" revisited**. *Am J Bioeth* 2008, **8**(1):W5-8. doi:10.1080/15265160801891227.
- Finucane B, Haas-Givler B, Simon EW: **Genetics, mental retardation, and the forging of new alliances**. *Am J Med Genet C Semin Med Genet* 2003, **117C**(1):66-72. doi:10.1002/ajmg.c.10021.
- Forrest Keenan K et al.: **How young people find out about their family history of Huntington's disease**. *Soc Sci Medi* 2009, **68**(10):1892-1900. doi:10.1016/j.socscimed.2009.02.049.
- Fu S, Dong J, Wang C, Chen G: **Parental attitudes toward genetic testing for prelingual deafness in China**. *Int J Pediatr Otorhinolaryngol* 2010, **74**(10):1122-1125. doi:10.1016/j.ijporl.2010.06.012.
- Fukushima Y: [**Pediatric neurological disorders and genetic counseling**.] *No to Hattatsu* 2003, **35**(4): 285-291.
- Gillam L, Poulakis Z, Tobin S, Wake M: **Enhancing the ethical conduct of genetic research: investigating views of parents on including their healthy children in a study on mild hearing loss**. *J Med Ethics* 2006, **32**(9):537-541. doi: 10.1136/jme.2005.013201.
- Giordano J: **Neuroethical issues in neurogenetic and neuro-implantation technology: the need for pragmatism and preparedness in practice and policy**. *Stud Ethics Law and Technol* 2011, **4**(3). doi:10.2202/1941-6008.1152.
- Godard B, Cardinal G: **Ethical implications in genetic counseling and family studies of the epilepsies**. *Epilepsy Behav* 2004, **5**(5):621-626. doi:10.1016/j.yebeh.2004.06.016.
- Goh AM et al.: **Perception, experience, and response to genetic discrimination in Huntington's disease: the Australian results of the International RESPOND-HD study**. *Genet Test Mol Biomarkers* 2013, **17**(2):115-121. doi:10.1089/gtmb.2012.0288.
- Goldman JS, Hou CE: **Early-onset Alzheimer disease: when is genetic testing appropriate?***Alzheimer Dis Assoc Disord* 2004, **18**(2): 65-67.
- Golomb MR, Garg BP, Walsh LE, Williams LS: **Perinatal stroke in baby, prothrombotic gene in mom: does this affect maternal health insurance?***Neurology* 2005, **65**(1):13-16. doi:10.1212/01.wnl.0000167543.83897.fa.
- Goodey CF: **On certainty, reflexivity and the ethics of genetic research into intellectual disability**. *J Intellect Disabil Res* 2003, **47**(Pt 7):548-554.
- Gordon SC, Landa D: **Disclosure of the genetic risk of Alzheimer's disease**. *N Engl J Med* 2010, **362**(2):181-2. doi:10.1056/NEJMc096300.
- Grant R, Flint K: **Prenatal screening for fetal aneuploidy: a commentary by the Canadian Down Syndrome Society**. *J Obstet Gynaecol Can* 2007, **29**(7):580-582.
- Green RC et al.: **Disclosure of APOE genotype for risk of Alzheimer's disease**. *N Engl J Med* 2007, **361**(3):245-254. doi:10.1056/NEJMoa0809578.
- Gross ML: **Ethics, policy, and rare genetic disorders: the case of Gaucher disease in Israel**. *Theor Med Bioeth* 2002, **23**(2):151-170.
- Guillemin M, Gillam L: (2006). **Attitudes to genetic testing for deafness: the importance of informed choice**. *J Genet Couns* 2006, **15**(1):51-59. doi:10.1007/s10897-005-9003-6.
- Guzauskas GF, Lebel RR: **The duty to re-contact for newly appreciated risk factors: Fragile X premutation**. *J Clin Ethics* 2006, **17**(1):46-52.
- Harper PS et al.: **Genetic testing and Huntington's disease: issues of employment**. *Lancet Neurol* 2004, **3**(4):249-252. doi:10.1016/S1474-4422(04)00711-2.
- Harris JC: **Advances in understanding behavioral phenotypes in neurogenetic syndromes**. *Am J Medical Genet C Semin Med Genet* 2010, **154C**(4): 389-399. doi:10.1002/ajmg.c.30276.
- Hassan A, Markus HS: **Practicalities of genetic studies in human stroke**. *Methods Mol Med* 2005, **104**:223-240. doi: 10.1385/1-59259-836-6:223.
- Hawkins AK, Ho A, Hayden MR: **Lessons from predictive testing for Huntington disease: 25 years on**. *J Med Genet* 2011, **48**(10):649-650. doi:10.1136/jmedgenet-2011-100352.
- Haworth A et al.: **Call for participation in the neurogenetics consortium within the Human Variome Project**. *Neurogenetics* 2011, **12**(3):169-73. doi: 10.1007/s10048-011-0287-4.
- Hayry M: **There is a difference between selecting a deaf embryo and deafening a hearing child**. *J Med Ethics* 2004, **30**(5):510-512. doi: 10.1136/jme.2002.001891.
- Hipps YG, Roberts JS, Farrer LA, Green RC: **Differences between African Americans and whites in their attitudes toward genetic testing for Alzheimer's disease**. *Genet Test* 2003, **7**(1):39-44. doi:10.1089/109065703321560921.
- Holland A, Clare IC: **The Human Genome Project: considerations for people with intellectual disabilities**. *J Intellect Disabil Res* 2003, **47**(Pt 7): 515-525. doi: 10.1046/j.1365-2788.2003.00530.x.
- Holt K: **What do we tell the children? Contrasting the disclosure choices of two HD families regarding risk status and predictive genetic testing**. *J Genet Couns* 2006, **15**(4):253-265. doi:10.1007/s10897-006-9021-z.
- Hoop JG, Spellecy R: **Philosophical and ethical issues at the forefront of neuroscience and genetics: an overview for psychiatrists**. *Psychiatr Clin North Am* 2009, **32**(2): 437-449. doi:10.1016/j.psc.2009.03.004.
- Horiguchi T, Kaga M, Inagaki M: **[Assessment of chromosome and gene analysis for the diagnosis of the fragile X syndrome in Japan: annual incidence.]***No To Hattatsu* 2005, **37**(4):301-306.
- Huniche L: **Moral landscapes and everyday life in families with Huntington's disease: aligning ethnographic description and bioethics**. *Soc Sci Med* 2011, **72**(11):1810-1816. doi:10.1016/j.socscimed.2010.06.039.
- Hurley AC et al.: **Genetic susceptibility for Alzheimer's disease: why did adult offspring seek testing?***Am J Alzheimers Dis Other Demen* 2005, **20**(6):374-381.
- Illes F et al.: **Einstellung zu genetischen untersuchungen auf Alzheimer-Demenz [Attitudes towards predictive genetic testing for Alzheimer's disease.]***Z Gerontol Geriatr* 2006, **39**(3): 233-239. doi:10.1007/s00391-006-0377-3.
- Inglis A, Hippman C, Austin JC: **Prenatal testing for Down syndrome: the perspectives of parents of individuals with Down syndrome**. *Am J Med Genet A* 2012, **158A**(4): 743-750. doi:10.1002/ajmg.a.35238.
- Johnston T: **In one's own image: ethics and the reproduction of deafness**. *J Deaf Stud Deaf Educ* 2005, **10**(4):426-441. doi: 10.1093/deafed/eni040.
- Kane RA, Kane RL: **Effect of genetic testing for risk of Alzheimer's disease**. *N Engl J Med* 2009, **361**(3):298-299. doi:10.1056/NEJMe0903449.
- Kang PB: **Ethical issues in neurogenetic disorder**. *Handb Clin Neurol* 2013, **118**:265-276. doi: 10.1016/B978-0-444-53501-6.00022-6.
- Kim SY et al.: **Volunteering for early phase gene transfer research in Parkinson disease**. *Neurology* 2006, **66**(7):1010-1015. doi: 10.1212/01.wnl.0000208925.45772.ea
- King NM: **Genes and Tourette syndrome: scientific, ethical, and social implications**. *Adv Neurol* 2006, **99**:144-147.
- Kissela BM et al.: **Proband race/ethnicity affects pedigree completion rate in a genetic study of ischemic stroke**. *J Stroke Cerebrovasc Dis* 2008, **17**(5):299-302. doi:10.1016/j.jstrokecerebrovasdis.2008.02.011.
- Klein C, Ziegler A: **From GWAS to clinical utility in Parkinson's disease**. *Lancet* 2011, **377**(9766):613-614. doi:10.1016/S0140-6736(11)60062-7.
- Klein CJ, Dyck PJ: **Genetic testing in inherited peripheral neuropathies**. *J Peripher Nerv Syst* 2005, **10**(1):77-84. doi: 10.1111/j.1085-9489.2005.10111.x.
- Klitzman R et al.: **Decision-making about reproductive choices among individuals at-risk for Huntington's disease**. *J Genet Couns* 2007, **16**(3):347-362. doi:10.1007/s10897-006-9080-1.
- Krajewski KM, Shy ME: **Genetic testing in neuromuscular disease**. *Neurol Clin* 2004, **22**(3):481-508. doi:10.1016/j.ncl.2004.03.003.
- Kromberg JG, Wessels TM: **Ethical issues and Huntington's disease**. *S Afr Med J* 2013, **103**(12 Suppl 1):1023-1026. doi:10.7196/samj.7146.
- Kullmann DM, Schorge S, Walker MC, Wykes RC: **Gene therapy in epilepsy-is it time for clinical trials?***Nat Rev Neurol* 2014, **10**(5):300-304. doi:10.1038/nrneurol.2014.43.
- Labrune P: **Diagnostic genetique pre-implantatoire de la choree de Huntington sans savoir si le parent est attaint. [Pre-implantation genetic diagnosis of Huntington's chorea without disclosure if the parent "at risk" is affected.]***Arch Pediatr* 2003, **10**(2):169-170.
- Laney DA et al.: **Fabry Disease practice guidelines: recommendations of the National Society of Genetic Counselors**. *J Genet Couns* 2013, **22**(5):555-564. doi: 10.1007/s10897-013-9613-3
- LaRusse S et al.: **Genetic susceptibility testing versus family history-based risk assessment: impact on perceived risk of Alzheimer disease**. *Genet Med* 2005, **7**(1):48-53. doi: 10.109701.GIM.0000151157.13716.6C.
- Le Hellard S, Hanson I: **The Imaging and Cognition Genetics Conference 2011, ICG 2011: a meeting of minds**. *Front Neurosci* 2012, **6**:74 doi: 10.3389/fnins.2012.00074.
- Leuzy A, Gauthier S: **Ethical issues in Alzheimer's disease: an overview**. *Expert Rev Neurother* 2012, **12**(5):557-567. doi:10.1586/ern.12.38.
- Mand C et al.: **Genetic selection for deafness: the views of hearing children of deaf adults**. *J Med Ethics* 2009, **35**(12):722-728. doi:10.1136/jme.2009.030429.
- Marcheco-Teruel B, Fuentes-Smith E: **Attitudes and knowledge about genetic testing before and after finding the disease-causing mutation among individuals at high risk for familial, early-onset Alzheimer's disease**. *Genet Test Mol Biomarkers* 2009, **13**(1):121-125. doi:10.1089/gtmb.2008.0047.
- Mathews KD: **Hereditary causes of chorea in childhood**. *Semin Pediatr Neurol* 2003, **10**(1):20-25.
- McGrath RJ et al.: **Access to genetic counseling for children with autism, Down syndrome, and intellectual disabilities**. *Pediatrics* 2009, **124 Suppl 4**:S443-449. doi:10.1542/peds.2009-1255Q.
- Meininger HP: **Intellectual disability, ethics and genetics--a selected bibliography**. *J Intellect Disabil Res* 2003, **47**(Pt 7):571-576.
- Meschia JF, Merino JG: **Reporting of informed consent and ethics committee approval in genetics studies of stroke**. *J Med Ethics* 2003, **29**(6):371-372.
- Molnar MJ, Bencsik P: **Establishing a neurological-psychiatric biobank: banking, informatics, ethics**. *Cell Immunol* 2006, **244**(2):101-104. doi: 10.1016/j.cellimm.2007.02.013.
- Moscarillo TJ et al.: **Knowledge of and attitudes about Alzheimer disease genetics: report of a pilot survey and two focus groups**. *Community Genet* 2007, **10**(2): 97-102. 10.1159/000099087.
- Mrazek DA: **Psychiatric pharmacogenomic testing in clinical practice**. *Dialogues Clin Neurosci* 2010, **12**(1): 66-76.
- Munoz-Sanjuan I, Bates GP: **The importance of integrating basic and clinical research toward the development of new therapies for Huntington disease**. *J Clin Invest* 2011, **121**(2):476-483. doi:10.1172/JCI45364.
- Nance WE: **The genetics of deafness**. *Ment Retard Dev Disabil Res Rev* 2003, **9**(2):109-119. doi:10.1002/mrdd.10067.
- Nunes R: **Deafness, genetics and dysgenics**. *Med Health Care Philos* 2006, **9**(1):25-31. doi:10.1007/s11019-005-2852-9.
- Olde Rikkert MG et al.: **Consensus statement on genetic research in dementia**. *Am J Alzheimers Dis Other Demen* 2008, **23**(3):262-266. doi:10.1177/1533317508317817.
- Ottman R, Berenson K, Barker-Cummings C: **Recruitment of families for genetic studies of epilepsy**. *Epilepsia* 2005, **46**(2):290-297. doi: 10.1111/j.0013-9580.2005.41904.x.
- Ottman R et al.: **Genetic testing in the epilepsies--report of the ILAE Genetics Commission**. *Epilepsia* 2010, **51**(4):655-670. doi:10.1111/j.1528-1167.2009.02429.x.
- Paulson HL: **Diagnostic testing in neurogenetics: principles, limitations, and ethical considerations**. *Neurol Clin* 2002, **20**(3):627-643.
- Petrini C: **Guidelines for genetic counselling for neurological diseases: ethical issues**. *Minerva Med* 2011, **102**(2):149-159.
- Poland S: **Intellectual disability, genetics, and ethics: a review**. *Ethics Intellect Disabil* 2004, **8**(1):1-2.
- Ramani D, Saviane C: **Genetic tests: between risks and opportunities: the case of neurodegenerative diseases**. *EMBO Rep* 2010, **11**(12):910-913. doi:10.1038/embor.2010.177.
- Raspberry K, Skinner D: **Enacting genetic responsibility: experiences of mothers who carry the fragile X gene**. *Sociol Health Illn* 2011, **33**(3):420-433. doi:10.1111/j.1467-9566.2010.01289.x.
- Raymond FL: **Genetic services for people with intellectual disability and their families**. *J Intellect Disabil Res* 2003, **47**(7):509-514. doi: 10.1046/j.1365-2788.2003.00529.x.
- Reinders HS: **Introduction to intellectual disability, genetics and ethics**. *J Intellect Disabil Res* 2003*,***47**(7):501-504. doi:
- Reinvang I et al.: **Neurogenetic effects on cognition in aging brains: a window of opportunity for intervention?***Front Aging Neurosci* 2010, **2**:143. doi: 10.1046/j.1365-2788.2003.00527.x10.3389/fnagi.2010.00143.
- Richards FH: **Maturity of judgement in decision making for predictive testing for nontreatable adult-onset neurogenetic conditions: a case against predictive testing of minors.***Clinical Genetics* 2006, **70**(5):396-401. doi: 10.1111/j.1399-0004.2006.00696.x.
- Roberts JS, Chen CA, Uhlmann WR, Green RC: **Effectiveness of a condensed protocol for disclosing APOE genotype and providing risk education for Alzheimer disease**. *Genet Med* 2012, **14**(8):742-748. doi:10.1038/gim.2012.37.
- Roberts JS, Uhlmann WR: **Genetic susceptibility testing for neurodegenerative diseases: ethical and practice issues**. *Prog Neurobiol* 2013, **110**:89-101. doi:10.1016/j.pneurobio.2013.02.005.
- Robins Wahlin TB: **To know or not to know: a review of behaviour and suicidal ideation in preclinical Huntington's disease**. *Patient Educ Couns* 2007, **65**(3): 279-287. doi: 10.1016/j.pec.2006.08.009.
- Rojo A, Corbella C: **Utilidad de los estudios geneticos y de neuroimagen en el diagnostico diferencial de la enfermedad de Parkinson [The value of genetic and neuroimaging studies in the differential diagnosis of Parkinson's disease.]***Rev Neurol* 2009, **48**(9):482-488.
- Romero LJ et al.: **Emotional responses to APO E genotype disclosure for Alzheimer disease**. *J Genet Couns* 2005, **14**(2):141-150. doi:10.1007/s10897-005-4063-1.
- Ryan M, Miedzybrodzka Z, Fraser L, Hall M: **Genetic information but not termination: pregnant women's attitudes and willingness to pay for carrier screening for deafness genes**. *J MedGenet* 2003, **40**(6):e80.
- Savulescu J: **Education and debate: deaf lesbians, "designer disability," and the future of medicine**. *BMJ* 2002, **325**(7367):771-773. doi: 10.1136/bmj.325.7367.771.
- Schanker BD: **Neuroimaging genetics and epigenetics in brain and behavioral nosology**. *AJOB Neurosci* 2012, **3**(4):44-46. doi: 10.1080/21507740.2012.721465.
- Schneider SA, Klein C: **What is the role of genetic testing in movement disorders practice?***Curr Neurol Neurosci Rep* 2011, **11**(4):351-361. doi:10.1007/s11910-011-0200-4.
- Schneider SA, Schneider UH, Klein C: **Genetic testing for neurologic disorders**. *Semin Neurol* 2011, **31**(5):542-552. doi:10.1055/s-0031-1299792.
- Schulze TG, Fangerau H, Propping P: **From degeneration to genetic susceptibility, from eugenics to genethics, from Bezugsziffer to LOD score: the history of psychiatric genetics**. *Int Rev Psychiatry* 2004, **16**(4), 246-259. doi: 10.1080/09540260400014419.
- Semaka A, Creighton S, Warby S, Hayden MR: **Predictive testing for Huntington disease: interpretation and significance of intermediate alleles**. *Clin Genet* 2006, **70**(4):283-294. doi: 10.1111/j.1399-0004.2006.00668.x.
- Semaka A, Hayden MR: **Evidence-based genetic counselling implications for Huntington disease intermediate allele predictive test results**. *Clin Genet* 2014, **85**(4):303-311. doi:10.1111/cge.12324.
- Serretti A, Artioli P: **Ethical problems in pharmacogenetics studies of psychiatric disorders**. *Pharmacogenomics J* 2006, **6**(5): 289-295. doi: 10.1038/sj.tpj.6500388.
- Sevick MA, McConnell T, Muender M: **Conducting research related to treatment of Alzheimer's disease: ethical issues**. *J Gerontol Nurs* 2003, **29**(2):6-12. doi: 10.3928/0098-9134-20030201-05.
- Shekhawat GS et al.: **Implications in disclosing auditory genetic mutation to a family: a case study**. *Int J Audiol* 2007, **46**(7):384-387. doi: 10.1080/14992020701297805.
- Shostak S, Ottman R: **Ethical, legal, and social dimensions of epilepsy genetics**. *Epilepsia* 2006, **47**(10):1595-1602. doi: 10.1111/j.1528-1167.2006.00632.x.
- Smith JA, Stephenson M, Jacobs C, Quarrell O: **Doing the right thing for one's children: deciding whether to take the genetic test for Huntington's disease as a moral dilemma**. *Clin Genet* 2013, **83**(5):417-421. doi:10.1111/cge.12124.
- Synofzik M: **Was passiert im Gehirn meines Patienten? Neuroimaging und Neurogenetik als ethische Herausforderungen in der Medizin. [What happens in the brain of my patients? neuroimaging and neurogenetics as ethical challenges in medicine.]***Dtsch Med Wochenschr* 2007, **132**(49), 2646-2649. doi:10.1055/s-2007-993114.
- Tabrizi SJ, Elliott CL, Weissmann C: **Ethical issues in human prion diseases**. *Br Med Bull* 2003, **66**:305-316.
- Tairyan K, Illes J: **Imaging genetics and the power of combined technologies: a perspective from neuroethics**. *Neuroscience* 2009, **164**(1):7-15. doi:10.1016/j.neuroscience.2009.01.052.
- Tan EC, Lai PS: **Molecular diagnosis of neurogenetic disorders involving trinucleotide repeat expansions**. *Expert Rev Mol Diagn* 2005, **5**(1):101-109. doi: 10.1586/14737159.5.1.101.
- Taneja PR et al.: **Attitudes of deaf individuals towards genetic testing**. *Am J Med Genet A* 2004, **130A**(1):17-21. doi:10.1002/ajmg.a.30051.
- Taylor S: **Gender differences in attitudes among those at risk for Huntington's disease**. *Genet Test* 2005, **9**(2):152-157. doi:10.1089/gte.2005.9.152.
- Taylor SD: **Predictive genetic test decisions for Huntington's disease: context, appraisal and new moral imperatives**. *Soc Sci Med* 2004, **58**(1):137-149. doi: 10.1016/S0277-9536(03)00155-2.
- Toda T: **Personal genome research and neurological diseases: overview**. *Brain Nerve* 2013, **65**(3):227-234.
- Todd RM, Anderson AK: **The neurogenetics of remembering emotions past**. *Proc Nat Acad Sci U S A* 2009, **106**(45):18881-18882. doi: 10.1073/pnas.0910755106.
- Toufexis M, Gieron-Korthals M: **Early testing for Huntington disease in children: pros and cons**. *J Child Neurol* 2010, **25**(4):482-484. doi:10.1177/0883073809343315.
- Towner D, Loewy RS: **Ethics of preimplantation diagnosis for a woman destined to develop early-onset Alzheimer disease**. *JAMA* 2002, **287**(8):1038-1040.
- Valente EM, Ferraris A, Dallapiccola B: **Genetic testing for paediatric neurological disorders**. *Lancet Neurol* 2008, **7**(12):1113-1126. doi:10.1016/S1474-4422(08)70257-6.
- van der Vorm A et al.: **Genetic research into Alzheimer's disease: a European focus group study on ethical issues**. *Int J Geriatr Psychiatry* 2008, **23**(1):11-15. doi: 10.1002/gps.1825.
- van der Vorm A et al.: **Experts' opinions on ethical issues of genetic research into Alzheimer's disease: results of a Delphi study in the Netherlands**. *Clin Genet* 2010, **77**(4):382-388. doi:10.1111/j.1399-0004.2009.01323.x.
- van der Vorm A et al.: **Ethical aspects of research into Alzheimer disease. a European Delphi study focused on genetic and non-genetic research**. *J Med Ethics* 2009, **35**(2):140-144. doi:10.1136/jme.2008.025049.
- Vehmas S: **Is it wrong to deliberately conceive or give birth to a child with mental retardation?***J Med Philos* 2002, **27**(1):47-63. doi:10.1076/jmep.27.1.47.2974.
- Wehbe RM: **When to tell and test for genetic carrier status: perspectives of adolescents and young adults from fragile X families**. *Am J Med Genet A* 2009, **149A**(6):1190-1199. doi:10.1002/ajmg.a.32840.
- Williams JK et al.: **In their own words: reports of stigma and genetic discrimination by people at risk for Huntington disease in the International RESPOND-HD study**. *Am J Medical Genet B Neuropsychiatr Genet* 2010, **153B**(6):1150-1159. doi:10.1002/ajmg.b.31080.
- Withrow KA et al.: **Impact of genetic advances and testing for hearing loss: results from a national consumer survey**. *Am J Med Genet A* 2009, **149A**(6):1159-1168. doi:10.1002/ajmg.a.32800.
- Wusthoff CJ, Olson DM: **Genetic testing in children with epilepsy**. *Continuum (Minneap Minn)* 2013, **19**(3 Epilepsy):795-800. doi:10.1212/01.CON.0000431393.39099.89.
- Yen RJ: **Tourette's syndrome: a case example for mandatory genetic regulation of behavioral disorders**. *Law Psychol Rev* 2003, **27**:29-54.

Books:

- Browner CH, Preloran HM: *Neurogenetic Diagnoses: The Power of Hope and the Limits of Today’s Medicine*. London: Routledge; 2009.
- Evers-Kiebooms G, Zoeteweij MW, Harper PS: *Prenatal Testing for Late-onset Neurogenetic Diseases*. Oxford: BIOS Scientific; 2002.
- Lynch DR: *Neurogenetics: Scientific and Clinical Advances*. New York: Taylor & Francis; 2006.

Book chapters:

- Bernat JL: **Neurogenetic diseases**. In his *Ethical Issues in Neurology.* Boston: Butterworth-Heinemann; 2002:409-437.
- Blank RH: **The human brain: an introduction: the brain and genetics**. In his *Intervention in the Brain: Politics, Policy and Ethics*. Cambridge, Mass.: MIT Press; 2013:16-21.
- Caplan AL, Farah MJ: **Emerging ethical issues in neurology, psychiatry, and the neurosciences**. In *The Molecular and Genetic Basis of Neurologic and Psychiatric Disease* (3rd ed.). Edited by Roger N. Rosenberg, Stanley B. Prusiner, Salvatore DiMauro, Robert L. Barchi, Eric J. Nestler. Philadelphia, PA: Butterworth-Heinemann; 2003:109-114.
- Evers-Kiehooms G: **Comments from the praxis of predictive testing, prenatal testing, and preimplantation genetic diagnosis for late-onset neurogenetic disease: the example of Huntington's disease**. In *Genetics, Theology and Ethics: An Interdisciplinary Conversation*. Edited by Lisa Sowle Cahill. New York: Crossroad Publication Company; 2005:171-180.
- Fairhurst M: **Neurogenetic imaging**. In *Gene Therapy: Prospective Technology Assessment in its Societal Context*. Edited by Jörg Niewöhner, Christof Tannert. Amsterdam: Elsevier Publishing; 2006:77-87.
- FitzGerald K, Wurzman R: **Neurogenetics and ethics: how scientific frameworks can better inform ethics**. In *Scientific and Philosophical Perspectives in Neuroethics.* Edited by James J. Giordano, Bert Gordijn. Cambridge: Cambridge University Press; 2010: 216-229.
- Green RM: **From genome to brainome: charting the lessons learned**. In *Neuroethics: Defining the Issues in Theory, Practice, and Policy*. Edited by Judy Illes. Oxford: Oxford University Press; 2006:105-121.
- Giordano JJ: **Neurogenetic and neural tissue-implantation technology: neuroethical, legal, and social issues**. In his *Neurotechnology: Premises, Potential, and Problems*. Boca Raton: CRC Press; 2012:59-68.
- Hsiung GYR: **Ethical concerns and pitfalls in neurogenetic testing**. In *Oxford Handbook of Neuroethics*. Edited by Judy Illes, Barbara J. Sahakian. Oxford: Oxford University Press; 2011:529-552.
- Stein, D: **Philosophy and cognitive-affective neurogenetics**. In *Philosophy as Cognitive Neuroscience: Philosophical Perspectives.* Edited by Matthew R. Broome, Lisa Bortolotti. Oxford: Oxford University Press; 2009:193-201.
- Uhlmann WR: **Ethical dilemmas in neurogenetics**. In *Neurogenetics: Scientific and Clinical Advances*. Edited by David R. Lynch. New York: Taylor & Francis; 2006: 87-103.
- Zarzeczny A, Caulfield T: **Public representations of neurogenetics**. In *The Oxford Handbook of Neuroethics*. Edited by Judy Illes, Barbara J. Sahakian. Oxford: Oxford University Press; 2011:715-728.

***Neurobiomarkers:***

- Arias JJ, Karlawish J: **Confidentiality in preclinical Alzheimer disease studies: when research and medical records meet**. *Neurology* 2014, **82**(8):725-729. doi:10.1212/WNL.0000000000000153.
- Choudhury S, Gold I, Kirmayer LJ: **From brain image to the Bush doctrine: critical neuroscience and the political uses of neurotechnology**. *AJOB Neurosci* 2010, **1**(2):17-19. doi: 10.1080/21507741003699280.
- Dani KA, McCormick MT, Muir KW: **Brain lesion volume and capacity for consent in stroke trials: Potential regulatory barriers to the use of surrogate markers**. *Stroke* 2008, **39**(8):2336-2340. doi: 10.1161/STROKEAHA.107.507111.
- Davis JK: **Justice, insurance, and biomarkers of aging**. *Exp Gerontol* 2010, **45**(10):814-818. doi: 10.1016/j.exger.2010.02.004.
- Davis KD, Racine E, Collett B: **Neuroethical issues related to the use of brain imaging: can we and should we use brain imaging as a biomarker to diagnose chronic pain?***Pain* 2012, **153**(8):1555-1559. doi:10.1016/j.pain.2012.02.037.
- Dresser R: **Pre-emptive suicide, precedent autonomy and preclinical Alzheimer disease**. *J Med Ethics* 2014, **40**(8):550-551. doi: 10.1136/medethics-2013-101615.
- Farah MJ, Gillihan SJ: **The puzzle of neuroimaging and psychiatric diagnosis: technology and nosology in an evolving discipline**. *AJOB Neurosci* 2012, **3**(4):31-41. doi:10.1080/21507740.2012.713072.
- Gauthier S, Leuzy A, Racine E, Rosa-Neto P: **Diagnosis and management of Alzheimer's disease: past, present and future ethical issues**. *Prog Neurobiol* 2013, **110**:102-113. doi:10.1016/j.pneurobio.2013.01.003.
- Giordano J, Abramson K, Boswell MV: **Pain assessment: subjectivity, objectivity, and the use of neurotechnology**. *Pain Physician* 2010, **13**(4):305-315.
- Goswami U: **Principles of learning, implications for teaching: a cognitive neuroscience perspective**. *J Philos Educ* 2008, **42**(3-4):381-399. doi: 10.1111/j.1467-9752.2008.00639.x.
- Illes J, Rosen A, Greicius M, Racine E: **Prospects for prediction: ethics analysis of neuroimaging in Alzheimer's disease**. *Ann NY Acad Sci* 2007, **1097**:278-295. doi: 10.1196/annals.1379.030.
- Jones R: **Biomarkers: casting the net wide**. *Nature* 2010, **466**(7310):S11-S12. doi: 10.1038/466S11a.
- Karlawish J: **Addressing the ethical, policy, and social challenges of preclinical Alzheimer disease**. *Neurology* 2011, **77**(15):1487-1493. doi: 10.1212/WNL.0b013e318232ac1a.
- Klein E, Karlawish J: **Ethical issues in the neurology of aging and cognitive decline**. *Handb Clin Neurol* 2013, **118**:233-242. doi: 10.1016/B978-0-444-53501-6.00020-2.
- Lakhan SE, Vieira KF, Hamlat E: **Biomarkers in psychiatry: drawbacks and potential for misuse**. *Int Arch Med* 2010, **3**:1. doi: 10.1186/1755-7682-3-1.
- Lehrner A, Yehuda R: **Biomarkers of PTSD: military applications and considerations**. *Eur J Psychotraumatol* 2014, **5**. doi: 10.3402/ejpt.v5.23797.
- Mattsson N, Brax D, Zetterberg H: **To know or not to know: ethical issues related to early diagnosis of Alzheimer's disease**. *Int J Alzheimers Dis* 2010, 2010:841941. doi: 10.4061/2010/841941.
- Peters KR, Lynn Beattie B, Feldman HH, Illes J: **A conceptual framework and ethics analysis for prevention trials of Alzheimer disease**. *Prog Neurobiol* 2013, **110**:114-123. doi: 10.1016/j.pneurobio.2012.12.001.
- Petzold A et al.: **Biomarker time out**. *Mult Scler* 2014, **20**(12):1560-63. doi: 10.1177/1352458514524999.
- Pierce R: **Complex calculations: ethical issues in involving at-risk healthy individuals in dementia research**. *J Med Ethics* 2010, **36**(9):553-557. doi: 10.1136/jme.2010.036335.
- Porteri C, Frisoni GB: **Biomarker-based diagnosis of mild cognitive impairment due to Alzheimer's disease: how and what to tell: a kickstart to an ethical discussion**. *Front Aging Neurosci* 2014, **6**:41. doi:10.3389/fnagi.2014.00041.
- Prvulovic D, Hampel H: **Ethical considerations of biomarker use in neurodegenerative diseases—a case study of Alzheimer's disease**. *Prog Neurobiol* 2011, **95**(4):517-519. doi: 10.1016/j.pneurobio.2011.11.009.
- Schicktanz S, et al.: **Before it is too late: professional responsibilities in late-onset Alzheimer's research and pre-symptomatic prediction**. *Front Hum Neurosci* 2014, **8**:921. doi:10.3389/fnhum.2014.00921.
- Singh I, Rose N: **Biomarkers in psychiatry**. *Nature* 2009, **460**(7252):202-207. doi: 10.1038/460202a.
- Tarquini D, et al. **[Diagnosing Alzheimer's disease: from research to clinical practice and ethics]**. *Recenti Prog Med* 2014, **105**(7-8):295-299. doi: 10.1701/1574.17116.
- Walsh P, Elsabbagh M, Bolton P, Singh I: **In search of biomarkers for autism: scientific, social and ethical challenges**. *Nat Rev Neurosci* 2011, **12**(10):603-612. doi:10.1038/nrn3113.
- Zizzo N, et al.: **Comments and reflections on ethics in screening for biomarkers of prenatal alcohol exposure**. *Alcohol Clin Exp Res* 2013, **37**(9):1451-1455. doi: 10.1111/acer.12115.

Books:

- Lerner AC, Lerner AW: *Alzheimer's Disease*. Detroit: Greenhaven Press; 2009.
- Nuffield Council on Bioethics. *Dementia: Ethical Issues*. London: Nuffield Council on Bioethics; 2009.
- Galimberti D, Scarpini E. *Biomarkers for Early Diagnosis of Alzheimer's Disease*. New York: Nova Biomedical Books; 2008.
- Singh I, Sinnott-Armstrong WP, Savulescu J: *Bioprediction, Biomarkers, and Bad Behavior: Scientific, Legal, and Ethical Challenges*. New York: Oxford University Press; 2014.
- Takeda M, Toshihisa T, Cacabelos R: *Molecular Neurobiology of Alzheimer Disease and Related Disorders*. Basel: Karger; 2004.

Book Chapters:

- Albert MS, McKhann GM: **Neuroethical issues in early detection of Alzheimer's disease**. In *The Oxford Handbook of Neuroethics.* Edited by Judy Illes, Barbara J. Sahakian. Oxford: Oxford University Press; 2011:553-562.
- Burger K, Hampel H: **Biomarkers for the dementias**. In *American Psychiatric Publishing Textbook of Alzheimer Disease and Other Dementias.* Edited by Myron F. Weiner, Anne M. Lipton. Washington, DC: American Psychiatric Publishing; 2009:407-421.
- Dunnett SB, Björklund, A: **Stroke: clinical trials: potential biomarkers**. In their *Functional Neural Transplantation III: Primary and Stem Cell Therapies for Brain Repair.* Amsterdam: Elsevier; 2012:155-156.
- Hall WD, Gartner CE, Mathews R, Munafo M: **Technical, ethical and social issues in the bioprediction of addiction liability and treatment response**. In *Addiction Neuroethics: The Ethics of Addiction Neuroscience Research and Treatment*. Edited by Adrian Carter, Wayne Hall, Judy Illes. London: Academic Press; 2012:116-138.
- Harris JR, Gruenewald TL, Seeman T: **An overview of biomarker research from community and population-based studies on aging**. In *Biosocial Surveys*. Edited by Maxine Weinstein, James W. Vaupel, Kenneth W. Wachter, National Research Council (U.S.) Committee on Advances in Collecting and Utilizing Biological Indicators and Genetic Information in Social Science Surveys, National Research Council (U.S.) Committee on Population. Washington, D.C.: National Academies Press; 2008:96-135.
- Haynes, J. (2014). **The neural code for intentions in the human brain**. In *Bioprediction, Biomarkers, and Bad Behavior: Scientific, Legal, and Ethical Challenges*. Edited by Ilian Singh, Walter Sinnott-Armstrong, Julian Savulescu. Oxford: Oxford University Press; 2014:173-187.
- Lakhan SE, Vieira KF: **The ethical ramifications of biomarker use for mood disorders**. In *Handbook of Schizophrenia Spectrum Disorders* (Volume III). Edited by Michael S. Ritsner. Dordrecht: Springer Netherlands; 2011:421-437.
- Lock M: **The future is now: locating biomarkers for dementia**. In *Biomedicine as Culture: Instrumental Practices, Technoscientific Knowledge, and New Modes of Life*. Edited by Regula Valérie Burri, Joseph Dumit. New York: Routledge; 2007:61-85.
- Rutter M: **Biomarkers: potential and challenges**. In *Bioprediction, Biomarkers, and Bad Behavior: Scientific, Legal, and Ethical Challenges*. Oxford University Press. Edited by Ilian Singh, Walter Sinnott-Armstrong, Julian Savulescu. Oxford: Oxford University Press; 2014:188-205.
- Silva G, Furie K: **Biomarkers in neurology**. In *Clinical Trials in the Neurosciences*. Edited by K.M. Woodbury-Harris, B.M. Coull. Basel; New York: Karger; 2009:55-61.
- Wolpe PR: **Rethinking the implications of discovering biomarkers for biologically based criminality**. In *Bioprediction, Biomarkers, and Bad Behavior: Scientific, Legal, and Ethical Challenges*. Edited by Ilina Singh, Walter P. Sinnott-Armstrong, Julian Savulescu. Oxford: Oxford University Press; 2013:118–130.

***Neuropsychopharmacology:***

- Anderson IM: **Drug information not regulation is needed**. *J Psychopharmacol* 2004, **18**(1): 7-13. doi: 10.1177/0269881104040205.
- Anderson KS, Bjorklund P: **Demystifying federal nursing home regulations to improve the effectiveness of psychopharmacological care**. *Perspect Psychiatr Care* 2010, **46**(2): 152-162. doi:10.1111/j.1744-6163.2010.00251.x.
- Appelbaum PS: **Psychopharmacology and the power of narrative**. *Am J Bioeth* 2005, **5**(3): 48-49. doi: 10.1080/15265160591002773.
- Arun M, Jagadish Rao PP, Menezes RG: **The present legal perspective of narcoanalysis: winds of change in India**. *Med Leg J* 2010, **78**(Pt 4): 138-141. doi:10.1258/mlj.2010.010024.
- Bailey JE: **The application of good clinical practice to challenge tests**. *J Psychopharmacol* 2004, **18**(1): 16. doi:10.1177/0269881104040210.
- Belitz J, Bailey RA: **Clinical ethics for the treatment of children and adolescents: a guide for general psychiatrists**. *Psychiatr Clin North Am* 2009, **32**(2): 243-257. doi:10.1016/j.psc.2009.02.001.
- Benedetti F, Carlino E, Pollo A: **How placebos change the patient's brain**. *Neuropsychopharmacology* 2011, **36** (1): 339-354. doi: 10.1038/npp.2010.81.
- Bjorklund P: **Can there be a 'cosmetic' psychopharmacology? Prozac unplugged: the search for an ontologically distinct cosmetic psychopharmacology**. *Nurs Philos* 2005, **6**(2): 131-143. doi: 10.1111/j.1466-769X.2005.00213.x.
- Breithaupt H, Weigmann K: **Manipulating your mind**. *EMBO Rep* 2004, **5**(3), 230-232. doi:10.1038/sj.embor.7400109.
- Browning D: **Internists of the mind or physicians of the soul: does psychiatry need a public philosophy**? *Aust N Z J Psychiatry* 2003, **37**(2): 131-137. doi: 10.1046/j.1440-1614.2003.01135.x.
- Caplan A: **Accepting a helping hand can be the right thing to do**. *J Med Ethics* 2013, **39**(6), 367-368. doi:10.1136/medethics-2012-100879.
- Cerullo MA: **Cosmetic psychopharmacology and the President's Council on Bioethics**. *Perspect Biol Med* 2006, **49**(4): 515-523. doi: 10.1353/pbm.2006.0052.
- Cheshire WP: **Accelerated thought in the fast lane**. *Ethics Med* 2009, **25**(2): 75-78.
- Cohan JA: **Psychiatric ethics and emerging issues of psychopharmacology in the treatment of depression**. *J Contemp Health Law Policy* 2003, **20**(1):115-172.
- Conti NA, Matusevich D: **Problematic of gender in psychiatry. [Problematicas de genero en psiquiatria]***Vertex* 2008, **19**(81): 269-270.
- Czerniak E, Davidson M: **Placebo, a historical perspective**. *Eur Neuropsychopharmacol* 2012, **22**(11): 770-774. doi:10.1016/j.euroneuro.2012.04.003.
- Dell ML: **Child and adolescent depression: psychotherapeutic, ethical, and related nonpharmacologic considerations for general psychiatrists and others who prescribe**. *Psychiatr Clin North Am* 2012, **35**(1):181-201. doi:10.1016/j.psc.2011.12.002.
- Dell ML, Vaughan BS, Kratochvil CJ: **Ethics and the prescription pad**. *Child Adoles Psychiatr Clin N Am* 2008, **17**(1): 93-111, ix. doi: 10.1016/j.chc.2007.08.003.
- Derivan AT, et al.: **The ethical use of placebo in clinical trials involving children**. *J Child Adolesc Psychopharmacol* 2004, **14**(2): 169-174. doi:10.1089/1044546041649057.
- DeVeaugh-Geiss J, et al.: **Child and adolescent psychopharmacology in the new millennium: a workshop for academia, industry, and government**. *J Am Acad Child Adolesc Psychiatry* 2006, **45**(3): 261-270. doi:10.1097/01.chi.0000194568.70912.ee.
- Earp BD, Wudarczyk OA, Sandberg A, Savulescu J: **If I could just stop loving you: anti-love biotechnology and the ethics of a chemical breakup**. *Am J Bioeth* 2013, **13**(11): 3-17. doi: 10.1080/15265161.2013.839752.
- Echarte Alonso LE: **Therapeutic and cosmetic psychopharmacology: risks and limits [Psicofarmacologia terapeutica y cosmetic: riesgos y limites].***Cuad Bioet* 2009, **20**(69): 211-230.
- Echarte Alonso LE: **Neurocosmetics, transhumanism and eliminative materialism: toward new ways of eugenics [Neurocosmetica, transhumanismo y materialismo eliminativo: hacia nuevas formas de eugenesia].***Cuad Bioet* 2012, **23**(77): 37-51.
- Eisenberg L: **Psychiatry and human rights: welfare of the patient is in first place: acceptance speech for the Juan Jose Lopez Award. [Psychiatrie und menschenrechte: das wohl des patienten an erster stele: dankesrede fur die Juan Jose Lopez Ibor Auszeichnung]**. *Psychiatr Danub* 2009, **21**(3): 266-275.
- Evers K: **Personalized medicine in psychiatry: ethical challenges and opportunities**. *Dialogues Clin Neurosci* 2009, **11**(4): 427-434.
- Fava GA: **Conflict of interest in psychopharmacology: can Dr. Jekyll still control Mr. Hyde?***Psychother Psychosom* 2004, **73**(1):1-4. doi:10.1159/000074433.
- Fava GA: **The intellectual crisis of psychiatric research**. *Psychother Psychosom* 2006, **75**(4): 202-208. doi: 10.1159/000092890.
- Fava GA: **The decline of pharmaceutical psychiatry and the increasing role of psychological medicine**. *Psychother Psychosom* 2009, **78**(4): 220-227. doi:10.1159/000214443.
- Frank E, Novick DM, Kupfer DJ: **Beyond the question of placebo controls: ethical issues in psychopharmacological drug studies**. *Psychopharmacology (Berl)* 2003, **171**(1): 19-26. doi:10.1007/s00213-003-1477-z.
- Frecska E: **Neither with you, nor with you: preferably with you [Se veluk, se nelkuluk: megis inkabb veluk]**. *Neuropsychopharmacol Hung* 2004, **6**(2): 61-62.
- Gelenberg AJ, Freeman MP: **The art (and blood sport) of psychopharmacology research: who has a dog in the fight?***J Clin Psychiatry* 2007, **68**(2):185. doi: 10.4088/JCP.v68n0201.
- Geppert C, Bogenschutz MP: **Pharmacological research on addictions: a framework for ethical and policy considerations**. *J Psychoactive Drugs* 2009, **41**(1): 49-60. doi: 10.1080/02791072.2009.10400674.
- Ghaemi SN: **Toward a Hippocratic psychopharmacology**. *Can J Psychiatry* 2008, **53**(3):189-196.
- Ghaemi SN, Goodwin FK: **The ethics of clinical innovation in psychopharmacology: challenging traditional bioethics**. *Philos Ethics Humanit Med* 2007, **2**:26. doi: 10.1186/1747-5341-2-26.
- Glannon W: **Psychopharmacology and memory**. *J Med Ethics* 2006, **32**(2):74-78. doi: 10.1136/jme.2005.012575.
- Glass KC: **Rebuttal to Dr Streiner: can the "evil" in the "lesser of 2 evils" be justified in placebo-controlled trials?***Can J Psychiatry* 2008, **53**(7): 433.
- Gordijn B, Dekkers W: **Technology and the self**. *Med Health Care Philos* 2007, **10**(2):113-114. doi:10.1007/s11019-006-9046-y.
- Greely HT: **Knowing sin: making sure good science doesn't go bad**. *Cerebrum* 2006, 1-8.
- Griffith JL: **Neuroscience and humanistic psychiatry: a residency curriculum**. *Acad Psychiatry* 2014, **38**(2):177-184. doi:10.1007/s40596-014-0063-5.
- Gutheil TG: **Reflections on ethical issues in psychopharmacology: an American perspective**. *Int J Law Psychiatry* 2012, **35**(5-6): 387-391. doi:10.1016/j.ijlp.2012.09.007.
- Haroun AM: **Ethical discussion of informed consent**. *J Clin Psychopharmacol* 2005, **25**(5): 405-406.
- Jakovljević M: **The side effects of psychopharmacotherapy: conceptual, explanatory, ethical and moral issues - creative psychopharmacology instead of toxic psychiatry**. *Psychiatr Danub* 2009, **21**(1): 86-90.
- Jesani A: **Willing participants and tolerant profession: medical ethics and human rights in narco-analysis**. *Indian J Med Ethics* 2008, **5**(3):130-135.
- Kirmayer LJ, Raikhel E: **From Amrita to substance D: psychopharmacology, political economy, and technologies of the self**. *Transcult Psychiatry* 2009, **46**(1): 5-15. doi:10.1177/1363461509102284.
- Klein DF, et al.: **Improving clinical trials: American Society of Clinical Psychopharmacology recommendations**. *Arch Gen Psychiatry* 2002, **59**(3): 272-278. doi:10.1001/archpsyc.59.3.272.
- Koelch M, Schnoor K, Fegert JM: **Ethical issues in psychopharmacology of children and adolescents**. *Curr Opin Psychiatry* 2008, **21**(6): 598-605. doi:10.1097/YCO.0b013e328314b776.
- Kolch M, et al.: **Safeguarding children's rights in psychopharmacological research: ethical and legal issues**. *Curr Pharm Des* 2010, **16**(22): 2398-2406. doi: 10.2174/138161210791959881.
- Koski G: **Imagination and attention: protecting participants in psychopharmacological research**. *Psychopharmacology (Berl)* 2003, **171**(1): 56-57. doi:10.1007/s00213-003-1631-7.
- Kotzalidis G, et al.: **Ethical questions in human clinical psychopharmacology: should the focus be on placebo administration?***J Psychopharmacol* 2008, **22**(6): 590-597. doi:10.1177/0269881108089576.
- Krystal JH: **Commentary: first, do no harm: then, do some good: ethics and human experimental psychopharmacology**. *Isr J Psychiatry Relat Sci* 2002, **39**(2): 89-91.
- Langlitz N: **The persistence of the subjective in neuropsychopharmacology: observations of contemporary hallucinogen research**. *Hist Human Sci* 2010, **23**(1): 37-57. doi: 10.1177/0952695109352413.
- Levy N, Clarke S: **Neuroethics and psychiatry**. *Curr Opin Psychiatry* 2008, **21**(6): 568-571. doi:10.1097/YCO.0b013e3283126769.
- Lombard J: **Synchronic consciousness from a neurological point of view: the philosophical foundations for neuroethics**. *Synthese* 2008, **162**(3): 439-450. doi: 10.1007/s11229-007-9246-x.
- Malhotra S, Subodh BN: **Informed consent & ethical issues in paediatric psychopharmacology**. *Indian J Med Res* 2009, **129**(1): 19-32.
- McHenry L: **Ethical issues in psychopharmacology**. *J Med Ethics* 2006, **32**(7): 405-410. doi: 10.1136/jme.2005.013185.
- Miskimen T, Marin H, Escobar J: **Psychopharmacological research ethics: special issues affecting US ethnic minorities**. *Psychopharmacology (Berl)* 2003, **171**(1): 98-104. doi:10.1007/s00213-003-1630-8.
- Mohamed AD, Sahakian BJ: **The ethics of elective psychopharmacology**. *Int J Neuropsychopharmacol* 2012, **15**(4): 559-571. doi:10.1017/S146114571100037X.
- Mohamed AD: **Reducing creativity with psychostimulants may debilitate mental health and well-being**. *JMH* 2014, **9**(1): 146-163. doi:10.1080/15401383.2013.875865.
- Morris GH, Naimark D, Haroun AM: **Informed consent in psychopharmacology**. *J Clin Psychopharmacol* 2005, **25**(5): 403-406. doi: 10.1097/01.jcp.0000181028.12439.81.
- Nierenberg AA, et al.: **Critical thinking about adverse drug effects: lessons from the psychology of risk and medical decision-making for clinical psychopharmacology**. *Psychother Psychosom* 2008, **77**(4): 201-208. doi:10.1159/000126071.
- Novella EJ: **Mental health care in the aftermath of deinstitutionalization: a retrospective and prospective view**. *Health Care Anal* 2010, **18**(3): 222-238. doi:10.1007/s10728-009-0138-8.
- Perlis RH, et al.: **Industry sponsorship and financial conflict of interest in the reporting of clinical trials in psychiatry**. *Am J Psychiatry* 2005, **162**(10): 1957-1960. doi: 10.1176/appi.ajp.162.10.1957.
- Puzyński S: **Placebo in the investigation of psychotropic drugs, especially antidepressants**. *Sci Eng Ethics* 2004, **10**(1): 135-142. doi: 10.1007/s11948-004-0070-0.
- Rihmer, Z., Dome, P., Baldwin, D. S., & Gonda, X. (2012). **Psychiatry should not become hostage to placebo: an alternative interpretation of antidepressant-placebo differences in the treatment response in depression**. *Eur Neuropsychopharmacol* 2012, **22**(11): 782-786. doi:10.1016/j.euroneuro.2012.03.002.
- Roberts LW, Krystal J: **A time of promise, a time of promises: ethical issues in advancing psychopharmacological research**. *Psychopharmacology (Berl)* 2003, **171**(1): 1-5. doi:10.1007/s00213-003-1704-7.
- Roberts LW, et al.: **Schizophrenia patients' and psychiatrists' perspectives on ethical aspects of symptom re-emergence during psychopharmacological research participation**. *Psychopharmacology (Berl)* 2003, **171**(1): 58-67. doi:10.1007/s00213-002-1160-9.
- Rosenstein DL, Miller FG: **Ethical considerations in psychopharmacological research involving decisionally impaired subjects**. *Psychopharmacology (Berl)* 2003, **171**(1): 92-97. doi:10.1007/s00213-003-1503-1.
- Rudnick A: **The molecular turn in psychiatry: a philosophical analysis**. *The J Med Philos* 2002, **27**(3): 287-296. doi:10.1076/jmep.27.3.287.2979.
- Rudnick A: **Re: toward a Hippocratic psychopharmacology**. *Can J Psychiatry* 2009, **54**(6): 426.
- Safer DJ: **Design and reporting modifications in industry-sponsored comparative psychopharmacology trials**. *J Nerv Ment Dis* 2002, **190**(9): 583-592. doi:10.1097/01.NMD.0000030522.74800.0D.
- Schermer MH: **Brave new world versus island--utopian and dystopian views on psychopharmacology**. *Med Health Care Philos* 2007, **10**(2): 119-128. doi:10.1007/s11019-007-9059-1.
- Schmal C, et al.: **Pediatric psychopharmacological research in the post EU regulation 1901/2006 era**. *Z Kinder Jugendpsychiatr Psychother* 2014, **42**(6): 441-449. doi:10.1024/1422-4917/a000322.
- Sententia W: **Neuroethical considerations - cognitive liberty and converging technologies for improving human cognition**. *Ann N Y Acad Sci* 2004, **1013**: 221-228. doi:10.1196/annals.1305.014.
- Sergeant JA, et al.: **Eunethydis: a statement of the ethical principles governing the relationship between the European group for ADHD guidelines, and its members, with commercial for-profit organisations**. *Eur Child Adoles Psychiatry* 2010, **19**(9): 737-739. doi: 10.1007/s00787-010-0114-8.
- Singh I: **Not robots: children's perspectives on authenticity, moral agency and stimulant drug treatments**. *J Med Ethics* 2013, **39**(6): 359-366. doi: 10.1136/medethics-2011-100224. 
- Singh I: **Will the “real boy” please behave: dosing dilemmas for parents of boys with ADHD**. *Am J Bioeth* 2005, **5**(3): 34-47. doi: 10.1080/15265160590945129.
- Smith ME, Farah MJ: **Are prescription stimulants "smart pills"? the epidemiology and cognitive neuroscience of prescription stimulant use by normal health individuals**. *Psychol Bull* 2011, **137**(5): 717-741. doi: 10.1037/a0023825.
- Sobredo LD, Levin SA: **We hear about "gender psychopharmacology": are we listening well? [Se escucha hablar de psicofarmacologia de genero: estaremos escuchando bien?]***Vertex* 2008, **19**(81): 276-279.
- Stein DJ: **Cosmetic psychopharmacology of anxiety: bioethical considerations**. *Curr Psychiatry Rep* 2005, **7**(4): 237-238. doi: 10.1007/s11920-005-0072-x.
- Street LL, Luoma JB: **Control groups in psychosocial intervention research: ethical and methodological issues**. *Ethics Behav* 2002, **12**(1): 1-30. doi:10.1207/S15327019EB1201_1.
- Streiner DL: **The lesser of 2 evils: the ethics of placebo-controlled trials**. *Can J Psychiatry* 2008, **53**(7): 430-432.
- Strous RD: **Ethical considerations in clinical training, care and research in psychopharmacology**. *Int J Neuropsychopharmacol* 2011, **14**(3): 413-424. doi:10.1017/S1461145710001112.
- Suárez RM: **Psychiatry and neuroethics [Psiquiatría y neuroética]**. *Vertex* 2013, **24**(109): 233-240.
- Svenaeus F: **Psychopharmacology and the self: an introduction to the theme**. *Med Health Care Philos* 2007, **10**(2): 115-117. doi:10.1007/s11019-007-9057-3.
- Synofzik M: **Intervening in the neural basis of one's personality: an ethical analysis of neuropharmacology and deep-brain stimulation [Eingriffe in die grundlagen der persönlichkeit: eine praxisorientierte ethische analyse von neuropharmaka und tiefhirnstimulation]**. *Dtsch Med Wochenschr* 2007, **132**(50): 2711-2713. doi: 10.1055/s-2007-993124.
- Synofzik M: **Intervening in the neural basis of one's personality: a practice-oriented ethical analysis of neuropharmacology and deep-brain stimulation**. *Dtsch Med Wochenschr* 2007, **132**(50): 2711-2713. doi:10.1055/s-2007-993124.
- Terbeck S, Chesterman LP: **Will there ever be a drug with no or negligible side effects? evidence from neuroscience**. *Neuroethics* 2014, **7**(2): 189-194. doi: 10.1007/s12152-013-9195-7.
- Thorens G, Gex-Fabry M, Zullino SF, Eytan A: **Attitudes toward psychopharmacology among hospitalized patients from diverse ethno-cultural backgrounds**. *BMC Psychiatry* 2008, **8**:55. doi:10.1186/1471-244X-8-55.
- Touwen DP, Engberts DP: **Those famous red pills-deliberations and hesitations: ethics of placebo use in therapeutic and research settings**. *Eur Neuropsychopharmacol* 2012, **22**(11): 775-781. doi:10.1016/j.euroneuro.2012.03.005.
- Vince G: **Rewriting your past: drugs that rid people of terrifying memories could be a lifeline for many: but could they have a sinister side too?***New Sci* 2005, **188**(2528): 32-35.
- Vitiello B: **Ethical considerations in psychopharmacological research involving children and adolescents**. *Psychopharmacology(Berl)* 2003, **171**(1): 86-91. doi:10.1007/s00213-003-1400-7.
- Vrecko S: **Neuroscience, power and culture: an introduction**. *Hist Human Sci* 2010, **23**(1): 1-10. doi: 10.1177/0952695109354395.
- Weinmann S: **Meta-analyses in psychopharmacotherapy: garbage in--garbage out?[Metaanalysen zur psychopharmakotherapie: garbage in--garbage out?]**. *Psychiatri Prax* 2009, **36**(6): 255-257. doi:10.1055/s-0029-1220425 [doi]
- Wisner KL, et al.: **Researcher experiences with IRBs: a survey of members of the American College of Neuropsychopharmacology**. *IRB* 2011, **33**(5): 14-20. doi: 10.2307/23048300.
- Young SN, Annable L: **The ethics of placebo in clinical psychopharmacology: the urgent need for consistent regulation**. *J Psychiatry Neurosci* 2002, **27**(5): 319-321.
- Young SN: **Acute tryptophan depletion in humans: a review of theoretical, practical and ethical aspects**. *J Psychiatry Neurosci* 2013, **38**(5): 294-305. doi:10.1503/jpn.120209.

Books:

- Borkenhagen A et al.: *Die Selbstverbesserung des Menschen : Wunschmedizin und Enhancement aus Medizinpsychologischer Perspektive*. Giessen: Psychosozial-Verlag; 2012.

Book Chapters:

- Bailey R: **Changing your own mind: the neuroethics of psychopharmacology**. In his *Liberation Biology: The Scientific and Moral Case for the Biotech Revolution*. Amherst, N.Y.: Prometheus Books; 2005: 223-238.
- Fukuyama F: **Neuropharmacology and the control of behavior**. In his *Our Posthuman Future: Consequences of the Biotechnology Revolution*. New York: Farrar, Straus and Giroux; 2002: 41-56.
- Hyman SE: **Ethical issues in psychopharmacology: research and practice**. *Neuroethics: Mapping the Field*. Edited by Steven J. Marcus. New York: Dana Press; 2002: 135-143.
- Langlitz N: **Neuropsychopharmacology as spiritual technology**. In his *Neuropsychedelia: The Revival of Hallucinogen Research since the Decade of the Brain*. Berkeley: University of California Press; 2013: 1-23.
- Pinals DA, Appelbaum PD: **Ethical aspects of neuropsychiatric research with human subjects**. In *Neuropsychopharmacology: The Fifth Generation of Progress.* Edited by Kenneth L. Davis, Dennis Charney, Joseph T. Coyle. Philadelphia, Pennsylvania: Lippincott, Williams, & Wilkins; 2002: 475-483.

***Mood stabilizers/anti-depressants/antipsychotic and nootropic agents:***

- Adam D, Kasper S, Moller HJ, Singer EA, 3rd European Expert Forum on Ethical Evaluation of Placebo-Controlled Studies in Depression: **Placebo-controlled trials in major depression are necessary and ethically justifiable: how to improve the communication between researchers and ethical committees**. *Eur Arch Psychiatry Clin Neurosci* 2005, **255**(4):258-260. doi:10.1007/s00406-004-0555-5.
- Agius M, Bradley V, Ryan D, Zaman R: **The ethics of identifying and treating psychosis early**. *Psychiatr Danub* 2008, **20**(1):93-96.
- Allison SK: **Psychotropic medication in pregnancy: ethical aspects and clinical management**. *J Perinat Neonatal Nurs* 2004, **18**(3):194-205.
- Alpert JE et al.: Enrolling research subjects from clinical practice: ethical and procedural issues in the sequenced treatment alternatives to relieve depression (STAR*D) trial. *Psychiatry Res* 2006, **141**(2):193-200. doi:10.1016/j.psychres.2005.04.007.
- Amsterdam JD, McHenry LB: **The paroxetine 352 bipolar trial: a study in medical ghostwriting**. *Int J Risk Saf Med* 2012, **24**(4):221-231. doi: 10.3233/JRS-2012-0571.
- Anderson IM, Haddad PM: **Prescribing antidepressants for depression: time to be dimensional and inclusive**. *Br J Gen Pract* 2011, **61**(582):50-52. doi:10.3399/bjgp11X548992.
- Arcand M et al.: **Should drugs be prescribed for prevention in the case of moderate to severe dementia?***Revue Geriatr* 2007, **32**(3):189-200.
- Aydin N et al.: **A report by Turkish Association for Psychopharmacology on the psychotropic drug usage in Turkey and medical, ethical and economical consequences of current applications**. *Klinik Psikofarmakol BuÌˆlteni* 2013, **23**(4):390-402. doi:10.5455/bcp.20131230121254.
- Baertschi B: **The happiness pill…why not? [La pilule du bonheur… Pourquoi non?]***Rev Med Suisse* 2006, **2**(90):2816-2820.
- Baker CB et al.: **Quantitative analysis of sponsorship bias in economic studies of antidepressants**. *Br J Psychiatry* 2003, **183**:498-506.
- Baldwin D et al.: **Placebo-controlled studies in depression: necessary, ethical and feasible**. *Eur Arch Psychiatry Clin Neurosci* 2003, **253**(1):22-28. doi:10.1007/s00406-003-0400-2.
- Ballard C, Sorensen S, Sharp S: **Pharmacological therapy for people with Alzheimer's disease: the balance of clinical effectiveness, ethical issues and social and healthcare costs**. *J Alzheimers Dis* 2007, **12**(1):53-59.
- Bartlett P: **A matter of necessity? enforced treatment under the Mental Health Act. R. (JB) v. Responsible Medical Officer Dr A Haddock, Mental Health Act Commission second opinion appointed doctor Dr Rigby, Mental Health Act Commission second opinion appointed Doctor Wood**. *Med Law Rev* 2007, **15**(1) 86-98. doi: 10.1093/medlaw/fwl027.
- Basil B, Adetunji B, Mathews M, Budur K: **Trial of risperidone in India--concerns**. *Br J Psychiatry* 2006, **188**:489-90; doi: 10.1192/bjp.188.5.489-b.
- Berger JT, Majerovitz SD: **Do elderly persons' concerns for family burden influence their preferences for future participation in dementia research?***J Clin Ethics* 2005, **16**(2):108-115.
- Bernheim E: **Psychiatric medication as restraint: between autonomy and protection, is there place for a legal framework? [La medication psychiatrique comme contention : entre autonomie et protection, quelle place pour un cadre juridique ?]***Sante Ment Que* 2010, **35**(2):163-184. doi: 10.7202/1000558ar.
- Berns A: **Dementia and antipsychotics: a prescription for problems**. *J Leg Med* 2012, **33**(4):553-569. doi:10.1080/01947648.2012.739067.
- Biegler P: **Autonomy, stress, and treatment of depression**. *BMJ* 2008, **336**(7652):1046-1048. doi:10.1136/bmj.39541.470023.AD.
- Biegler P: **Autonomy and ethical treatment in depression**. *Bioethics* 2010, **24**(4):179-189. doi:10.1111/j.1467-8519.2008.00710.x.
- Block JJ: **Ethical concerns regarding olanzapine versus placebo in patients prodromally symptomatic for psychosis**. *Am J Psychiatry* 2006, **163**(10):1838. doi: 10.1176/ajp.2006.163.10.1838.
- Borger BA: **Sell v. United States: the appropriate standard for involuntarily administering antipsychotic drugs to dangerous detainees for trial**. *Seton Hall Law Rev* 2005, **35**(3):1099-1120.
- Broich K: **Klinische pruefungen mit antidepressiva und antipsychotika. das fuer und wider von placebokontrollen [clinical trials using antidepressants and antipsychotics. the pros and cons of placebo control]**. *Bundesgesundheitsblatt - Gesundheitsforschung – Gesundheitsschutz* 2005, **48**(5):541-547. doi: 10.1007/s00103-005-1038-1.
- Buller T, Shriver A, Farah M: **Broadening the focus**. *Camb Q Healthc Ethics* 2014, **23**(2):124-128. doi:10.1017/S0963180113000650.
- Charlton BG: **If 'atypical' neuroleptics did not exist, it wouldn't be necessary to invent them: perverse incentives in drug development, research, marketing and clinical practice**. *Med Hypotheses* 2005, **65**(6):1005-1009. doi: 10.1016/j.mehy.2005.08.013.
- Claassen D: **Financial incentives for antipsychotic depot medication: ethical issues**. *J Med Ethics* 2007, **33**(4):189-193. doi: 10.1136/jme.2006.016188.
- Cohan JA: **Psychiatric ethics and emerging issues of psychopharmacology in the treatment of depression**. *J Contemp Health Law Policy* 2003, **20**(1):115-172.
- Cohen D, Jacobs DH: **Randomized controlled trials of antidepressants: clinically and scientifically irrelevant**. *Debates in Neuroscience* 2007, **1**(1):44-54. doi: 10.1007/s11559-007-9002-x.
- Couzin-Frankel J: **A lonely crusade**. *Science* 2014, **344**(6186):793-797. doi: 10.1126/science.344.6186.793.
- Coyne J: **Lessons in conflict of interest: the construction of the martyrdom of David Healy and the dilemma of bioethics**. *Am J Bioeth* 2005, **5**(1):W3-14. doi: 10.1080/15265160590969114.
- Davis JM et al.: **Should we treat depression with drugs or psychological interventions? a reply to Ioannidis**. *Philos Ethics Humanit Med* 2011, 6:8. doi: 10.1186/1747-5341-6-8.
- DeMarco JP, Ford PJ: **Neuroethics and the ethical parity principle**. *Neuroethics* 2014, **7**(3):317-325. doi: 10.1007/s12152-014-9211-6.
- Dhiman GJ, Amber KT: Pharmaceutical ethics and physician liability in side effects. *J Med Humanit* 2013, 34(4):497-503. doi:10.1007/s10912-013-9239-3.
- Di Pietro N, Illes J, Canadian Working Group on Antipsychotic Medications and Children: **Rising antipsychotic prescriptions for children and youth: cross-sectoral solutions for a multimodal problem**. *CMAJ 2014,***186**(9):653-654. doi:10.1503/cmaj.131604.
- Ecks S, Basu S: **The unlicensed lives of antidepressants in India: generic drugs, unqualified practitioners, and floating prescriptions**. *Transcult Psychiatry* 2009, **46**(1):86-106. doi:10.1177/1363461509102289.
- Elliott C: **Against happiness**. *Med Health Care Philos* 2007, **10**(2):167-171. doi:10.1007/s11019-007-9058-2.
- Epstein AJ, Asch DA, Barry CL: **Effects of conflict-of-interest policies in psychiatry residency on antidepressant prescribing**. *LDI Issue Brief* 2013, **18**(3):1-4.
- Epstein AJ et al.: **Does exposure to conflict of interest policies in psychiatry residency affect antidepressant prescribing?***Med Care* 2013, **51**(2):199-203. doi:10.1097/MLR.0b013e318277eb19.
- Farlow MR: **Randomized clinical trial results for donepezil in Alzheimer's disease: is the treatment glass half full or half empty?***J Am Geriatr Soc* 2008, **56**(8):1566-1567. doi:10.1111/j.1532-5415.2008.01853.x.
- Fast J: **When is a mental health clinic not a mental health clinic? drug trial abuses reach social work**. *Soc Work* 2003, **48**(3):425-427. doi: 10.1093/sw/48.3.425.
- Filaković P, Degmecić D, Koić E, Benić D: **Ethics of the early intervention in the treatment of schizophrenia**. *Psychiatr Danub* 2007, **19**(3):209-215.
- Fisk JD: **Ethical considerations for the conduct of antidementia trials in Canada**. *Can J Neurol Sci* 2007, **34**( Suppl 1):S32-S36. doi: 10.1017/S0317167100005539.
- Flaskerud JH: **American culture and neuro-cognitive enhancing drugs**. *Issues Ment Health Nurs* 2010, **31**(1):62-63. doi:10.3109/01612840903075395.
- Fleischhacker WW et al.: **Placebo or active control trials of antipsychotic drugs?***Arch Gen Psychiatry* 2003, **60**(5): 458-464. doi:10.1001/archpsyc.60.5.458.
- Francey SM: **Who needs antipsychotic medication in the earliest stages of psychosis? a reconsideration of benefits, risks, neurobiology and ethics in the era of early intervention.***Schizophr Res* 2010, **119**(1-3):1-10. doi:10.1016/j.schres.2010.02.1071.
- Gardner P: **Distorted packaging: marketing depression as illness, drugs as cure**. *J Med Humanit* 2003, **24**(1-2):105-130. doi: 10.1023/A:1021314017235.
- Gauthier S, Leuzy A, Racine E, Rosa-Neto P: **Diagnosis and management of Alzheimer's disease: past, present and future ethical issues**. *Prog Neurobiol* 2013, **110**:102-113. doi: 10.1016/j.pneurobio.2013.01.003.
- Gilstad JR, Finucane TE: **Results, rhetoric, and randomized trials: the case of donepezil**. *J Am Geriatr Soc* 2008, **56**(8):1556-1562. doi:10.1111/j.1532-5415.2008.01844.x.
- Gjertsen MK, von Mehren Saeterdal I, Thürmer H: **Questionable criticism of the report on antidepressive agents. [Tvilsom kritikk av rapport om antidepressive legemidler]***Tidsskr Nor Laegeforen* 2008, **128**(4):475.
- Gold I, Olin L: **From Descartes to desipramine: psychopharmacology and the self.***Transcult Psychiatry* 2009, **46**(1):38-59. doi:10.1177/1363461509102286.
- Greely H et al.: **Towards responsible use of cognitive-enhancing drugs by the healthy.***Nature* 2008, **456**(7223):702-705. doi:10.1038/456702a.
- Gross DE: **Presumed dangerous: California's selective policy of forcibly medicating state prisoners with antipsychotic drugs**. *Univ Calif Davis Law Rev* 2002, **35**:483-517.
- Hamann J et al.: **Do patients with schizophrenia wish to be involved in decisions about their medical treatment?***Am Journal of Psychiatry,***162**(12):2382-2384. doi:10.1176/appi.ajp.162.12.2382.
- Heinrichs DW: **Antidepressants and the chaotic brain: implications for the respectful treatment of selves**. *Philos Psychiatr Psychol* 2005, **12**(3):215-227. doi: 10.1353/ppp.2006.0006.
- Hellander M: **Medication-induced mania: ethical issues and the need for more research**. *J Child Adolesc Psychopharmacol* 2003, **13**(2):199. doi:10.1089/104454603322163916.
- Herzberg D: **Prescribing in an age of "wonder drugs."***MD Advis* 2011, **4**(2):14-18.
- Hoffman GA: **Treating yourself as an object: self-objectification and the ethical dimensions of antidepressant use**. *Neuroethics* 2013, **6**(1):165-178. doi: 10.1007/s12152-012-9162-8.
- Howe EG: **Ethical challenges when patients have dementia**. *J Clin Ethics* 2011, **22**(3):203-211.
- Hudson TJ et al.: **Disparities in use of antipsychotic medications among nursing home residents in Arkansas**. *Psychiatr Serv* 2005, **56**(6):749-751. doi: 10.1176/appi.ps.56.6.749.
- Huf W et al.: **Meta-analysis: fact or fiction? how to interpret meta-analyses**. *World J Biol Psychiatry* 2011, **12**(3):188-200. doi:10.3109/15622975.2010.551544.
- Hughes JC: **Quality of life in dementia: an ethical and philosophical perspective**. *Expert Rev Pharmacoecon Outcomes Res* 2003, **3**(5):525-534. doi:10.1586/14737167.3.5.525.
- Huizing AR, Berghmans RLP, Widdershoven GAM, Verhey FRJ: **Do caregivers' experiences correspond with the concerns raised in the literature? ethical issues related to anti-dementia drugs**. *Int J Geriatr Psychiatry* 2006, **21**(9):869-875. doi: 10.1002/gps.1576.
- Ihara H, Arai H: **Ethical dilemma associated with the off-label use of antipsychotic drugs for the treatment of behavioral and psychological symptoms of dementia.***Psychogeriatrics* 2008, **8**(1):32-37. doi:10.1111/j.1479-8301.2007.00215.x.
- Iliffe S: **Thriving on challenge: NICE's dementia guidelines**. *Expert Rev Pharmacoecon Outcomes Res* 2007, **7**(6):535-538. doi: 10.1586/14737167.7.6.535.
- Ioannidis JP: **Effectiveness of antidepressants: an evidence myth constructed from a thousand randomized trials?***Philos Ethics Humanit Med* 2008, **3**:14. doi: 10.1186/1747-5341-3-14.
- Jacobs DH, Cohen D: **The make-believe world of antidepressant randomized controlled trials -- an afterword to Cohen and Jacobs**. *Journal of Mind and Behavior* 2010, **31**(1-2):23-36.
- Jakovljević M: **New generation vs. first generation antipsychotics debate: pragmatic clinical trials and practice-based evidence**. *Psychiatr Danub* 2009, **21**(4):446-452.
- Jotterand F: **Psychopathy, neurotechnologies, and neuroethics**. *Theor Med Bioeth* 2014, **35**(1):1-6. doi:10.1007/s11017-014-9280-x.
- Khan MM: **Murky waters: the pharmaceutical industry and psychiatrists in developing countries**. *Psychiatr Bull* 2006, **30**(3):85-88.
- Kim SY, Holloway RG: **Burdens and benefits of placebos in antidepressant clinical trials: a decision and cost-effectiveness analysis**. *Am J Psychiatry* 2003, **160**(7):1272-1276. doi: 10.1176/appi.ajp.160.7.1272.
- Kim SY, et al.: **Preservation of the capacity to appoint a proxy decision maker: implications for dementia research**. *Arch Gen Psychiatry* 2011, **68**(2):214-220. doi:10.1001/archgenpsychiatry.2010.191.
- Kirsch I: **The use of placebos in clinical trials and clinical practice**. *Can J Psychiatry* 2011, **56**(4):191-2.
- Klemperer D: **Drug research: marketing before evidence, sales before safety**. *Dtsch Arztebl Int* 2010, **107**(16) 277-278. doi:10.3238/arztebl.2010.0277.
- Leguay D et al.: **Evolution of the social autonomy scale (EAS) in schizophrenic patients depending on their management. [Evolution de l'autonomie sociale chez des patients schizophrenes selon les prises en charge. L'etude ESPASS]***Encephale* 2010, **36**(5):397-407. doi:10.1016/j.encep.2010.01.004.
- Leibing A: **The earlier the better: Alzheimer's prevention, early detection, and the quest for pharmacological interventions**. *Cult Med Psychiatry* 2014, **38**(2):217-236. doi:10.1007/s11013-014-9370-2.
- Lisi D: **Response to "results, rhetoric, and randomized trials: the case of donepezil"**. *J Am Geriatr Soc* 2009, **57**(7):1317-8; doi:10.1111/j.1532-5415.2009.02331.x.
- McConnell S, Karlawish J, Vellas B, DeKosky S: **Perspectives on assessing benefits and risks in clinical trials for Alzheimer's disease**. *Alzheimers Dement* 2006, **2**(3):160-163. doi:10.1016/j.jalz.2006.03.015.
- McGlashan TH: **Early detection and intervention in psychosis: an ethical paradigm shift.***Br J Psychiatry Suppl* 2005, **48**:s113-s115. doi: 10.1192/bjp.187.48.s113.
- McGoey L: **Compounding risks to patients: selective disclosure is not an option**. *Am J Bioeth* 2009, **9**(8):35-36. doi:10.1080/15265160902979798.
- McGoey L, Jackson E: **Seroxat and the suppression of clinical trial data: regulatory failure and the uses of legal ambiguity**. *J Med Ethics* 2009, **35**(2):107-112. doi:10.1136/jme.2008.025361.
- McGoey L: **Profitable failure: antidepressant drugs and the triumph of flawed experiments**. *Hist Human Sci* 2010, **23**(1):58-78. doi: 10.1177/0952695109352414.
- McHenry L: **Ethical issues in psychopharmacology**. *J Med Ethics* 2006, **32**(7):405-410. doi: 10.1136/jme.2005.013185.
- Meesters Y, Ruiter MJ, Nolen WA: **Is it acceptable to use placebos in depression research? [Is het gebruik van placebo in onderzoek bij depressie aanvaardbaar?]***Tijdschr Psychiatr* 2010, **52**(8):575-582.
- Millán-González R: **Consentimientos informados y aprobación por parte de los comités de ética en los estudios de antipsicóticos atípicos para el manejo del delírium [informed consent and the approval by ethics committees of studies involving the use of atypical antipsychotics in the management of delirium]**. *Rev Colomb Psiquiatr* 2012, **41**(1):150-164. doi: 10.1016/S0034-7450(14)60074-3.
- Moller HJ: **Are placebo-controlled studies required in order to prove efficacy of antidepressants?***World J BiolPsychiatry* 2005, **6**(3):130-131. doi: 10.1080/15622970510030108.
- Moncrieff J, Double D: **Double blind random bluff**. *Ment Health Today* 2003:24-26.
- Morse SJ: **Involuntary competence**. *Behav Sci Law* 2003, **21**(3):311-328. doi:10.1002/bsl.538.
- Moskowitz DS: **Quarrelsomeness in daily life**. *J Pers* 2010, **78**(1):39-66. doi:10.1111/j.1467-6494.2009.00608.x.
- Moynihan R: **Evening the score on sex drugs: feminist movement or marketing masquerade?***BMJ* 2014, **349**:g6246. doi:10.1136/bmj.g6246.
- Muller S: **Body integrity identity disorder (BIID)--is the amputation of healthy limbs ethically justified?***Am J Bioeth* 2009, **9**(1):36-43. doi:10.1080/15265160802588194.
- Müller S, Walter H: **Reviewing autonomy: implications of the neurosciences and the free will debate for the principle of respect for the patient's autonomy**. *Camb Q Healthc Ethics* 2010, **19**(2):205-217. doi:10.1017/S0963180109990478.
- Murtagh A, Murphy KC: **Trial of risperidone in India--concerns**. *Br J Psychiatry* 2006, **188**:489. doi: 10.1192/bjp.188.5.489-a.
- Naarding P, van Grevenstein M, Beekman AT: **Benefit-risk analysis for the clinician: 'primum non nocere' revisited--the case for antipsychotics in the treatment of behavioural disturbances in dementia**. *Int J Geriatr Psychiatry* 2010, **25**(5):437-440. doi:10.1002/gps.2357.
- Newton J, Langlands A: **Depressing misrepresentation?***Lancet* 2004, **363**(9422):1732. doi:10.1016/S0140-6736(04)16262-4.
- Olsen JM: **Depression, SSRIs, and the supposed obligation to suffer mentally**. *Kennedy Inst Ethics J* 2006, **16**(3):283-303. doi: 10.1353/ken.2006.0019.
- Orfei MD, Caltagirone C, Spalletta G: **Ethical perspectives on relations between industry and neuropsychiatric medicine**. *Int Rev Psychiatry* 2010, **22**(3):281-287. doi:10.3109/09540261.2010.484014.
- Patel V: **Ethics of placebo-controlled trial in severe mania**. *Indian J Med Ethics* 2006, **3**(1):11-12.
- Perman E: **Physicians report verbal drug information: cases scrutinized by the IGM [Lakare anmaler muntlig lakemedelsinformation. Arenden behandlade av IGM]***Lakartidningen* 2004, **101**(35):2648, 2650.
- Pitkälä K: **When should the medication for dementia be stopped? [Milloin dementialaakityksen voi lopettaa?]***Duodecim* 2003, **119**(9):817-818.
- Poses RM: **Efficacy of antidepressants and USPSTF guidelines for depression screening.***Ann Intern Med* 2010, **152**(11):753. doi:10.7326/0003-4819-152-11-201006010-00016.
- Preda A: **Shared decision making in schizophrenia treatment**. *J Clin Psychiatry* 2008, **69**(2):326.
- Quinlan M: **Forcible medication and personal autonomy: the case of Charles Thomas Sell**. *Spec Law Dig Health Care Law* 2005, **311**:9-33.
- Ragan M, Kane CF: **Meaningful lives: elders in treatment for depression**. *Arch Psychiatr Nurs* 2010, **24**(6):408-417. doi:10.1016/j.apnu.2010.04.002.
- Rajna P: **Living with lost individuality: special concerns in medical care of severely demented Alzheimer patients. [Elni az egyeniseg elvesztese utan. A sulyos Alzheimer-betegek orvosi ellatasanak sajatos szempontjai]***Ideggyogy Sz* 2010, **63**(11-12):364-376.
- Rasmussen-Torvik LJ, McAlpine DD: **Genetic screening for SSRI drug response among those with major depression: great promise and unseen perils**. *Depress Anxiety* 2007, **24**(5):350-357. doi:10.1002/da.20251.
- Raven M, Stuart GW, Jureidini J: **‘Prodromal’ diagnosis of psychosis: ethical problems in research and clinical practice**. *Aust N Z J Psychiatry* 2012, **46**(1):64-65. doi:10.1177/0004867411428917.
- Raz A et al.: **Placebos in clinical practice: comparing attitudes, beliefs, and patterns of use between academic psychiatrists and nonpsychiatrists**. *Can J Psychiatry* 2011, **56**(4):198-208.
- Reichlin M: **The challenges of neuroethics**. *Funct Neurol* 2007, **22**(4):235-242.
- Roberts LW, Geppert CM: **Ethical use of long-acting medications in the treatment of severe and persistent mental illnesses**. *Compr Psychiatry* 2004, **45**(3):161-167. doi:10.1016/j.comppsych.2004.02.003.
- Roehr B: **Professor files complaint of scientific misconduct over allegation of ghostwriting**. *BMJ* 2011, **343**:d4458. doi:10.1136/bmj.d4458.
- Roehr B: **Marketing of antipsychotic drugs targeted doctors of Medicaid patients, report says**. *BMJ* 2012, **345**:e6633. doi:10.1136/bmj.e6633.
- Rose S: **How smart are smart drugs?***Lancet* 2008, **372**(9634):198-199. doi:10.1016/S0140-6736(08)61058-2.
- Rose SP: **‘Smart drugs’: do they work? are they ethical? will they be legal?***Nat Rev Neurosci* 2002, **3**(12):975-979. doi:10.1038/nrn984.
- Rudnick A: **Re: toward a Hippocratic psychopharmacology**. *Can J Psychiatry* 2009, **54**(6):426.
- Schneider CE: **Benumbed**. *Hastings Cent Rep* 2004, **34**(1):9-10.
- Shivakumar G, Inrig S, Sadler JZ: **Community, constituency, and morbidity: applying Chervenak and McCullough's criteria**. *Am J Bioeth* 2011, **11**(5):57-60. doi:10.1080/15265161.2011.578466.
- Silverman BC, Gross AF: **Weighing risks and benefits of prescribing antidepressants during pregnancy**. *Virtual Mentor* 2013, **15**(9):746-752. doi:10.1001/virtualmentor.2013.15.9.ecas1-1309.
- Singer EA: **The necessity and the value of placebo**. *Sci Eng Ethics* 2004, **10**(1):51-56. doi: 10.1007/s11948-004-0062-0.
- Snyder M, Platt L: **Substance use and brain reward mechanisms in older adults**. *J Psychosoc Nurs Ment Health Serv* 2013, **51**(7):15-20. doi:10.3928/02793695-20130530-01.
- Soderfeldt Y, Gross D: **Information, consent and treatment of patients with Morgellons disease: an ethical perspective**. *Am J Clin Dermatol* 2014, **15**(2):71-76. doi:10.1007/s40257-014-0071-y.
- Srinivasan S et al.: **Trial of risperidone in India--concerns**. *Br J Psychiatry* 2006, **188**:489. doi:10.1192/bjp.188.5.489.
- Steinert T: **CUtLASS 1 - increasing disillusion about 2nd generation neuroleptics. [CUtLASS 1 - zunehmende ernuchterung bezuglich neuroleptika der 2. generation]***Psychiatr Prax* 2007, **34**(5):255-257. doi:10.1055/s-2007-984998.
- Steinert, T: **Ethical attitudes towards involuntary admission and involuntary treatment of patients with schizophrenia. [ethische einstellungen zu zwangsunterbringung und -behandlung schizophrener patienten]***Psychiatr Prax* 2007, **34** (Suppl 2),S186-S190. doi:10.1055/s-2006-952003.
- Steinert T, Kallert TW: **Involuntary medication in psychiatry. [medikamentose zwangsbehandlung in der psychiatrie]**. *Psychiatr Prax* 2006, **33**(4):160-169. doi:10.1055/s-2005-867054.
- Stroup S, Swartz M, Appelbaum P: **Concealed medicines for people with schizophrenia: a U.S. perspective**. *Schizophr Bull* 2002, **28**(3):537-542. doi 10.1093/oxfordjournals.schbul.a006961.
- Svenaeus F: **Do antidepressants affect the self? a phenomenological approach**. *Med Health Care Philos* 2007, **10**(2):153-166. doi:10.1007/s11019-007-9060-8.
- Svenaeus F: **The ethics of self-change: becoming oneself by way of antidepressants or psychotherapy?***Med Health Care Philos* 2009, **12**(2):169-178. doi:10.1007/s11019-009-9190-2.
- Synofzik M: **Effective, indicated--and yet without benefit? the goals of dementia drug treatment and the well-being of the patient. [Wirksam, indiziert--und dennoch ohne nutzen? die ziele der medikamentosen demenz-behandlung und das wohlergehen des patienten]***Z Gerontol Geriatr* 2006, **39**(4):301-307. doi:10.1007/s00391-006-0390-6.
- Tashiro S, Yamada MM, Matsui K: **Ethical issues of placebo-controlled studies in depression and a randomized withdrawal trial in Japan: case study in the ethics of mental health research**. *J Nerv Ment Dis* 2012, **200**(3):255-259. doi:10.1097/NMD.0b013e318247d24f.
- Teboul E: **Keeping 'em honest: the current crisis of confidence in antidepressants.***J Clin Psychiatry* 2011, **72**(7):1015. doi:10.4088/JCP.11lr07111.
- Terbeck S, Chesterman LP: **Will there ever be a drug with no or negligible side effects? evidence from neuroscience**. *Neuroethics* 2014, **7**(2):189-194. doi:10.1007/s12152-013-9195-7.
- Torrey EF: **A question of disclosure**. *Psychiatr Serv* 2008, **59**(8):935. doi:10.1176/appi.ps.59.8.935.
- Valverde MA. **Un dilema bioético a propósito de los antipsicóticos [A bioethical dilemma regarding antipsychotics]**. *Revista De Bioética y Derecho* 2010, **20**:4-9.
- Vincent NA: **Restoring responsibility: promoting justice, therapy and reform through direct brain interventions**. *Criminal Law and Philosophy* 2014, **8**(1):21-42. doi:10.1007/s11572-012-9156-y.
- Waller P: **Dealing with uncertainty in drug safety: lessons for the future from sertindole.***Pharmacoepidemiol Drug Saf* 2003, **12**(4):283-287. doi:10.1002/pds.849.
- Waring DR: **The antidepressant debate and the balanced placebo trial design: an ethical analysis**. *Int J Law Psychiatry* 2008, **31**(6):453-462. doi:10.1016/j.ijlp.2008.09.001.
- Werner S: **Physical activity for patients with dementia: respecting autonomy [bewegung bei menschen mit demenz: autonomie respektieren]**. *Pflege Z* 2011, **64**(4):205-206, 208-209.
- Williams, KG: (2002). **Involuntary antipsychotic treatment: legal and ethical issues**. *Am J Health Syst Pharm* 2002, **59**(22):2233-2237.
- Wong JG, Poon Y, Hui EC: **"I can put the medicine in his soup, doctor!"***J Med Ethics* 2005, **31**(5):262-265. doi:10.1136/jme.2003.007336.
- Yang A, Koo JY: **Non-psychotic uses for anti-psychotics**. *J Drugs Dermatol* 2004, **3**(2):162-168.
- Young SN: **Acute tryptophan depletion in humans: a review of theoretical, practical and ethical aspects**. *J Psychiatry Neurosci* 2013, **38**(5):294-305. doi:10.1503/jpn.120209.
- Zetterqvist AV, Mulinari S: **Misleading advertising for antidepressants in Sweden: a failure of pharmaceutical industry self-regulation**. *PLoS One* 2013, **8**(5):e62609. doi:10.1371/journal.pone.0062609.

Books:

- Biegler P: *The Ethical Treatment of Depression: Autonomy Through Psychotherapy.* Cambridge, Mass.: MIT Press; 2011.
- Dworkin RW: *Artificial Happiness: The Dark Side of the New Happy Class*. New York: Carroll & Graf; 2006.
- Medawar C, Hardon A: *Medicines Out of Control? Antidepressants and the Conspiracy of Goodwill*. The Netherlands: Aksant; 2004.
- Miravalle J: *The Drug, the Soul, and God: A Catholic Moral Perspective on Antidepressants*. Scranton, Pa.: University of Scranton Press; 2010.

Book chapters:

- Foddy B, Kahane G, Savulescu J: **Practical neuropsychiatric ethics**. In *The Oxford Handbook of Philosophy and Psychiatry*. Edited by K.W.M. Fulford, Martin Davies, Richard G.T. Gipps, George Graham, John Z. Sadler, Giovanni Stanghellini, and Tim Thornton. Oxford: Oxford University Press; 2014:1185-1201.
- Stingl AI, Weiss SM: **Beyond and before the label: the ecologies and agencies of ADHD**. In *Krankheitskonstruktionen und Krankheitstreiberei: Die Renaissance der soziologischen Psychiatriekritik*. Edited by Michael Dellwing, Martin Harbusch. Wiesbaden: Springer; 2013:201-231.

***Anti-anxiety agents:***

- Dhahan PS, Mir R: **The benzodiazepine problem in primary care: the seriousness and solutions**. *Qual Prim Care* 2005, **13**(4): 221-224.
- Donate-Bartfield E, Spellecty R, Shane NJ: **Maximizing beneficence and autonomy: ethical support for the use of nonpharmacological methods for managing dental anxiety**. *J Am Coll Dent* 2010, **77**(3): 26-34.
- Glass KC: **Rebuttal to Dr. Streiner: can the “evil” in the “lesser of 2 evils” be justified in placebo-controlled trials?***Can J Psychiatry* 2008, **53**(7): 433.
- Gutheil TG: **Reflections on ethical issues in psychopharmacology: an American perspective**. *Int J Law Psychiatry* 2012, **35**(5-6): 387-391. doi: 10.1016/j.ijlp.2012.09.007.
- Hofsø K, Coyer FM: **Part 1. Chemical and physical restraints in the management of mechanically ventilated patients in the ICU: contributing factors**. *Intensive Crit Care Nurs* 2007, **23**(5): 249-255. doi: 10.1016/j.iccn.2007.04.003.
- Kaut KP: **Psychopharmacology and mental health practice: an important alliance**. *J Ment Health Couns* 2011, **33**(3): 196-222. doi: 10.17744/mehc.33.3.u357803u508r4070.
- King JH, Anderson SM: **Therapeutic implications of pharmacotherapy: current trends and ethical issues**. *J Couns Dev* 2004, **82**(3): 329-336. doi: 10.1002/j.1556-6678.2004.tb00318.x.
- Kirmayer LJ: **Psychopharmacology in a globalizing world: the use of anti-depressants in Japan**. *Transcult Psychiatry* 2002, **39**(3): 295-322. doi: 10.1177/136346150203900302.
- Klemperer D: **Drug research: marketing before evidence, sales before safety**. *Dtsch Arztebl Int* 2010, **107**(16): 277-278. doi: 10.3238/arztebl.2010.0277.
- Kotzalidis G et al.: **Ethical questions in human clinical psychopharmacology: should the focus be on placebo administration?***J Psychopharmacol* 2008, **22**(6): 590-597. doi: 10.1177/0269881108089576.
- Levy N, Clarke S: **Neuroethics and psychiatry**. *Curr Opin Psychiatry* 2008, **21**(6): 568-571. doi: 10.1097/YCO.0b013e3283126769.
- Mohamed AD, Sahakian BJ: **The ethics of elective psychopharmacology**. *Int J Neuropsychopharmacol* 2012, **15**(4): 559-571. doi: 10.1017/S146114571100037X.
- Murray CE, Murray TL: **The family pharm: an ethical consideration of psychopharmacology in couple and family counseling**. *Fam J Alex Va* 2007, **15**(1): 65-71. doi: 10.1177/1066480706294123.
- Nierenberg AA et al.: **Critical thinking about adverse drug effects: lessons from the psychology of risk and medical decision-making for clinical psychopharmacology.***Psychother Psychosom* 2008, **77**(4): 201-208. doi: 10.1159/000126071.
- Sabin JA, Daniels N, Teagarden JR: **The perfect storm**. *Psychiatr Ann* 2004, **34(**2): 125-132. doi: 10.3928/0048-5713-20040201-10.
- Schott G et al.: **The financing of drug trials by pharmaceutical companies and its consequences. part 1: a qualitative, systematic review of the literature on possible influences on the findings, protocols, and quality of drug trials**. *Dtsch Arztebl Int* 2010, **107**(16): 279-285. doi: 10.3238/arztebl.2010.0279.
- Sprung CL et al.: **End-of-life practices in European intensive care units: the Ethicus Study**. *JAMA* 2003, **290**(6): 790-797. doi: 10.1001/jama.290.6.790.
- Sprung CL et al: **Relieving suffering or intentionally hastening death: where do you draw the line?***Crit Care Med* 2008, **36**(1): 8-13. doi: 10.1097/01.CCM.0000295304.99946.58.
- Streiner DL: **The lesser of 2 evils: the ethics of placebo-controlled trials**. *Can J Psychiatry* 2008, **53**(7): 430-432.
- Strous RD: **Ethical considerations in clinical training, care and research in psychopharmacology**. *Int J Neuropsychopharmacol* 2011, **14**(3): 413-424. doi: 10.1017/S1461145710001112.

Books:

- Tone A: *The Age of Anxiety: A History of America’s Turbulent Affair with Tranquilizers*. New York: Basic Books; 2009.

***Analgesics (and pain medicine):***

- Atkinson TJ, Schatman ME, Fudin J: **The damage done by the war on opioids: the pendulum has swung too far**. *J Pain Res* 2014, **7**: 265-268. doi: 10.2147/JPR.S65581.
- Basta LL: **Ethical issues in the management of geriatric cardiac patients: a hospital’s ethics committee decides to not give analgesics to a terminally ill patient to relieve her pain**. *Am J Geriatr Cardiol* 2005, **14**(3): 150-151. doi: 10.1111/j.1076-7460.2004.02724.x.
- Benyamin RM, Datta S, Falco FJ: **A perfect storm in interventional pain management: regulated, but unbalanced**. *Pain Physician* 2010, **13**(2):109-116.
- Birnie KA et al.: **A practical guide and perspectives on the use of experimental pain modalities with children and adolescents**. *Pain Manag* 2014, **4**(2):97-111. doi:10.2217/pmt.13.72.
- Borasio GD et al.: **Attitudes towards patient care at the end of life: a survey of directors of neurological departments**. [Einstellungen zur Patientenbetreuung in der letzten Lebensphase Eine Umfrage bei neurologischen Chefarzten.] *Nervenarzt,* 2004, **75**(12):1187-1193. doi:10.1007/s00115-004-1751-2.
- Braude HD: **Affecting the body and transforming desire: the treatment of suffering as the end of medicine**. *Philos Psychiatr Psychol* 2012, **19**(4): 265-278. doi:10.1353/ppp.2012.0048.
- Braude H: **Normativity unbound: liminality in palliative care ethics**. *Theor Med Bioeth* 2012, **33**(2): 107-122. doi:10.1007/s11017-011-9200-2.
- Braude HD: **Unraveling the knot of suffering: combining neurobiological and hermeneutical approaches**. *Philos Psychiatr Psychol* 2012, 19(4): 291-294. doi: 10.1353/ppp.2012.0056.
- Brennan PM, Whittle IR: **Intrathecal baclofen therapy for neurological disorders: a sound knowledge base but many challenges remain**. *Br J Neurosurg* 2008, **22**(4): 508-519. doi:10.1080/02688690802233364.
- Buchbinder M: **Personhood diagnostics: personal attributes and clinical explanations of pain**. *Med Anthropol Q* 2011, **25**(4): 457-478. doi: 10.1111/j.1548-1387.2011.01180.x.
- Chaturvedi SK: **Ethical dilemmas in palliative care in traditional developing societies, with special reference to the Indian setting**. *J Med Ethics* 2008, **34**(8): 611-615. doi:10.1136/jme.2006.018887.
- Ciuffreda MC et al.: **Rat experimental model of myocardial ischemia/reperfusion injury: an ethical approach to set up the analgesic management of acute post-surgical pain**. *PloS One* 2014, **9**(4): e95913. doi:10.1371/journal.pone.0095913.
- Darnall BD, Schatman ME: **Urine drug screening: opioid risks preclude complete patient autonomy**. *Pain Med* 2014, **15**(12): 2001-2002. doi:10.1111/pme.12604_4.
- de la Fuente-Fernandez R: **Placebo, efecto placebo y ensayos clinicos [Placebo, placebo effect and clinical trials].***Neurologia* 2007, **22**(2): 69-71.
- Derbyshire SW: **Foetal pain?***Best Pract Res Clin Obstet Gynaecol* 2010, **24**(5): 647-655. doi:10.1016/j.bpobgyn.2010.02.013.
- Douglas C, Kerridge I, Ankeny R: **Managing intentions: the end-of-life administration of analgesics and sedatives, and the possibility of slow euthanasia**. *Bioethics* 2008, **22**(7): 388-396. doi:10.1111/j.1467-8519.2008.00661.x.
- England JD, Franklin GM: **Difficult decisions: managing chronic neuropathic pain with opioids**. *Continuum (Minneap Minn)* 2012, **18**(1): 181-184. doi:10.1212/01.CON.0000411547.51324.38.
- Feen E: **Continuous deep sedation: consistent with physician's role as healer**. *Am J Bioeth* 2011, **11**(6): 49-51. doi:10.1080/15265161.2011.578200.
- Finkel AG: **Conflict of interest or productive collaboration? the pharma: academic relationship and its implications for headache medicine**. *Headache* 2006, **46**(7): 1181-1185. doi: 10.1111/j.1526-4610.2006.00508.x.
- Franklin GM: **Primum non nocere**. *Pain Med* 2013, **14**(5): 617-618. doi:10.1111/pme.12120_2.
- Giordano J: **Cassandra 's curse: interventional pain management, policy and preserving meaning against a market mentality**. *Pain Physician* 2006, **9**(3): 167-169.
- Giordano J: **Changing the practice of pain medicine writ large and small through identifying problems and establishing goals**. *Pain Physician* 2006, **9**(4): 283-285.
- Giordano J, Schatman ME: **A crisis in chronic pain care: an ethical analysis; part two: proposed structure and function of an ethics of pain medicine**. *Pain Physician* 2008, **11**(5): 589-595.
- Giordano J, Schatman ME: **A crisis in chronic pain care: an ethical analysis; part three: toward an integrative, multi-disciplinary pain medicine built around the needs of the patient**. *Pain Physician* 2008, **11**(6): 775-784.
- Giordano J, Engebretson JC, Benedikter R: **Culture, subjectivity, and the ethics of patient-centered pain care**. *Camb Q Healthc Ethics* 2009, **18**(1): 47-56. doi:10.1017/S0963180108090087.
- Giordano J, Schatman ME: **An ethical analysis of crisis in chronic pain care: facts, issues and problems in pain medicine; part I**. *Pain Physician* 2008, **11**(4): 483-490.
- Giordano J, Schatman ME, Hover G: **Ethical insights to rapprochement in pain care: bringing stakeholders together in the best interest(s) of the patient**. *Pain Physician* 2009, **12**(4): E265-E75.
- Giordano J: **Ethics of, and in, pain medicine: constructs, content, and contexts of application**. *Pain Physician* 2008, **11**(4): 391-392.
- Giordano J: **Hospice, palliative care, and pain medicine: meeting the obligations of non-abandonment and preserving the personal dignity of terminally III patients**. *Del Med J* 2006, **78**(11): 419-422.
- Giordano J: **Moral agency in pain medicine: philosophy, practice and virtue**. *Pain Physician* 2006, **9**(1): 41-46.
- Giordano J, Gomez CF, Harrison C: **On the potential role for interventional pain management in palliative care**. *Pain Physician* 2007, **10**(3): 395-398.
- Giordano J, Abramson K, Boswell MV: **Pain assessment: subjectivity, objectivity, and the use of neurotechnology**. *Pain Physician* 2010, **13**(4): 305-315.
- Giordano J, Boswell MV: **Pain, placebo, and nocebo: epistemic, ethical, and practical issues**. *Pain Physician* 2005, **8**(4): 331-333.
- Giordano J: **Pain research: can paradigmatic expansion bridge the demands of medicine, scientific philosophy and ethics?***Pain Physician* 2004, **7**(4): 407-410.
- Giordano J, Benedikter R: **The shifting architectonics of pain medicine: toward ethical realignment of scientific, medical and market values for the emerging global community--groundwork for policy**. *Pain Med* 2011, **12**(3): 406-414. doi:10.1111/j.1526-4637.2011.01055.x.
- Giordano J: **Techniques, technology and tekne: the ethical use of guidelines in the practice of interventional pain management**. *Pain Physician* 2007, **10**(1): 1-5.
- Goy ER, Carter JH, Ganzini L: **Parkinson disease at the end of life: caregiver perspectives**. *Neurology* 2007, **69**(6): 611-612. doi: 10.1212/01.wnl.0000266665.82754.61.
- Gupta A, Giordano J: **On the nature, assessment, and treatment of fetal pain: neurobiological bases, pragmatic issues, and ethical concerns**. *Pain Physician* 2007, **10**(4): 525-532.
- Hall JK, Boswell MV: **Ethics, law, and pain management as a patient right**. *Pain Physician* 2009, **12**(3), 499-506.
- Hofmeijer J et al. **Appreciation of the informed consent procedure in a randomised trial of decompressive surgery for space occupying hemispheric infarction**. *J Neurol Neurosurg Psychiatry* 2007, **78**(10):1124-1128. doi: 10.1136/jnnp.2006.110726.
- Hunsinger M et al.: **Disclosure of authorship contributions in analgesic clinical trials and related publications: ACTTION systematic review and recommendations**. *Pain* 2014, **155**(6): 1059-1063. doi:10.1016/j.pain.2013.12.011.
- Jacobson PL, Mann JD: **Evolving role of the neurologist in the diagnosis and treatment of chronic noncancer pain.***Mayo Clin Proc* 2003, **78**(1): 80-84. doi: 10.4065/78.1.80.
- Jacobson PL, Mann JD: **The valid informed consent-treatment contract in chronic non-cancer pain: its role in reducing barriers to effective pain management**. *Compr Ther* 2004, **30**(2): 101-104.
- Jung B, Reidenberg MM: **Physicians being deceived**. *Pain Med* 2007, **8**(5): 433-437. doi: 10.1111/j.1526-4637.2007.00315.x.
- Kotalik J: **Controlling pain and reducing misuse of opioids: ethical considerations**. *Can Fam Physician* 2012, **58**(4): 381-385.
- LeBourgeois HW 3rd, Foreman TA, Thompson JW Jr.: **Novel cases: malingering by animal proxy**. *J Am Acad Psychiatry Law* 2002, **30**(4): 520-524.
- Lebovits A: **Physicians being deceived: whose responsibility?***Pain Med* 2007, **8**(5): 441. doi: 10.1111/j.1526-4637.2007.00337.x.
- Lebovits A: **On the impact of the "business" of pain medicine on patient care: an introduction**. *Pain Med* 2011, **12**(5): 761-762. doi:10.1111/j.1526-4637.2011.01111.x.
- Leo RJ, Pristach CA, Streltzer J: **Incorporating pain management training into the psychiatry residency curriculum**. *Acad Psychiatry* 2003, **27**(1):1-11. doi:10.1176/appi.ap.27.1.1.
- Mancuso T, Burns J: **Ethical concerns in the management of pain in the neonate**. *Paediatr Anaesth* 2009, **19**(10): 953-957. doi:10.1111/j.1460-9592.2009.03144.x.
- McGrew M, Giordano J: **Whence tendance? accepting the responsibility of care for the chronic pain patient**. *Pain Physician* 2009, **12**(3): 483-485.
- Monroe TB, Herr KA, Mion LC, Cowan RL: **Ethical and legal issues in pain research in cognitively impaired older adults**. *Int J Nurs Stud* 2013, **50**(9): 1283-1287. doi:10.1016/j.ijnurstu.2012.11.023.
- Nagasako EM, Kalauokalani DA: **Ethical aspects of placebo groups in pain trials: lessons from psychiatry**. *Neurology* 2005, **65**(12 Suppl 4): S59-S65. doi: 10.1212/WNL.65.12_suppl_4.S59.
- Niebroj LT, Jadamus-Niebroj D, Giordano J: **Toward a moral grounding of pain medicine: consideration of neuroscience, reverence, beneficence, and autonomy**. *Pain Physician* 2008, **11**(1): 7-12.
- Novy DM, Ritter LM, McNeill J: **A primer of ethical issues involving opioid therapy for chronic nonmalignant pain in a multidisciplinary setting**. *Pain Med* 2009, **10**(2): 356-363. doi: 10.1111/j.1526-4637.2008.00509.x.
- Peppin J: **Preserving beneficence**. *Pain Med* 2013, **14**(5): 619. doi:10.1111/pme.12120_3.
- Petersen GL et al.: **The magnitude of nocebo effects in pain: a meta-analysis**. *Pain* 2014, **155**(8): 1426-1434. doi:10.1016/j.pain.2014.04.016.
- Rapoport AM: **More on conflict of interest from a clinical professor of neurology in private practice at a headache center**. *Headache* 2006, **46**(6): 1020-1021. doi:10.1111/j.1526-4610.2006.00474_2.x.
- Robbins NM, Chaiklang K, Supparatpinyo K: **Undertreatment of pain in HIV+ adults in Thailand**. *J Pain Symptom Manage* 2012, **45**(6): 1061-1072. doi:10.1016/j.jpainsymman.2012.06.010.
- Rowbotham MC: **The impact of selective publication on clinical research in pain**. *Pain* 2008, **140**(3), 401-404. doi:10.1016/j.pain.2008.10.026.
- Russell JA, Williams MA, Drogan O: **Sedation for the imminently dying: survey results from the AAN ethics section**. *Neurology* 2010, **74**(16): 1303-1309. doi:10.1212/WNL.0b013e3181d9edcb.
- Schatman ME, Darnall BD: **Among disparate views, scales tipped by the ethics of system integrity**. *Pain Med* 2013, **14**(11): 1629-1630. doi:10.1111/pme.12257_4.
- Schatman ME, Darnall BD: **Commentary and rapprochement**. *Pain Med* 2013, **14**(5): 619-620. doi:10.1111/pme.12120_4.
- Schatman ME, Darnall BD: **Ethical pain care in a complex case**. *Pain Med* 2013, **14**(6): 800-801. doi:10.1111/pme.12137_3.
- Schatman ME, Darnall BD: **A pendulum swings awry: seeking the middle ground on opioid prescribing for chronic non-cancer pain**. *Pain Med* 2013, **14**(5): 617. doi:10.1111/pme.12120.
- Schatman ME, Darnall BD: **A practical and ethical solution to the opioid scheduling conundrum**. *J Pain Res* 2013, **7**:1-3. doi:10.2147/JPR.S58148.
- Schatman ME: **The role of the health insurance industry in perpetuating suboptimal pain management**. *Pain Med* 2011, **12**(3): 415-426. doi:10.1111/j.1526-4637.2011.01061.x.
- Schofferman J: **Interventional pain medicine: financial success and ethical practice: an oxymoron?***Pain Med* 2006, **7**(5): 457-460. doi: 10.1111/j.1526-4637.2006.00215_1.x.
- Schofferman J: **PRF: too good to be true?***Pain Med* 2006, **7**(5): 395. doi: 10.1111/j.1526-4637.2006.00209.x.
- Sullivan M, Ferrell B: **Ethical challenges in the management of chronic nonmalignant pain: negotiating through the cloud of doubt**. *J Pain* 2005, **6**(1): 2-9. doi: 10.1016/j.jpain.2004.10.006.
- Tait RC, Chibnall JT: **Racial/ethnic disparities in the assessment and treatment of pain: psychosocial perspectives**. *Am Psychol* 2014, **69**(2): 131-141. doi:10.1037/a0035204.
- Tracey I: **Getting the pain you expect: mechanisms of placebo, nocebo and reappraisal effects in humans**. *Nat Med* 2010, **16**(11): 1277-1283. doi:10.1038/nm.2229.
- Verhagen AA et al.: **Analgesics, sedative and neuromuscular blockers as part of end-of-life decisions in Dutch NICUs**. *Arch Dis Child Fetal Neonatal Ed* 2009, **94**(6): F434-F438. doi:10.1136/adc.2008.149260.
- Williams MA, Rushton CH: **Justified use of painful stimuli in the coma examination: a neurologic and ethical rationale**. *Neurocrit Care* 2009, **10**(3): 408-413. doi:10.1007/s12028-009-9196-x.

Books:

- Braude HD: *Intuition in Medicine: A Philosophical Defense of Clinical Reasoning*. Chicago: University of Chicago Press; 2012.
- Giordano J: *Pain: Mind, Meaning, and Medicine: Collected Essays on the Philosophical and Ethical Dimensions of Practical Pain Management*. Glen Mills, PA: PPM Communications, Inc.; 2009.
- Giordano J, Boswell MV, eds.: *Pain Medicine: Philosophy, Ethics and Policy*. Yarton, Oxon, UK and Chicago, IL: Linton Atlantic Books; 2009.
- Goldberg DS: *The Bioethics of Pain Management: Beyond Opioids*. New York: Routledge; 2014.
- Schatman ME: *Ethical Issues in Chronic Pain Management*. New York: Informa Healthcare; 2007.

Book chapters:

- Ballantyne JC: **Chronic opioid therapy: the argument for caution**. In *Ethical Issues in Chronic Pain Management*. Edited by Michael E. Schatman. New York: Informa Healthcare; 2007:121-142.
- Cole BE: **Chronic opioid therapy: the argument for opioid therapy to treat persistent noncancer pain**. In *Ethical Issues in Chronic Pain Management*. Edited by Michael E. Schatman. New York: Informa Healthcare; 2007: 111-120.
- Schatman ME: **The demise of the multidisciplinary chronic pain management clinic: bioethical perspectives on providing optimal treatment when ethical principles collide**. In his *Ethical Issues in Chronic Pain Management*. New York: Informa Healthcare; 2007: 43-62.
- Skinner A: **Pain, ethics and research**. In *Pain Management: From Basics to Clinical Practice*. Edited by John Hughes. Edinburgh: Churchill Livingstone/Elsevier; 2008: 277-284.

***Brain stimulation/Neuromodulation:***

***Neurofeedback:***

- Bakhshayesh AR, et al.: **Neurofeedback in ADHD: a single-blind randomized controlled trial**. *Eur Child Adolesc Psychiatry* 2011, **20**(9): 481-491. doi:10.1007/s00787-011-0208-y.
- Focquaert F: **Mandatory neurotechnological treatment: ethical issues**. *Theor Med Bioeth* 2014, **35**(1): 59-72. doi:10.1007/s11017-014-9276-6.
- Ford PJ, Henderson JM: **The clinical and research ethics of neuromodulation**. *Neuromodulation* 2006, **9**(4): 250-252. doi:10.1111/j.1525-1403.2006.00076.x.
- Gevensleben H, et al.: **Neurofeedback for ADHD: further pieces of the puzzle**. *Brain Topogr* 2014, **27**(1): 20-32. doi:10.1007/s10548-013-0285-y.
- Giordano J, DuRousseau D: **Toward right and good use of brain-machine interfacing neurotechnologies: ethical issues and implications for guidelines and policy**. *Cog Technol* 2011, **15**(2):5-10.
- Glannon W: **Neuromodulation, agency and autonomy**. *Brain Topogr* 2014, **27**(1): 46-54. doi:10.1007/s10548-012-0269-3.
- Hammond DC, et al.: **Standards of practice for neurofeedback and neurotherapy: a position paper of the International Society for Neurofeedback & Research**. *J Neurother* 2011, **15**(1):54-64. doi: 10.1080/10874208.2010.545760.
- Hammond DC, Kirk, L: **First, do no harm: adverse effects and the need for practice standards in neurofeedback**. *J Neurother* 2008, **12**(1): 79-88. doi: 10.1080/10874200802219947.
- Huggins JE, Wolpaw JR: **Papers from the Fifth International Brain-computer Interface Meeting: preface**. *J Neural Eng* 2014, **11**(3): 030301. doi:10.1088/1741-2560/11/3/030301.
- Huster RJ, Mokom ZN, Enriquez-Geppert S, Herrmann CS: **Brain-computer interfaces for EEG neurofeedback: peculiarities and solutions**. *Int J Psychophysiol* 2014, **91**(1): 36-45. doi:10.1016/j.ijpsycho.2013.08.011.
- Levy RM: **Ethical issues in neuromodulation**. *Neuromodulation* 2010, **13**(3):147-151. doi:10.1111/j.1525-1403.2010.00281.x.
- Mandarelli G, Moscati FM, Venturini P, Ferracuti S: **Informed consent and neuromodulation techniques for psychiatric purposes: an introduction. [Il consenso informato e gli interventi di neuromodulazione chirurgica in psichiatria: un'introduzione].***Riv Psichiatr* 2013, **48**(4): 285-292. doi:10.1708/1319.14624.
- Maurizio S, et al.: **Differential EMG biofeedback for children with ADHD: a control method for neurofeedback training with a case illustration**. *Appl Psychophysiol Biofeedback* 2013, **38**(2) 109-119. doi:10.1007/s10484-013-9213-x.
- Micoulaud-Franchi JA, Fond G, Dumas G: **Cyborg psychiatry to ensure agency and autonomy in mental disorders: a proposal for neuromodulation therapeutics**. *Front Hum Neurosci* 2013, **7**: 463. doi:10.3389/fnhum.2013.00463.
- Myers JE, Young JS: **Brain wave biofeedback: benefits of integrating neurofeedback in counseling**. *J Couns Dev* 2012, **90**(1): 20-28. doi: 10.1111/j.1556-6676.2012.00003.x.
- Plischke H, DuRousseau D, Giordano J: **EEG-based neurofeedback: the promise of neurotechnology and the need for neuroethically informed guidelines and policies**. *Ethics in Biology, Engineering and Medicine: An International Journal* 2011, **2**(3):221-232. doi:10.1615/EthicsBiologyEngMed.2012004853.
- Rothenberger A, Rothenberger LG: **Updates on treatment of attention-deficit/hyperactivity disorder: facts, comments, and ethical considerations**. *Curr Treat Options Neurol* 2012, **14**(6):594-607. doi:10.1007/s11940-012-0197-2.
- Rusconi E, Mitchener-Nissen T: **The role of expectations, hype and ethics in neuroimaging and neuromodulation futures**. *Front Syst Neurosci* 2014, **8**:214. doi:10.3389/fnsys.2014.00214.
- Vuilleumier P, Sander D, Baertschi B: **Changing the brain, changing the society: clinical and ethical implications of neuromodulation techniques in neurology and psychiatry**. *Brain Topogr* 2014, **27**(1):1-3. doi:10.1007/s10548-013-0325-7.

Book chapters:

- Hammond DC: **Definitions, standard of care, and ethical considerations**. In *Clinical Neurotherapy: Application of Techniques for Treatment*. Edited by David S. Cantor, James R. Evans. London: Elsevier; 2014: 1-18.
- Linden D: **Ethics of neurofeedback**. In his *Brain Control: Developments in Therapy and Implications for Society*. New York: Palgrave Macmillan; 2014: 167.
- Striefel S: **Ethics in neurofeedback practice**. In *Introduction to Quantitative EEG and Neurofeedback: Advanced Theory and Applications*. Edited by Thomas H. Budzynski et al. Amsterdam; Boston: Elsevier; 2009: 475-492.

**Transcranial Electrical Stimulation/Magnetic Stimulation (tDCS, tACS, TMS):**

- Bestmann S, Feredoes E: **Combined neurostimulation and neuroimaging in cognitive neuroscience: past, present, and future**. *Ann N Y Acad Sci* 2013, **1296**:11-30. doi: 10.1111/nyas.12110.
- Brunelin J, Levasseur-Moreau J, Fecteau S: **Is it ethical and safe to use non-invasive brain stimulation as a cognitive and motor enhancer device for military services? a reply to Sehm and Ragert**. *Front HumNeurosci* 2013, **7**: 874. doi:10.3389/fnhum.2013.00874.
- Brunoni AR et al.: **Clinical research with transcranial direct current stimulation (tDCS): challenges and future directions**. *Brain Stim* 2012, **5**(3): 175-195. doi: 10.1016/j.brs.2011.03.002.
- Cabrera LY, Evans EL, Hamilton RH: **Ethics of the electrified mind: defining issues and perspectives on the principled use of brain stimulation in medical research and clinical care**. *Brain Topogr* 2014, **27**(1): 33-45. doi:10.1007/s10548-013-0296-8.
- Cherney LR et al.: **Transcranial direct current stimulation and aphasia: the case of Mr. C**. *Top Stroke Rehabil* 2013, **20**(1):5-21. doi:10.1310/tsr2001-5.
- Chi RP, Snyder AW: **Facilitate insight by non-invasive brain stimulation**. *PloS One* 2011, **6**(2): e16655. doi:10.1371/journal.pone.0016655.
- Cohen Kadosh R et al.: **The neuroethics of non-invasive brain stimulation**. *Curr Biol* 2012, **22**(4):R108-R111. doi:10.1016/j.cub.2012.01.013.
- Coman A, Skarderud F, Reas DL, Hofmann BM: **The ethics of neuromodulation for anorexia nervosa: a focus on tRMS**. *J Eat Disord* 2014, **2**(1):10. doi: 10.1186/2050-2974-2-10.
- Davis NJ, Gold E, Pascual-Leone A, Bracewell RM: **Challenges of proper placebo control for non-invasive brain stimulation in clinical and experimental applications**. *Eur J Neurosci* 2013, **38**(7):2973-2977. doi:10.1111/ejn.12307.
- Davis NJ: **Transcranial stimulation of the developing brain: a plea for extreme caution**. *Front Hum Neurosci* 2014, **8**:600. doi:10.3389/fnhum.2014.00600.
- Davis NJ, van Koningsbruggen MG: **"Non-invasive" brain stimulation is not non-invasive**. *Front Syst Neurosci* 2013, **7**:76. doi:10.3389/fnsys.2013.00076.
- Dranseika V, Gefenas E, Noreika S: **The map of neuroethics**. *Problemos* 2009, **76**:66-73.
- Dubljević V, Saigle V, Racine E: **The rising tide of tDCS in the media and academic literature**. *Neuron* 2014, **82**(4):731-736. doi: 10.1016/j.neuron.2014.05.003.
- Gilbert DL et al.: **Should transcranial magnetic stimulation research in children be considered minimal risk?***Clin Neurophysiol* 2004, **115**(8): 1730-1739. 10.1016/j.clinph.2003.10.037.
- Heinrichs JH: **The promises and perils of non-invasive brain stimulation**. *Int J Law Psychiatry* 2012, **35**(2): 121-129. doi: 10.1016/j.ijlp.2011.12.006.
- Horng SH, Miller FG: **Placebo-controlled procedural trials for neurological conditions**. *Neurotherapeutics* 2007, **4**(3): 531-536. doi: 10.1016/j.nurt.2007.03.001.
- Horvath JC, Carter O, Forte JD: **Transcranial direct current stimulation: five important issues we aren't discussing (but probably should be)**. *Front Syst Neurosci* 2014, **8**: 2. doi:10.3389/fnsys.2014.00002.
- Horvath JC et al.: **Transcranial magnetic stimulation: a historical evaluation and future prognosis of therapeutically relevant ethical concerns**. *J Med Ethics* 2011, **37**(3): 137-143. doi:10.1136/jme.2010.039966.
- Illes J, Gallo M, Kirschen MP: **An ethics perspective on transcranial magnetic stimulation (TMS) and human neuromodulation**. *Behav Neurol* 2006, **17**(3-4): 3-4. doi: 10.1155/2006/791072.
- Johnson MD et al.: **Neuromodulation for brain disorders: challenges and opportunities**. *IEEE Trans Biomed.Eng* 2013, **60**(3): 610-624. doi: 10.1109/TBME.2013.2244890.
- Jones LS: **The ethics of transcranial magnetic stimulation**. *Science* 2007, **315**(5819):1663-1664. doi: 10.1126/science.315.5819.1663c.
- Jorge RE, Robinson RG: **Treatment of late-life depression: a role of non-invasive brain stimulation techniques**. *Int Rev Psychiatry* 2011, **23**(5): 437-444. doi:10.3109/09540261.2011.633501.
- Jotterand F, Giordano J: **Transcranial magnetic stimulation, deep brain stimulation and personal identity: ethical questions, and neuroethical approaches for medical practice**. *Int Rev Psychiatry* 2011, **23**(5): 476-485. doi: 10.3109/09540261.2011.616189.2011.616189.
- Karim AA: **Transcranial cortex stimulation as a novel approach for probing the neurobiology of dreams: clinical and neuroethical implications**. *IJODR* 2010, **3**(1): 17-20. doi: 10.11588/ijodr.2010.1.593.
- Keiper A: **The age of neuroelectronics**. *New Atlantis* 2006, **11**:4-41.
- Knoch D et al.: **Diminishing reciprocal fairness by disrupting the right prefrontal cortex**. *Science* 2006, **314**(5800): 829-832. doi: 10.1126/science.1129156.
- Krause B, Cohen Kadosh R: **Can transcranial electrical stimulation improve learning difficulties in atypical brain development? a future possibility for cognitive training**. *Dev Cogn Neurosci* 2013, **6**: 176-194. doi:10.1016/j.dcn.2013.04.001.
- Levasseur-Moreau J, Brunelin J, Fecteau S: **Non-invasive brain stimulation can induce paradoxical facilitation: are these neuroenhancements transferable and meaningful to security services**? *Front HumNeurosci* 2013, **7**: 449. doi:10.3389/fnhum.2013.00449.
- Levy N: **Autonomy is (largely) irrelevant**. *Am J Bioeth* 2009, **9**(1): 50-51. doi: 10.1080/15265160802588228.
- Luber B et al.: **Non-invasive brain stimulation in the detection of deception: scientific challenges and ethical consequences**. *Behav Sci Law* 2009, **27**(2):191-208. doi:10.1002/bsl.860.
- Najib U, Horvath JC: **Transcranial magnetic stimulation (TMS) safety considerations and recommendations**. *Neuromethods* 2014, **89**: 15-30. doi: 10.1007/978-1-4939-0879-0_2.
- Nitsche MA, et al.: **Safety criteria for transcranial direct current stimulation (tDCS) in humans.***Clin Neurophysiol* 2003, **114**(11): 2220-2222. doi: 10.1016/S1388-2457(03)00235-9.
- Nyffeler T, Müri R: **Comment on: safety, ethical considerations, and application guidelines for the use of transcranial magnetic stimulation in clinical practice and research, by Rossi et al**. *Clin Neurophysiol* 2010, **121**(6): 980. doi: 10.1016/j.clinph.2010.04.001.
- Reiner PB: **Comment on "can transcranial electrical stimulation improve learning difficulties in atypical brain development? a future possibility for cognitive training" by Krause and Cohen Kadosh**. *Dev Cogn Neurosci* 2013, **6**: 195-196. doi:10.1016/j.dcn.2013.05.002.
- Rossi S, Hallett M, Rossini PM, Pascual-Leone A, Safety of TMS Consensus Group: **Safety, ethical considerations, and application guidelines for the use of transcranial magnetic stimulation in clinical practice and research**. *Clin Neurophysiol* 2009, **120**(12): 2008-2039. doi: 10.1016/j.clinph.2009.08.016.
- Schutter DJLG, van Honk J, Panksepp J: **Introducing transcranial magnetic stimulation (TMS) and its property of causal inference in investigating brain-function relationships**. *Synthese* 2004, **141**(2):155-173. doi: 10.1023/B:SYNT.0000042951.25087.16.
- Sehm B, Ragert P: **Why non-invasive brain stimulation should not be used in military and security services**. *Front Hum Neurosci* 2013, **7**:553. doi: 10.3389/fnhum.2013.00553.
- Shamoo AE: **Ethical and regulatory challenges in psychophysiology and neuroscience-based technology for determining behavior**. *Account Res* 2010, **17**(1): 8-29. doi: 10.1080/08989620903520271.
- Shirota Y, Hewitt M, Paulus W: **Neuroscientists do not use non-invasive brain stimulation on themselves for neural enhancement**. *Brain Stimul* 2014, **7**(4): 618-619. doi:10.1016/j.brs.2014.01.061.
- Widdows KC, Davis NJ: **Ethical considerations in using brain stimulation to treat eating disorders**. *Front Behav Neurosci* 2014, **8**: 351. doi:10.3389/fnbeh.2014.00351.
- Williams NR, et al.: **Interventional psychiatry: how should psychiatric educators incorporate neuromodulation into training**? *Acad Psychiatry* 2014, **38**(2): 168-176. doi: 10.1007/s40596-014-0050-x.

Book chapters:

- Ganis G, Rosenfeld JP: **Neural correlates of deception**. In *The Oxford Handbook of Neuroethics*. Edited by Judy Illes, Barbara J. Sahakian. Oxford: Oxford University Press; 2011: 101–118.
- Green RM: **Ethical issues**. In *Handbook of Transcranial Magnetic Stimulation*. Edited by Alvaro Pascual-Leone. London: Arnold, and New York: Oxford University Press; 2002: 50–56.
- Horvath J et al.: **Transcranial magnetic stimulation: future prospects and ethical concerns in treatment and research**. In *Neuroethics in Practice*. Edited by Anjan Chatterjee, Martha J. Farah. New York: Oxford University Press; 2012: 209–234.
- Nuffield Council on Bioethics: **Transcranial brain stimulation**. In its *Novel Neurotechnologies: Intervening in the Brain*. London: Nuffield Council on Bioethics; 2013: 16–22.
- Pascual-Leone A, Fregni F, Steven-Wheeler MS, Forrow L: **Non-invasive brain stimulation as a therapeutic and investigative tool: an ethical appraisal**. In *The Oxford Handbook of Neuroethics*. Edited by Judy Illes, Barbara J. Sahakian. Oxford: Oxford University Press; 2011: 417–440.
- Steven MS, Pascual-Leone A**: Transcranial magnetic stimulation and the human brain: an ethical evaluation**. In *Neuroethics: Defining the Issues in Theory, Practice, and Policy*. Edited by Judy Illes. Oxford: Oxford University Press; 2006: 201–211.

**Deep brain stimulation:**

- Arends M, Fangerau H, Winterer G: **[“Psychosurgery” and deep brain stimulation with psychiatric indication: current and historical aspects**]. *Nervenarzt* 2009, **80**(7): 781-788. doi: 1007/s00115-009-2726-0.
- Baylis F: **“I am who I am”: on the perceived threats to personal identity from deep brain stimulation**. *Neuroethics* 2013, **6**: 513-526. doi: 10.1007/s12152-011-9137-1.
- Bell E, Mathieu G, Racine E: **Preparing the ethical future of deep brain stimulation**. *Surg Neurol* 2009, **72**(6): 577-586. doi: 10.1016/j.surneu.2009.03.029.
- Bell E et al.: **A review of social and relational aspects of deep brain stimulation in Parkinson's disease informed by healthcare provider experiences**. *Parkinsons Dis* 2011, **2011**:871874. doi: 10.4061/2011/871874.
- Bell E et al.: **Deep brain stimulation and ethics: perspectives from a multisite qualitative study of Canadian neurosurgical centers**. *World Neurosurg* 2011, **76**(6): 537-547. doi: 10.1016/j.wneu.2011.05.033.
- Bell E et al.: **Hope and patients’ expectations in deep brain stimulation: healthcare providers’ perspectives and approaches**. *J Clin Ethics* 2010, **21**(2): 112-124.
- Bell E, Racine E: **Deep brain stimulation, ethics, and society**. *J Clin Ethics* 2010, **21**(2): 101-103.
- Bell E, Racine E: **Clinical and ethical dimensions of an innovative approach for treating mental illness: a qualitative study of health care trainee perspectives on deep brain stimulation**. *Can J Neurosci Nurs* 2013, **35**(3): 23-32.
- Bell E, Racine E: **Ethics guidance for neurological and psychiatric deep brain stimulation**. *Handb Clin Neurol* 2013, **116**: 313-325. doi: 10.1016/B978-0-444-53497-2.00026-7.
- Bell E et al.: **Beyond consent in research: revisiting vulnerability in deep brain stimulation for psychiatric disorders**. *Camb Q Healthc Ethics* 2014, **23**(3): 361-368. doi: 10.1017/S0963180113000984.
- Canavero S: **Halfway technology for the vegetative state**. *Arch Neurol* 2010, **67**(6): 777. doi: 10.1001/archneurol.2010.102.
- Christen M, Müller S: **Current status and future challenges of deep brain stimulation in Switzerland**. *Swiss Med Wkly* 2012, **142**: w13570. doi: 10.4414/smw.2012.13570.
- Clausen J: **Ethical brain stimulation- neuroethics of deep brain stimulation in research and clinical practice**. *Eur J Neurosci* 2010, **32**(7): 1152-1162. doi: 10.1111/j.1460-9568.2010.07421.x.
- de Zwaan M, Schlaepfer TE: **Not too much reason for excitement: deep brain stimulation for anorexia nervosa**. *Eur Eat Disord Rev* 2013, **21**(6): 509-511. doi: 10.1002/erv.2258.
- Dunn LB et al.: **Ethical issues in deep brain stimulation research for treatment-resistant depression: focus on risk and consent**. *AJOB Neurosci* 2011, **2**(1): 29-36. doi: 10.1080/21507740.2010.533638.
- Erickson-Davis C: **Ethical concerns regarding commercialization of deep brain stimulation for obsessive compulsive disorder**. *Bioethics* 2012, **26**(8): 440-446. doi: 10.1111/j.1467-8519.2011.01886.x.
- Farris S, Ford P, DeMarco J, Giroux ML: **Deep brain stimulation and the ethics of protection and caring for the patient with Parkinson’s dementia**. *Mov Disord* 2008, **23**(14): 1973-1976. doi: 10.1002/mds.22244.
- Finns JJ: **Neuromodulation, free will and determinism: lessons from the psychosurgery debate**. *Clin Neurosci Res* 2004, **4**(1/2): 113-118. doi: 10.1016/j.cnr.2004.06.011.
- Finns JJ et al.: **Misuse of the FDA’s humanitarian device exemption in deep brain stimulation for obsessive-compulsive disorder**. *Health Aff (Millwood)* 2011, **30**(2): 302-311. doi: 10.1377/hlthaff.2010.0157.
- Finns JJ, Schiff ND: **Conflicts of interest in deep brain stimulation research and the ethics of transparency**. *J Clin Ethics* 2010, **21**(2): 125-132.
- Finns JJ et al.: **Ethical guidance for the management of conflicts of interest for researchers, engineers and clinicians engaged in the development of therapeutic deep brain stimulation**. *J Neural Eng* 2011, **8**(3): 033001. doi: 10.1088/1741-2560/8/3/033001.
- Giacino J, Finns JJ, Machado A, Schiff ND: **Central thalamic deep brain stimulation to promote recovery from chronic posttraumatic minimally conscious state: challenges and opportunities**. *Neuromodulation* 2012, **15**(4): 339-349. doi: 10.1111/j.1525-1403.2012.00458.x.
- Gilbert F: **The burden of normality: from ‘chronically ill’ to ‘symptom free’: new ethical challenges for deep brain stimulation postoperative treatment**. *J Med Ethics* 2012, **38**(7): 408-412. doi: 10.1136/medethics-2011-100044.
- Gilbert F, Ovadia D: **Deep brain stimulation in the media: over-optimistic portrayals call for a new strategy involving journalists and scientists in ethical debates**. *Front Integr Neurosci* 2011, **5**:16. doi: 10.3389/fnint.2011.00016.
- Glannon W: **Consent to deep brain stimulation for neurological and psychiatric disorders**. *J Clin Ethics* 2010, **21**(2): 104-111.
- Glannon W: **Deep-brain stimulation for depression**. *HEC Forum* 2008, **20**(4): 325-335. doi: 10.1007/s10730-008-9084-3.
- Goldberg DS: **Justice, population health, and deep brain stimulation: the interplay of inequities and novel health technologies**. *AJOB Neurosci* 2012, **3**(1): 16-20. doi: 10.1080/21507740.2011.635626.
- Grant RA et al.: **Ethical considerations in deep brain stimulation for psychiatric illness**. *J Clin Neurosci* 2014, **21**(1): 1-5. doi: 10.1016/j.jocn.2013.04.004.
- Hariz MI, Blomstedt P, Zrinzo L: **Deep brain stimulation between 1947 and 1987: the untold story**. *Neurosurg Focus* 2010, **29**(2): E1. doi: 10.3171/2010.4.FOCUS10106.
- Hinterhuber H: [**Deep brain stimulation-new indications and ethical implications**]. *Neuropsychiatr* 2009, **23**(3): 139-143.
- Hubbeling D: **Registering findings from deep brain stimulation**. *JAMA* 2010, **303**(21): 2139-2140. doi: 10.1001/jama.2010.705.
- Illes J. **Deep brain stimulation: paradoxes and a plea**. *AJOB Neurosci* 2012, **3**(1): 65-70. doi: 10.1080/21507740.2011.635629.
- Johansson V et al.: **Thinking ahead on deep brain stimulation: an analysis of the ethical implications of a developing technology**. *AJOB Neurosci* 2014, **5**(1): 24-33. doi: 10.1080/21507740.2013.863243.
- Jotterand F, Giordano J: **Transcranial magnetic stimulation, deep brain stimulation and personal identity: ethical questions, and neuroethical approaches for medical practice**. *Int Rev Psychiatry* 2011, **23**(5): 476-485. doi: 10.3109/09540261.2011.616189.2011.616189.
- Katayama Y, Fukaya C: [**Deep brain stimulation and neuroethics**]. *Brain Nerve* 2009, **61**(1): 27-32.
- Klaming L, Haselager P: **Did my brain implant make me do it? questions raised by DBS regarding psychological continuity, responsibility for action and mental competence**. *Neuroethics* 2013, **6**: 527-539. doi: 10.1007/s12152-010-9093-1.
- Kraemer F: **Authenticity or autonomy: when deep brain stimulation causes a dilemma**. *J Med Ethics* 2013, **39**(12): 757-760. doi: 10.1136/medethics-2011-100427.
- Kraemer F: **Me, myself and my brain implant: deep brain stimulation raises questions of personal authenticity and alienation**. *Neuroethics* 2013, **6**: 483-497. doi: 10.1007/s12152-011-9115-7.
- Kringelbach ML, Aziz TZ: **Deep brain stimulation: avoiding the errors of psychosurgery**. *JAMA* 2009, **301**(16): 1705-1707. doi: 10.1001/jama.2009.551.
- Kringelbach ML, Aziz TZ: **Neuroethical principles of deep-brain stimulation**. *World Neurosurg* 2011, **76**(6): 518-519. doi: 10.1016/j.wneu.2011.06.042.
- Krug H, Müller O, Bittner U: [**Technological intervention in the self? an ethical evaluation of deep brain stimulation relating to patient narratives**]. *Fortschr Neurol Psychiatr* 2010, **78**(11): 644-651. doi: 10.1055/s-0029-1245753.
- Kubu CS, Ford PJ: **Beyond mere symptom relief in deep brain stimulation: an ethical obligation for multi-faceted assessment of outcome**. *AJOB Neurosci* 2012, **3**(1): 44-49. doi: 10.1080/21507740.2011.633960.
- Kuhn J, Gaebel W, Klosterkoetter J, Woopen C: **Deep brain stimulation as a new therapeutic approach in therapy-resistant mental disorders: ethical aspects of investigational treatment**. *Eur Arch Psychiatry Clin Neurosci* 2009, **259**(Suppl 2): S135-S141. doi: 10.1007/s00406-009-0055-8.
- Kuhn J et al.: **Deep brain stimulation for psychiatric disorders**. *Dtsch Arztebl Int* 2010, **107**(7): 105-113. doi: 10.3238/arztebl.2010.0105.
- Lipsman N, Giacobbe P, Bernstein M, Lozano AM: **Informed consent for clinical trials of deep brain stimulation in psychiatric disease: challenges and implications for trial design**. *J Med Ethics* 2012, **38**(2): 107-111. doi: 10.1136/jme.2010.042002.
- Lipsman N, Glannon W: **Brain, mind and machine: what are the implications of deep brain stimulation for perceptions of personal identity, agency and free will?***Bioethics* 2013, **27**(9): 465-470. doi:10.1111/j.1467-8519.2012.01978.x.
- Mandarelli G, Moscati FM, Venturini P, Ferracuti S: **[Informed consent and neuromodulation techniques for psychiatric purposes: an introduction]**. *Riv Psichiatr* 2013, **48**(4): 285-292. doi: 10.1708/1319.14624.
- Mathews DJ: **Deep brain stimulation, personal identity and policy**. *Int Rev Psychiatry* 2011, **23**(5):486-492. doi:10.3109/09540261.2011.632624.
- Mendelsohn D, Lipsman N, Bernstein M: **Neurosurgeons’ perspectives on psychosurgery and neuroenhancement: a qualitative study at one center**. *J Neurosurg* 2010, **113**(6): 1212-1218. doi: 10.3171/2010.5.JNS091896.
- Meyer FP: **Re: deep brain stimulation for psychiatric disorders: topic for ethics committee**. *Dtsch Arztebl Int* 2010, **107**(37): 644. doi: 10.3238/arztebl.2010.0644b.
- Müller UJ et al.: [**Deep brain stimulation in psychiatry: ethical aspects**]. *Psychiatr Prax* 2014, **41**(Suppl 1): S38-S43. doi: 10.1055/s-0034-1370015.
- Müller S, Walter H, Christen M: **When benefitting a patient increases the risk for harm for third persons- the case of treating pedophilic Parkinsonian patients with deep brain stimulation**. *Int J Law Psychiatry* 2014, **37**(3): 295-303. doi: 10.1016/j.ijlp.2013.11.015.
- Oshima H, Katayama Y: **Neuroethics of deep brain stimulation for mental disorders: brain stimulation reward in humans**. *Neurol Med Chir (Tokyo)* 2010, **50**(9): 845-852. doi: 10.2176/nmc.50.845.
- Pacholczyk A: **DBS makes you feel good! –why some of the ethical objections to the use of DBS for neuropsychiatric disorders and enhancement are not convincing**. *Front Integr Neurosci* 2011, **5**: 14. doi: 10.3389/fnint.2011.00014.
- Patuzzo S, Manganotti P: **Deep brain stimulation in persistent vegetative states: ethical issues governing decision making**. *Behav Neurol* 2014, **2014**: 641213. doi: 10.1155/2014/641213.
- Racine E, Bell E: **Responding ethically to patient and public expectations about psychiatric DBS**. *AJOB Neurosci 2012*, **3**(1): 21-29. doi: 10.1080/21507740.2011.633959.
- Racine E et al.: **“Currents of hope”: neurostimulation techniques in U.S. and U.K. print media**. *Camb Q Healthc Ethics* 2007, **16**(3): 312-316. doi: 10.1017/S0963180107070351.
- Rabins P et al.: **Scientific and ethical issues related to deep brain stimulation for disorders of mood, behavior, and thought**. *Arch Gen Psychiatry* 2009, **66**(9): 931-937. doi: 10.1001/archgenpsychiatry.2009.113.
- Rossi PJ, Okun M, Giordano J: **Translational imperatives in deep brain stimulation research: addressing neuroethical issues of consequences and continuity of clinical care**. *AJOB Neurosci* 2014, **5**(1): 46-48. doi: 10.1080/21507740.2013.863248.
- Schermer M: **Ethical issues in deep brain stimulation**. *Front Integr Neurosci* 2011, **5**:17. doi: 10.3389/fnint.2011.00017.
- Schermer M: **Health, happiness and human enhancement** — **dealing with unexpected effects of deep brain stimulation**. *Neuroethics* 2013, **6**: 435-445. doi: 10.1007/s12152-011-9097-5.
- Schiff ND, Giacino JT, Fins JJ: **Deep brain stimulation, neuroethics, and the minimally conscious state: moving beyond proof of principle**. *Arch Neurol* 2009, **66**(6): 697-702. doi: 10.1001/archneurol.2009.79.
- Schlaepfer TE: **Toward an emergent consensus**— **international perspectives on neuroethics of deep brain stimulation for psychiatric disorders**—**a Tower of Babel?***AJOB Neurosci* 2012, **3**(1): 1-3. doi: 10.1080/21507740.2012.646914.
- Schlaepfer TE, Fins JJ: **Deep brain stimulation and the neuroethics of responsible publishing: when one is not enough**. *JAMA* 2010, **303**(8): 775-776. doi: 10.1001/jama.2010.140.
- Schlaepfer TE, Lisanby SH, Pallanti S: **Separating hope from hype: some ethical implications of the development of deep brain stimulation in psychiatric research and treatment**. *CNS Spectr* 2010, **15**(5): 285-287. doi: 10.1017/S1092852900027504.
- Schmetz MK, Heinemann T: [**Ethical aspects of deep brain stimulation in the treatment of psychiatric disorders**]. *Fortschr Neurol Psychiatr* 2010, **78**(5): 269-278. doi: 10.1055/s-0029-1245208.
- Schmitz-Luhn B, Katzenmeier C, Woopen C: **Law and ethics of deep brain stimulation**. *Int J Law Psychiatry* 2012, **35**(2): 130-136. doi: 10.1016/j.ijlp.2011.12.007.
- Sen AN et al.: **Deep brain stimulation in the management of disorders of consciousness: a review of physiology, previous reports, and ethical considerations**. *Neurosurg Focus* 2010, **29**(2): E14. doi: 10.3171/2010.4.FOCUS1096.
- Sharifi MS: **Treatment of neurological and psychiatric disorders with deep brain stimulation: raising hopes and future challenges**. *Basic Clin Neurosci* 2013, **4**(3): 266-270.
- Skuban T, Hardenacke K, Woopen C, Kuhn J: **Informed consent in deep brain stimulation**—**ethical considerations in a stress field of pride and prejudice**. *Front Integr Neurosci* 2011, **5**:7. doi: 10.3389/fnint.2011.00007.
- Synofzik M: [**Intervening in the neural basis of one’s personality: a practice-oriented ethical analysis of neuropharmacology and deep-brain stimulation**]. *Dtsch Med Wochenschr* 2007, **132**(50): 2711-2713. doi: 10.1055/s-2007-993124.
- Synofzik M: [**New indications for deep brain stimulation: ethical criteria for research and therapy**]. *Nervenarzt* 2013, **84**(10): 1175-1182. doi: 10.1007/s00115-013-3733-8.
- Synofzik M, Schlaepfer TE: **Electrodes in the brain—ethical criteria for research and treatment with deep brain stimulation for neuropsychiatric disorders**. *Brain Stimul* 2011, **4**(1): 7-16. doi: 10.1016/j.brs.2010.03.002.
- Synofzik M, Schlaepfer TE: **Stimulating personality: ethical criteria for deep brain stimulation in psychiatric patients and for enhancement purposes**. *Biotechnol J* 2008, **3**(12): 1511-1520. doi: 10.1002/biot.200800187.
- Synofzik M, Schlaepfer TE, Fins JJ: **How happy is too happy? euphoria, neuroethics, and deep brain stimulation of the nucleus accumbens**. *AJOB Neurosci* 2012, **3**(1): 30-36. doi: 10.1080/21507740.2011.635633.
- Takagi M: [**Safety and neuroethical consideration of deep brain stimulation as a psychiatric treatment**]. *Brain Nerve* 2009, **61**(1): 33-40.
- Weisleder P**: Individual justice or societal injustice**. *Arch Neurol* 2010, **67**(6): 777-778. doi: 10.1001/archneurol.2010.103.
- Wind JJ, Anderson DE: **From prefrontal leukotomy to deep brain stimulation: the historical transformation of psychosurgery and the emergence of neuroethics**. *Neurosurg Focus* 2008, **25**(1): E10. doi: 10.3171/FOC/2008/25/7/E10.
- Witt K et al.: **Deep brain stimulation and the search for identity**. *Neuroethics* 2013, **6**: 499-511. doi: 10.1007/s12152-011-9100-1.
- Woopen C: **Ethical aspects of neuromodulation**. *Int Rev Neurobiol* 2012, **107**: 315-332. doi: 10.1016/B978-0-12-404706-8.00016-4.
- Woopen C et al.: **Early application of deep brain stimulation: clinical and ethical aspects**. *Prog Neurobiol* 2013, **110**: 74-88. doi: 10.1016/j.pneurobio.2013.04.002.

Books:

- Fangerau H, Fegert J, Trapp T: *Implanted Minds: The Neuroethics of Intracerebral Stem Cell Transplantation and Deep Brain Stimulation*. Bielefeld, Germany: Transcript Verlag; 2011.

Book chapters:

- Ackerman S: **Ethical and practical concerns of deep brain stimulation**. In *her Hard Science, Hard Choices: Facts, Ethics and Policies Guiding Brain Science Today*. New York: Dana Press; 2006: 100-102.
- Blank RH: **Brain intervention: state of the art**. In *his Intervention in the Brain: Politics, Policy, and Ethics*. Cambridge, Mass.: MIT Press; 2013: 25-63.
- Bell E, Racine E: **Ethics guidance for neurological and psychiatric deep brain stimulation**. In *Brain Stimulation: Handbook of Clinical Neurology*. Edited by A.M. Lozano, Mark Hallett. Amsterdam: Elsevier; 2013: 313-328.
- Green AL, Pereira EAC, Aziz TZ: **Deep brain stimulation and pleasure**. In *Pleasures of the Brain*. Edited by Morten L. Kringelback and Kent C. Berridge. Oxford: Oxford University Press; 2010: 302-319.
- Matthews DJH, Rabins PV, Greenberg BD: **Deep brain stimulation for treatment-resistant neuropsychiatric disorders**. In *Oxford Handbook of Neuroethics*. Edited by Judy Illes and Barbara J. Sahakian. Oxford: Oxford University Press; 2011: 441-454.
- Synofzik M: **Functional neurosurgery and deep brain stimulation**. In *Neuroethics in Practice*. Edited by Anjan Chatterjee, Martha J. Farah. New York: Oxford University Press; 2013: 189-208.

***Brain-machine interfaces:***

- Baranauskas G: **What limits the performance of current invasive brain machine interfaces?***Front Syst Neurosci* 2014, **8**:68. doi: 10.3389/fnsys.2014.00068.
- Carmichael C, Carmichael P: **BNCI systems as a potential assistive technology: ethical issues and participatory research in the BrainAble project**. *Disabil Rehabil Assist Technol* 2014, **9**(1): 41-47. doi: 10.3109/17483107.2013.867372.
- Clausen J: **Bonding brains to machines: ethical implications of electroceuticals for the human brain**. *Neuroethics* 2013, **6**(3): 429-434. doi: 10.1007/s12152-013-9186-8.
- Clausen J: **Conceptual and ethical issues with brain-hardware interfaces**. *Curr Opin Psychiatry* 2011, **24**(6): 495-501. doi: 10.1097/YCO.0b013e32834bb8ca.
- Clausen J: **Moving minds: ethical aspects of neural motor prostheses**. *Biotechnol J* 2008, **3**(12): 1493-1501. doi: 10.1002/ciot.200800244.
- Demetriades AK, Demetriades CK, Watts C, Ashkan K: **Brain-machine interface: the challenge of neuroethics**. *Surgeon* 2010, **8**(5): 267-269. doi: 10.1016/j.surge.2010.05.006.
- Farah MJ, Wolpe PR: **Monitoring and manipulating brain function: new neuroscience technologies and their ethical implications**. *Hastings Cent Rep* 2004, **34**(3): 35-45. doi: 10.2307/3528418.
- Grübler G: **Beyond the responsibility gap: discussion note on responsibility and liability in the use of brain-computer interfaces**. *AI Soc* 2011, **26**:377-382. doi: 10.1007/s00146-011-0321-y.
- Hansson SO: **Implant ethics**. *J Med Ethics* 2005, **31**(9): 519-525. doi: 10.1136/jme.2004.009803.
- Heersmink R: **Embodied tools, cognitive tools and brain-computer interfaces.***Neuroethics* 2013, **6**(1): 207-219. doi: 10.1007/s12152-011-9136-2.
- Jebari K: **Brain machine interface and human enhancement – an ethical review.***Neuroethics* 2013, **6**(3): 617-625. doi: 10.1007/s12152-012-9176-2.
- Jebari K, Hansson SO: **European public deliberation on brain machine interface technology: five convergence seminars**. *Sci Eng Ethics* 2013, **19**(3): 1071-1086. doi: 10.1007/s11948-012-9425-0.
- Kotchetkov IS et al.: **Brain-computer interfaces: military, neurosurgical, and ethical perspective**. *Neurosurg Focus* 2010, **28**(5): E25. doi: 10.3171/2010.2.FOCUS1027.
- Lucivero F, Tamburrini G: **Ethical monitoring of brain-machine interfaces: a note on personal identity and autonomy**. *AI Soc* 2008, **22**(3): 449-460. doi: 10.1007/s00146-007-0146-x.
- McCullagh P, Lightbody G, Zygierewicz J, Kernohan WG: **Ethical challenges associated with the development and deployment of brain computer interface technology.***Neuroethics* 2014, **7**(2): 109-122. doi: 10.1007/s12152-013-9188-6.
- McGie SC, Nagai MK, Artinian-Shaheen T: **Clinical ethical concerns in the implantation of brain-machine interfaces: part 1: overview, target populations, and alternatives**. *IEEE Pulse* 2013, **4**(1): 28-32. doi: 10.1109/MPUL.2012.2228810.
- McGie SC, Nagai MK, Artinian-Shaheen T: **Clinical ethical concerns in the implantation of brain-machine interfaces**. *IEEE Pulse* 2013, **4**(2): 32-37. doi: 10.1109/MPUL.2013.2242014.
- Mizushima N, Sakura O: **A practical approach to identifying ethical and social problems during research and development: a model for a national research project of brain-machine interface**. *EASTS* 2012, **6**(3): 335-345. doi: 10.1215/18752160-1730938.
- Mizushima N, Sakura O: **Project-based approach to identify the ethical, legal and social implications: a model for national project of Brain Machine Interface development.***Neurosci Res* 2011, **71**(Supp): E391. doi: 10.1016/j.neures.2011.07.1715.
- Nijboer F, Clausen J, Allison, BZ, Haselager P: **The Asilomar Survey: stakesholders’ opinions on ethical issues related to brain-computer interfacing**. *Neuroethics* 2013, **6**: 541-578. doi: 10.1007/s12152-011-9132-6.
- Peterson GR: **Imaging God: cyborgs, brain-machine interfaces, and a more human future.***Dialog* 2005, **44**(4): 337-346. doi: 10.1111/j.0012-2033.2005.00277.x.
- Rowland NC, Breshears J, Chang EF: **Neurosurgery and the dawning age of brain-machine interfaces**. *Surg Neurol Int* 2013, **4**(Suppl 1): S11-S14. doi: 10.4103/2152-7806.109182.
- Rudolph A: **Military: brain machine could benefit millions**. *Nature* 2003, **424**(6947): 369. doi: 10.1038/424369b.
- Sakura O: **Brain-machine interface and society: designing a system of ethics and governance**. *Neurosci Res* 2009, **65**(Supp 1): S33. doi:10.1016/j.neures.2009.09.1687.
- Sakura O, Mizushima N: **Toward the governance of neuroscience: neuroethics in Japan with special reference to brain-machine interface (BMI)**. *EASTS* 2010, **4**(1): 137-144. doi: 10.1007/s12280-010-9121-6.
- Schermer M: **The mind and the machine: on the conceptual and moral implications of brain-machine interaction**. *Nanoethics* 2009, **3**(3): 217-230. doi: 10.1007/s11569-009-0076-9.
- Spezio ML: **Brain and machine: minding the transhuman future**. *Dialog* 2005, **44**(4). 375-380. doi: 10.1111/j.0012-2033.2005.00281.x.
- Tamburrini G: **Brain to computer communication: ethical perspectives on interaction models**. *Neuroethics* 2009, **2**(3): 137-149. doi: 10.1007/s12152-009-9040-1.
- Vlek RJ et al.: **Ethical issues in brain-computer interface research, development, and dissemination**. *J Neurol Phys Ther* 2012, **36**(2): 94-99. doi: 10.1097/NPT.0b013e31825064cc.
- Wolbring G et al.: **Emerging therapeutic enhancement enabling health technologies and their discourses: what is discussed within the health domain?***Healthcare (Basel)* 2013, **1**(1): 20-52. doi:10.3390/healthcare1010020.
- Wolpe PR: **Ethical and social challenges of brain-computer interfaces**. *Virtual Mentor* 2007, **9**(2): 128-131. doi: 10.1001/virtualmentor.2007.9.2.msoc1-0702.

Books:

- Grübler G, Hildt E: *Brain-Computer Interfaces in Their Ethical, Social and Cultural Contexts*. Dordrecht: Springer; 2014.

Book Chapters:

- Hinterberger T: **Possibilities, limits, and implications of brain-computer interfacing technologies.** In *Scientific and Philosophical Perspectives in Neuroethics*. Edited by James Giordano, Bert Gordijn. Cambridge; Cambridge University Press; 2010: 271-282.
- Johansson V: **Do brain machine interfaces on nano scale pose new ethical challenges?** In *Size Matters: Ethical, Legal and Social Aspects of Nanobiotechnology and Nano-Medicine*. Edited by Johann S. Ach, Christian Weidemann. Münster: LIT Verlag; 2008: 75-99.
- Kennedy P et al.: **Making the lifetime connection between brain and machine for restoring and enhancing function**. In *Brain Machine Interfaces: Implications for Science, Clinical Practice and Society*. Edited by Jens Schouenborg, Martin Garwicz, Nils Danielsen. Bostin: Elsevier Science; 2011:1-25.
- Kleih SC et al.: **Out of the frying pan into the fire – the P300-based BCI faces real-world challenges**. In *Brain Machine Interfaces: Implications for Science, Clinical Practice and Society*. Edited by Jens Schouenborg, Martin Garwicz, Nils Danielsen. Bostin: Elsevier Science; 2011; 27-46.
- Lee KY, Jang D: **Ethical and social issues behind brain-computer interface.** In *2013 International Winter Workshop on Brain-Computer Interface (BCI)*. Piscataway, NJ: IEEE Computer Society; 2013: 72-75.
- McGee EM: **Brain-computer interfaces: ethical and policy considerations**. Implantable Bioelectronics. Edited by Evgeny Katz. Weinheim, Germany: Wiley-VCH; 2014: 411-433.
- McGee EM: **Neuroethics and implanted brain machine interfaces**. In *Uberveillance and the Social Implications of Microchip Implants: Emerging Technologies.* Edited by M.G. Michael, Katina Michael. Hershey; Pennsylvania: Information Science Reference; 2014: 351-365.
- O’Brolchain F, Gordijn B: **Brain-computer interfaces and user responsibility**. In *Brain-Computer Interfaces in Their Ethical, Social and Cultural Contexts*. Edited by Gerd Grübler, Elisabeth Hildt. Dordrecht: Springer; 2014:163-82.
- Rao RPN: **Ethics of brain-computer interfacing**. In his *Brain-Computer Interfacing: An Introduction*. New York: Cambridge University Press; 2013: 272-280.
- Schulze-Bonhage A, Ball T: **Entwicklung und Einsatzmöglichkeiten von Brain-Machine-Interfaces bei Epilepsiepatienten**. In *Das technisierte Gehirn: Neurotechnologien als Herausforderung für Ethik und Anthropologie.* Edited by Oliver Müller, Jens Clausen, Giovanni Maio. Paderborn: Mentis; 2009: 35-49.
- Sententia W: **Neuroethical considerations: cognitive liberty and converging technologies for improving human cognition**. In *The Coevolution of Human Potential and Converging Technologies*. Edited by Mihail C. Roco, Carlo Montemagno. New York: New York Academy of Sciences; 2004: 221-228.
- Thomas AP, Prichard JR: **Brain-machine interfaces: a team-taught seminar bridging disciplines and fostering discussions**. In *2008 IEEE Frontiers in Education Conference*. Piscataway, NJ: IEEE Computer Society; 2008: 337-341.
- Wolpe PR. **Neurotechnology and brain-computer interfaces**. In *Emerging Technologies and Ethical Issues in Engineering: Papers from a Workshop, October 14-15, 2003*. Washington, DC: National Academies Press; 2004: 57-63.

***Neuroprosthetics:***

- Alpert S: **Brain-computer interface devices: risks and Canadian regulations**. *Account Res* 2008, **15**(2):63-86. doi:10.1080/08989620701783774.
- Articulo AC : **Towards an ethics of technology: re-exploring Teilhard de Chardin's theory of technology and evolution**. *Open J Phil* 2014, **4**(4):518-530. doi:10.4236/ojpp.2014.44054.
- Attiah MA, Farah MJ: **Minds and motherboards and money: futurism and realism in the neuroethics of BCI technologies**. *Front Syst Neurosci* 2014, **8**:86. doi:10.3389/fnsys.2014.00086.
- Baertschi B: **Hearing the implant debate: therapy or cultural alienation?***J Int Bioethique* 2013, **24**(4):71-81,181-2.
- Baranauskas G: **What limits the performance of current invasive brain machine interfaces?***Front Syst Neurosci* 2014, **8**:68. doi:10.3389/fnsys.2014.00068.
- Berg AL, Herb A, Hurst M: **Cochlear implants in children: ethics, informed consent, and parental decision making**. *The Journal of Clinical Ethics, 16*(3), 239-250.
- Berg AL, Ip SC, Hurst M, Herb A: **Cochlear implants in young children: informed consent as a process and current practices**. *Am J Audiol* 2007, **16**(1): 13-28. doi: 10.1044/1059-0889(2007/003).
- Bhatt YM et al.: **Device nonuse among adult cochlear implant recipients**. *Otol Neurotol* 2005, **26**(2):183-187.
- Buller T: **Neurotechnology, invasiveness and the extended mind**. *Neuroethics* 2013, **6**(3):593-605. doi:10.1007/s12152-011-9133-5.
- Clark A: **Re-inventing ourselves: the plasticity of embodiment, sensing, and mind**. *J Med Philos* 2007, **32**(3):263-282. doi:10.1080/03605310701397024.
- Clausen J: **Bonding brains to machines: ethical implications of electroceuticals for the human brain**. *Neuroethics* 2013, **6**(3):429-434. doi: 10.1007/s12152-013-9186-8.
- Clausen J: **Conceptual and ethical issues with brain-hardware interfaces**. *Curr Opin Psychiatry* 2011, **24**(6):495-501. doi: 10.1097/YCO.0b013e32834bb8ca.
- Clausen J: **Ethische aspekte von gehirn-computer-schnittstellen in motorischen neuroprothesen [ethical aspects of brain-computer interfacing in neuronal motor prostheses]**. *IRIE* 2006, **5**(9):25-32.
- Clausen J: **Man, machine and in between**. *Nature* 2009, **457**(7233):1080-1081. doi:10.1038/4571080a.
- Clausen J: **Moving minds: ethical aspects of neural motor prostheses**. *Biotechnol J* 2008, **3**(12):1493-1501. doi:10.1002/biot.200800244.
- Decker M, Fleischer T: **Contacting the brain--aspects of a technology assessment of neural implants**. *Biotechnol J* 2008, **3**(12):1502-1510. doi:10.1002/biot.200800225.
- Demetriades AK, Demetriades CK, Watts C, Ashkan K: **Brain-machine interface: the challenge of neuroethics**. *Surgeon* 2010, **8**(5):267-269. doi:10.1016/j.surge.2010.05.006.
- Dielenberg RA: **The speculative neuroscience of the future human brain**. *Humanities* 2013, **2**(2):209-252. doi:10.3390/h2020209.
- Donoghue JP: **Bridging the brain to the world: a perspective on neural interface systems**. *Neuron* 2008, **60**(3):511-521. doi:10.1016/j.neuron.2008.10.037.
- Finlay L, Molano-Fisher P: **'Transforming' self and world: a phenomenological study of a changing lifeworld following a cochlear implant**. *Med Health Care Philos* 2008, **11**(3):255-267. doi:10.1007/s11019-007-9116-9.
- Giselbrecht S, Rapp BE, Niemeyer CM: **The chemistry of cyborgs--interfacing technical devices with organisms**. *Angew Chem Int Ed Engl* 2013, **52**(52):13942-13957. doi:10.1002/anie.201307495.
- Glasser BL: **Supreme Court redefines disability: limiting ADA protections for cochlear implantees in hospitals**. *J Leg Med* 2002, **23**(4):587-608. doi:10.1080/01947640290050355.
- Grau C et al.: **Conscious brain-to-brain communication in humans using non-invasive technologies**. *PloS One* 2014, **9**(8):e105225. doi:10.1371/journal.pone.0105225.
- Grübler G: **Beyond the responsibility gap: discussion note on responsibility and liability in the use of brain-computer interfaces**. *AI Soc* 2011, **26**(4):377-382. doi:10.1007/s00146-011-0321-y.
- Guyot JP, Gay A, Izabel Kos MI, Pelizzone M: **Ethical, anatomical and physiological issues in developing vestibular implants for human use**. *J Vestib Res* 2012, **22**(1):3-9. doi:10.3233/VES-2012-0446.
- Haselager P, Vlek R, Hill J Nijboer F: **A note on ethical aspects of BCI**. *Neural Netw* 2009, **22**(9):1352-1357. doi:10.1016/j.neunet.2009.06.046.
- Hladek GA: **Cochlear implants, the deaf culture, and ethics: a study of disability, informed surrogate consent, and ethnocide**. *Monash Bioeth Rev* 2002, **21**(1):29-44.
- Hoag H: **Remote control**. *Nature* 2003, **423**(6942):796-798. doi:10.1038/423796a.
- Huggins JE, Wolpaw JR: **Papers from the Fifth International Brain-Computer Interface Meeting: preface**. *J Neural Eng* 2014, **11**(3): 030301. doi:10.1088/1741-2560/11/3/030301.
- Hyde M, Power D: **Some ethical dimensions of cochlear implantation for deaf children and their families**. *J Deaf Stud Deaf Educ* 2006, **11**(1):102-111. doi: 10.1093/deafed/enj009.
- Jain N: **Brain-machine interface: the future is now**. *Natl Med J India* 2010, **23**(6):321-323.
- Jebari K, Hansson SO: **European public deliberation on brain machine interface technology: five convergence seminars**. *Sci Eng Ethics* 2013, **19**(3): 1071-1086. doi:10.1007/s11948-012-9425-0.
- Kermit P: **Enhancement technology and outcomes: what professionals and researchers can learn from those skeptical about cochlear implants**. *Health Care Anal* 2012, **20**(4):367-384. doi:10.1007/s10728-012-0225-0.
- Kotchetkov IS et al.: **Brain-computer interfaces: military, neurosurgical, and ethical perspective**. *Neurosurg Focus* 2010, **28**(5):E25. doi:10.3171/2010.2.FOCUS1027.
- Kübler A, Mushahwar VK, Hochberg LR, Donoghue JP: **BCI Meeting 2005--workshop on clinical issues and applications**. *IEEE Trans Neural Syst Rehabil Eng* 2006, **14**(2):131-134. doi:10.1109/TNSRE.2006.875585.
- Laryionava K, Gross D: **Public understanding of neural prosthetics in Germany: ethical, social, and cultural challenges**. *Camb Q Healthc Ethics* 2011, **20**(3):434-439. doi:10.1017/S0963180111000119.
- Levy N: **Reconsidering cochlear implants: the lessons of Martha's Vineyard**. *Bioethics* 2002, **16**(2):134-153. doi:10.1111/1467-8519.00275.
- Lucas MS: **Baby steps to superintelligence: neuroprosthetics and children**. *J Evol Technol* 2012, **22**(1):132-145.
- Lucivero F, Tamburrini G: **Ethical monitoring of brain-machine interfaces**. *AI & Soc* 2008, **22**(3):449-460. doi:10.1007/s00146-007-0146-x.
- McCullagh P, Lightbody G, Zygierewicz J: **Ethical challenges associated with the development and deployment of brain computer interface technology**. Neuroethics 2014, **7**(2):109-122. doi:10.1007/s12152-013-9188-6.
- McGee EM, Maguire GQ Jr.: **Becoming borg to become immortal: regulating brain implant technologies**. *Camb Q Healthc Ethics* 2007, **16**(3):291-302. doi:10.1017/S0963180107070326.
- McGie S, Nagai M, Artinian-Shaheen T: **Clinical ethical concerns in the implantation of brain-machine interfaces: part I: overview, target populations, and alternatives**. *IEEE Pulse* 2013, **4**(1):28-32. doi:10.1109/MPUL.2012.2228810.
- McGie SC, Nagai MK, Artinian-Shaheen T: **Clinical ethical concerns in the implantation of brain-machine interfaces**. *IEEE Pulse* 2013, **4**(2):32-37. doi:10.1109/MPUL.2013.2242014.
- Melton MF, Backous DD: **Preventing complications in pediatric cochlear implantation**. *Curr Opin Otolaryngol Head Neck Surg* 2011, **19**(5):358-362. doi:10.1097/MOO.0b013e32834a023b.
- Mizushima N, Sakura O: **Project-based approach to identify the ethical, legal and social implications: a model for national project of brain machine interface development**. *Neurosci Res* 2011, **71**(Suppl 1):e392. doi:10.1016/neures.2011.07.1715.
- Mizushima N, Isobe T, Sakura O: **Neuroethics at the benchside: a preliminary report of research ethics consultation in BMI studies**. *Neurosci Res* 2009, **65**(S1):S134. doi:10.1016/neures.2009.09.657.
- Nijboer F, Clausen J, Allison BZ, Haselager P: **The Asilomar Survey: stakeholders' opinions on ethical issues related to brain-computer interfacing**. *Neuroethics* 2013, **6**(3):541-578. doi: 10.1007/s12152-011-9132-6.
- Nijboer F: **Ethical, legal and social approach, concerning BCI applied to LIS patients**. *Ann Phys Rehabil Med* 2014, **57**(Supp 1):e244. doi:10.1016/j.rehab.2014.03.1145.
- Nikolopoulos, T. P., Dyar, D., & Gibbin, K. P. (2004). **Assessing candidate children for cochlear implantation with the Nottingham Children's Implant Profile (NChIP): the first 200 children**. *Int J Pediatr Otorhinolaryngol* 2004, **68**(2):127-135.
- Peterson GR: **Imaging god: cyborgs, brain-machine interfaces, and a more human future**. *Dialog J Theol* 2005, **44**(4):337-346. doi:10.1111/j.0012-2033.2005.00277.x.
- Poppendieck W et al.: **Ethical issues in the development of a vestibular prosthesis**. *Conf Proc IEEE Eng Med Biol Soc* 2011, **2011**:2265-2268. doi:10.1109/IEMBS.2011.6090570.
- Purcell-Davis A: **The representations of novel neurotechnologies in social media: five case studies**. *New Bioeth* 2013, **19**(1):30-45. doi:10.1179/2050287713Z.00000000026.
- Racine E et al.: **"Currents of hope": neurostimulation techniques in U.S. and U.K. print media**. *Camb Q Healthc Ethics* 2007, **16**(3):312-316. doi:10.1017/S0963180107070351.
- Rowland NC, Breshears J, Chang EF: **Neurosurgery and the dawning age of brain-machine interfaces**. *Surg Neurol Int* 2013, **4**(2):S11-S14. doi:10.4103/2152-7806.109182.
- Ryu SI, Shenoy KV: **Human cortical prostheses: lost in translation?***Neurosurg Focus* 2009, **27**(1):E5. doi:10.3171/2009.4.FOCUS0987.
- Saha S, Chhatbar P: **The future of implantable neuroprosthetic devices: ethical considerations**. *J Long Term Eff Med Implants* 2009, **19**(2):123-137. doi:10.1615/JLongTermEffMedImplants.v19.i2.40.
- Sakura O: **Brain-machine interface and society: designing a system of ethics and governance**. *Neurosci Res* 2009, **65**(S1):S33. doi:10.1016/j.neures.2009.09.1687.
- Sakura O: **Toward making a regulation of BMI**. *Neurosci Res* 2011, 71(Suppl):e9. doi:10.1016/j.neures.2011.07.030.
- Santos Santos S: **Aspectos bioeticos en implantes cocleares pediatricos [Bioethical issues in pediatric cochlear implants]**. *Acta Otorrinolaringol Esp* 2002, **53**(8):547-558.
- Schermer M: **The mind and the machine: on the conceptual and moral implications of brain-machine interaction**. *Nanoethics* 2009, **3**(3):217-230. doi:10.1007/s11569-009-0076-9.
- Spezio ML: **Brain and machine: minding the transhuman future**. *Dialog J Theol* 2005, **44**(4):375-380. doi:10.1111/j.0012-2033.2005.00281.x.
- Tamburrini G: **Brain to computer communication: ethical perspectives on interaction models**. *Neuroethics* 2009, **2**(3):137-149. doi: 10.1007/s12152-009-9040-1.
- Taylor F, Hine C: **Cochlear implants: informing commissioning decisions, based on need**. *J Laryngol Otol* 2006, **120**(12):1008-1013. doi: 10.1017/S0022215106003392.
- Thébaut C: **Dealing with moral dilemma raised by adaptive preferences in health technology assessment: the example of growth hormones and bilateral cochlear implants**. *Soc Sci Med* 2013, **99**:102-109. doi:10.1016/j.socscimed.2013.10.020.
- Vlek RJ et al.: **Ethical issues in brain-computer interface research, development, and dissemination**. *J Neurol Phys Ther* 2012, **36**(2):94-99. doi: 10.1097/NPT.0b013e31825064cc.

Books:

- Berger TW: *Toward Replacement Parts for the Brain: Implantable Biomimetic Electronics as Neural Prostheses*. Cambridge, Mass.: MIT Press; 2005.
- Christiansen JB, Leigh IW: *Cochlear Implants in Children: Ethics and Choices*. Washington, D.C.: Gallaudet University Press; 2002.
- Grübler G, Hildt E: *Brain-Computer-Interfaces in their Ethical, Social and Cultural Contexts*. Dordrecht: Springer; 2014.
- Luppicini R: *Handbook of Research on Technoself: Identity in a Technological Society*. Hershey, Pa.: Information Science Reference; 2013.

Book chapters:

- Chase VD: **Ethics**. In *his Shattered Nerves: How Science is Solving Modern Medicine's Most Perplexing Problem*. Baltimore, MD: Johns Hopkins University Press; 2006:251-272.
- Clark G: **Socioeconomics and ethics**. In *his Cochlear Implants: Fundamentals and Applications*. New York: Springer; 2003:767-786.
- McGee EM: **Brain-computer interfaces: ethical and policy considerations**. In *Implantable Bioelectronics*. Edited by Evgeny Katz. Weinheim, Germany: Wiley-VCH; 2014:411-433.

***Neural stem cells and neural tissue transplantation:***

- Albin RL: **Sham surgery controls: intracerebral grafting of fetal tissue for Parkinson's disease and proposed criteria for use of sham surgery controls**. *J Med Ethics* 2002, **28**(5):322-325. doi:10.1136/jme.28.5.322.
- American Academy of Neurology, American Neurological Association: **Position statement regarding the use of embryonic and adult human stem cells in biomedical research**. *Neurology* 2005, **64**(10):1679-1680. doi: 10.1212/01.WNL.0000161879.09113.DE.
- Anderson DK: **Neural tissue transplantation in Syringomyelia: feasibility and safety.***Ann N Y Acad Sci* 2002, **961**:263-264. doi:10.1111/j.1749-6632.2002.tb03097.x.
- Anisimov SV: **[Cell therapy for Parkinson's disease: IV. risks and future trends.]***Adv Gerontol* 2009, **22**(3):418-439.
- Arias-Carrión O, Yuan TF: **Autologous neural stem cell transplantation: a new treatment option for Parkinson's disease?***Med Hypotheses* 2009, **73**(5):757-759. doi:10.1016/j.mehy.2009.04.029.
- Baertschi B: **Intended changes are not always good, and unintended changes are not always bad--why?***Am J Bioeth* 2009, **9**(5):39-40. doi:10.1080/15265160902788710.
- Barker RA: **Neural transplants for Parkinson’s disease: what are the issues?***Poiesis Prax* 2006, **4**(2):129-143. doi:10.1007/s10202-006-0021-8.
- Barker RA, de Beaufort I: **Scientific and ethical issues related to stem cell research and interventions in neurodegenerative disorders of the brain**. *Prog Neurobiol* 2013, **110**:63-73. doi:10.1016/j.pneurobio.2013.04.003.
- Bell E et al.: **Responding to requests of families for unproven interventions in neurodevelopmental disorders: hyperbaric oxygen "treatment" and stem cell "therapy" in cerebral palsy**. *Dev Disabil Res Rev* 2011, **17**(1):19-26. doi:10.1002/ddrr.134.
- Benes FM: **Deserving the last great gift**. *Cerebrum* 2003, **5**(3):61-73.
- Benninghoff J, Möller HJ, Hampel H, Vescovi AL: **The problem of being a paradigm: the emergence of neural stem cells as example for "Kuhnian" revolution in biology or misconception of the scientific community?***Poiesis Prax* 2009, **6**(1):3-11. doi: 10.1007/s10202-008-0056-0.
- Bjarkam CR, Sørensen JC: **Therapeutic strategies for neurodegenerative disorders: emerging clues from Parkinson's disease.***Biol Psychiatry* 2004, **56**(4):213-216. doi:10.1016/j.biopsych.2003.12.025.
- Boer GJ, Widner H: **Clinical neurotransplantation: core assessment protocol rather than sham surgery as control**. *Brain Res Bull* 2002, **58**(6):547-553. doi:10.1016/S0361-9230(02)00804-3.
- Bojar M: **Pohled neurologa na bunecnou a genovou lecbu chorob nervoveho system [A neurologist's views on cellular and gene therapy in nervous system diseases].***Cas Lek Cesk* 2003, 142(9):534-537.
- Carter A, Bartlett P, Hall W: **Scare-mongering and the anticipatory ethics of experimental technologies.***Am J Bioeth* 2009, **9**(5):47-48. doi:10.1080/15265160902788736.
- Chandran S: **What are the prospects of stem cell therapy for neurology?***BMJ* 2008, **337**:a1934. doi:10.1136/bmj.a1934.
- Cheshire WP: **Miniature human brains: an ethical analysis**. *Ethics Med* 2014, **30**(1):7-12.
- Coors ME: **Considering chimeras: the confluence of genetic engineering and ethics**. *Natl Cathol Bioeth Q* 2006, **6**(1):75-87. doi:10.5840/ncbq20066168.
- de Amorim AR: **Regulating ethical issues in cell-based interventions: lessons from universal declaration on bioethics and human rights**. *Am J Bioeth* 2009, **9**(5):49-50. doi:10.1080/15265160902807361.
- Drouin-Ouellet J: **The potential of alternate sources of cells for neural grafting in Parkinson's and Huntington's disease**. *Neurodegener Dis Manag* 2014, **4**(4):297-307. doi:10.2217/nmt.14.26.
- Duggan PS et al.: **Unintended changes in cognition, mood, and behavior arising from cell-based interventions for neurological conditions: ethical challenges**. *Am J Bioeth* 2009, **9**(5):31-36. doi:10.1080/15265160902788645.
- Dunnett SB, Rosser AE: **Cell transplantation for Huntington's disease: should we continue?***Brain Res Bull* 2007, **72**(2-3):132-147. doi:10.1016/j.brainresbull.2006.10.019.
- Dunnett SB, Rosser AE: **Challenges for taking primary and stem cells into clinical neurotransplantation trials for neurodegenerative disease**. *Neurobiol Dis* 2014, **61**:79-89. doi:10.1016/j.nbd.2013.05.004.
- Feldmann RE Jr., Mattern R: **The human brain and its neural stem cells postmortem: from dead brains to live therapy**. *Int J Legal Med* 2006, **120**(4):201-211. doi:10.1007/s00414-005-0037-y.
- Fisher MMJ: **The BAC consultation on neuroscience and ethics an anthropologist's perspective**. *Innovation* 2013, **12**(1):40-43.
- Gasparini M et al.: **Stem cells and neurology: cues for ethical reflections**. *Neurol Sci* 2004, **25**(2):108-113. doi:10.1007/s10072-004-0241-4.
- Gazzaniga MS: **The thoughtful distinction between embryo and human**. *Chron High Educ* 2005, **51**(31):B10-B12.
- Gazzaniga MS: **What's on your mind?***New Sci* 2005, 186(2503):48-50.
- Giordano J: **Neuroethical issues in neurogenetic and neuro-implantation technology: the need for pragmatism and preparedness in practice and policy.***Stud Ethics Law and Technol* 2011, **4**(3). doi:10.2202/1941-6008.1152.
- Goldman SA: **Neurology and the stem cell debate.***Neurology* 2005, **64**(10):1675-1676. doi:10.1212/01.WNL.0000165312.12463.BE.
- Grisolia JS: **CNS stem cell transplantation: clinical and ethical perspectives**. *Brain Res Bull* 2002, **57**(6):823-826. doi:10.1016/S0361-9230(01)00766-3.
- Grunwell J, Illes J, Karkazis K: **Advancing neuroregenerative medicine: a call for expanded collaboration between scientists and ethicists**. *Neuroethics* 2009, **2**:13-20. doi:10.1007/s12152-008-9025-5.
- Harrower TP, Barker RA: **Is there a future for neural transplantation?***Biodrugs* 2004, **18**(3):141-153. doi:10.2165/00063030-200418030-00001.
- Hermerén G: **Ethical challenges for using human cells in clinical cell therapy**. *Prog Brain Res* 2012, **200**:17-40. doi:10.1016/B978-0-444-59575-1.00002-8.
- Hess PG: **Risk of tumorigenesis in first-in-human trials of embryonic stem cell neural derivatives: ethics in the face of long-term uncertainty**. *Account Res* 2009, **16**(4):175-198. doi:10.1080/08989620903065145.
- Hildt E: **Ethical challenges in cell-based interventions for neurological conditions: some lessons to be learnt from clinical transplantation trials in patients with Parkinson's disease**. *Am J Bioeth* 2009, **9**(5):37-38. doi:10.1080/15265160902850999.
- Hug K, Hermerén G: **Differences between Parkinson’s and Huntington’s diseases and their role for prioritization of stem cell-based treatments.** I 2013, **13**(5):777-791. doi: 10.2174/1566524011313050009.
- Illes J, Reimer JC, Kwon BK. **Stem cell clinical trials for spinal cord injury: readiness, reluctance, redefinition**. *Stem Cell Rev* 2011, **7**(4):997-1005. doi:10.1007/s12015-011-9259-1.
- Kaneko N, Kako E, Sawamoto K: **Prospects and limitations of using endogenous neural stem cells for brain regeneration.***Genes (Basel)* 2011, **2**(1):107-130. doi:10.3390/genes2010107.
- Kempermann G: **Neuronal stem cells and adult neurogenesis.***Ernst Schering Res Found Workshop* 2002, (35):17-28.
- Korean Movement Disorders Society Red Tulip Survey Participants et al.: **Nationwide survey of patient knowledge and attitudes towards human experimentation using stem cells or bee venom acupuncture for Parkinson's disease.***J Mov Disord* 2014, **7**(2):84-91. doi:10.14802/jmd.14012.
- Kosta E, Bowman DM: **Treating or tracking? regulatory challenges of nano-enabled ICT implants**. *Law Policy* 2011, **33**(2):256-275. doi:10.1111/j.1467-9930.2010.00338.x.
- Laguna Goya R, Kuan WL, Barker RA: **The future of cell therapies in the treatment of Parkinson's disease**. *Expert Opin Biol Ther* 2007, **7**(10):1487-1498. doi:10.1517/14712598.7.10.1487.
- Lo B, Parham L: **Resolving ethical issues in stem cell clinical trials: the example of Parkinson disease**. *J Law Med Ethics* 2010, **38**(2):257-266. doi:10.1111/j.1748-720X.2010.00486.x.
- Lopes M, Meningaud JP, Behin A, Hervé C: **Consent: a Cartesian ideal? human neural transplantation in Parkinson's disease.***Med Law* 2003, 22(1):63-71.
- Master Z, McLeod M, Mendez I: **Benefits, risks and ethical considerations in translation of stem cell research to clinical applications in Parkinson's disease**. *J Med Ethics* 2007, **33**(3):169-173. doi: 10.1136/jme.2005.013169.
- Martino G et al.: **Stem cell transplantation in multiple sclerosis: current status and future prospects**. *Nat Rev Neurol* 2010, **6**(5):247-255. doi:10.1038/nrneurol.2010.35.
- Mathews DJ et al.: **Cell-based interventions for neurologic conditions: ethical challenges for early human trials**. *Neurology* 2008, **71**(4):288-293. doi:10.1212/01.wnl.0000316436.13659.80.
- Medina JJ: **Custom-made neural stem cells**. *Psychiatr Times* 2011, **28**(4):41-42.
- Moreira T, Palladino P: **Between truth and hope: on Parkinson’s disease, neurotransplantation and the production of the ‘self’**. *Hist Human Sci* 2005, **18**(3):55-82. doi:10.1177/0952695105059306.
- Norman TR: **Human embryonic stem cells: A resource for in vitro neuroscience research?***Neuropsychopharmacology* 2006, **31**(12):2571-2572. doi:10.1038/sj.npp.1301126.
- Olson SF: **American Academy of Neurology development of a position on stem cell research.***Neurology* 2005, **64**(10):1674. doi:10.1212/01.WNL.0000165657.74376.EF.
- Pandya SK: **Medical ethics in the neurosciences**. *Neurol India* 2003, **51**(3):317-322.
- Parke S, Illes J: **In delicate balance: stem cells and spinal cord injury advocacy.***Stem Cell Rev* 2011, **7**(3):657-663. doi:10.1007/s12015-010-9211-9.
- Pendleton C, Ahmed I, Quinones-Hinojosa A: **Neurotransplantation: lux et veritas, fiction or reality?***J Neurosurg Sci* 2011, **55**(4):297-304.
- Pullicino PM, Burke WJ: **Cell-based interventions for neurologic conditions: ethical challenges for early human trials**. *Neurology* 2009, **72**(19):1709. doi:10.1212/01.wnl.0000346753.90198.a6.
- Ramos-Zúñiga R et al.: **Ethical implications in the use of embryonic and adult neural stem cells**. *Stem Cells Int* 2012, **2012**:470949. doi:10.1155/2012/470949.
- Reiner PB: **Unintended benefits arising from cell-based interventions for neurological conditions**. *Am J Bioeth* 2009, **9**(5):51-52. doi:10.1080/15265160902788769.
- Romano G: **Stem cell transplantation therapy: controversy over ethical issues and clinical relevance.***Drug News Perspect* 2004, **17**(10):637-645.
- Rosenberg RN, World Federation of Neurology: **World Federation of Neurology position paper on human stem cell research.***J Neurol Sci* 2006, **243**(1-2):1-2. doi:10.1016/j.jns.2006.02.001.
- Rosenfeld JV, Bandopadhayay P, Goldschlager T, Brown DJ: **The ethics of the treatment of spinal cord injury: stem cell transplants, motor neuroprosthetics, and social equity**. *Top Spinal Cord Inj Rehabil* 2008, **14**(1):76-88. doi:10.1310/sci1401-76.
- Rosser AE, Kelly CM, Dunnett SB: **Cell transplantation for Huntington's disease: practical and clinical considerations.***Future Neurol* 2011, **6**(1):45-62. doi:10.2217/fnl.10.78.
- Rothstein JD, Snyder EY: **Reality and immortality--neural stem cells for therapies.***Nat Biotechnol* 2004, **22**(3):283-285. doi:10.1038/nbt0304-283.
- Samarasekera N et al.: **Brain banking for neurological disorders.***Lancet Neurol* 2013, **12**(11):1096-1105. doi:10.1016/S1474-4422(13)70202-3.
- Sanberg PR: **Neural stem cells for Parkinson's disease: to protect and repair.***Proc Natl Acad Sci U S A* 2007, **104**(29):11869-11870. doi:10.1073/pnas.0704704104.
- Sayles M, Jain M, Barker RA: **The cellular repair of the brain in Parkinson's disease--past, present and future**. *Transpl Immunol* 2004, **12**(3-4):321-342. doi:10.1016/j.trim.2003.12.012.
- Schanker BD: **Inevitable challenges in establishing a causal relationship between cell-based interventions for neurological conditions and neuropsychological changes**. *Am J Bioeth* 2009, **9**(5):43-45. doi:10.1080/15265160902788686.
- Schermer M: **Changes in the self: the need for conceptual research next to empirical research**. *Am J Bioeth* 2009, **9**(5):45-47. doi:10.1080/15265160902788744.
- Schwartz PH, Kalichman MW: **Ethical challenges to cell-based interventions for the central nervous system: some recommendations for clinical trials and practice**. *Am J Bioeth* 2009, **9**(5):41-43. doi:10.1080/15265160902788694.
- Silani V, Cova L: **Stem cell transplantation in multiple sclerosis: safety and ethics.***J Neurol Sci* 2008, **265**(1-2), 116-121. doi:10.1016/j.jns.2007.06.010.
- Silani V, Leigh N: **Stem therapy for ALS: hope and reality**. *Amyotroph Lateral Scler Other Motor Neuron Disord* 2003, **4**(1):8-10. doi:10.1080/1466082031006652.
- Sivarajah N: **Neuroregenerative gene therapy: the implications for informed consent laws**. *Health Law Can* 2005, **26**(2):19-28.
- Takahashi R, Kondo T: **[Cell therapy for brain diseases: perspective and future prospects]**. *Rinsho Shinkeiqaku* 2011, **51**(11):1075-1077.
- Tandon PN: **Transplantation and stem cell research in neurosciences: where does India stand?***Neurol India* 2009, **57**(6):706-714. doi:10.4103/0028-3886.59464.
- Takala T, Buller T: **Neural grafting: implications for personal identity and personality.***Trames* 2011, **15**(2):168-178. doi:10.3176/tr.2011.2.05.
- Wang L, Lu M: **Regulation and direction of umbilical cord blood mesenchymal stem cells to adopt neuronal fate**. *Int J Neurosci* 2014, **124**(3):149-159. doi:10.3109/00207454.2013.828055.
- Wang Y: **Chinese views on the ethical issues and governance of stem cell research.***Eubios J Asian Int Bioeth* 2014, 24(3):87-93.

Books:

- Fangerau H, Fegert J, Trapp T: *Implanted Minds: The Neuroethics of Intracerebral Stem Cell Transplantation and Deep Brain Stimulation*. Bielefeld, Germany: Transcript Verlag; 2011.
- Ford NM, Herbert M: *Stem Cells: Science, Medicine, Law and Ethics*. Strathfield, NSW, Australia: St. Pauls; 2003.

Book chapters:

- Boer GJ: **Transplantation and xenotransplantation**. In *Scientific and Philosophical Perspectives in Neuroethics*. Edited by James J. Giordano, Bert Gordijn. Cambridge, UK: Cambridge University Press; 2010:190-215.
- Bührle CP: **Changes in personality: possible hazards arising from stem cell graft--an ethical and philosophical approach**. In *Implanted Minds: The Neuroethics of Intracerebral Stem Cell Transplantation and Deep Brain Stimulation*. Edited by Heiner Fangerau, Jörg M. Fegert, Thorsten Trapp. Bielefeld, Germany: Transcript Verlag; 2011:57-90.
- Dunnett SB, Borlongan CV, Sanberg PR: **Embryonic or neural stem cells in neurodegenerative disease of the central nervous system (with relevance to PD, HD, AD, MS, SCI, and stroke)**. In *Tissue and Cell Use: An Essential Guide.* Edited by Ruth M. Warwick, Scott A. Brubaker. Chichester, West Sussex, U.K.: Wiley-Blackwell; 2012:358-382.
- Goldstein J: **Parfit's concept of personal identity and its implications for intercerebral stem cell transplants**. In *Implanted Minds: The Neuroethics of Intracerebral Stem Cell Transplantation and Deep Brain Stimulation*. Edited by Heiner Fangerau, Jörg M. Fegert, Thorsten Trapp. Bielefeld, Germany: Transcript Verlag; 2011:45-56.
- Green RM: **Ethical considerations**. In *Principles of Regenerative Medicine*, *2*^*nd*^*edition*. Edited by Anthony Atala, Robert Lanza, James A. Thomson and Robert M. Nerem. London: Academic Press; 2011:1117-1130.
- Hook L, Fulton N, Russell G, Allsopp T: **Human neural stem cells for biopharmaceutical applications**. In *Stem Cell Research and Therapeutics*. Edited by Yanhong Shi, Dennis Owen Clegg. Dordrecht: springer; 2008:123-140.
- Macklin R: **Ethics and stem cell research**. In *Stroke Recovery with Cellular Therapies*. Edited by Sean I. Savitz, Daniel M. Rosenbaum. New York, NY: Humana Press; 2008:133-149.
- Mauron A, Hurst S: **Experimenting with innovative cell therapies for Parkinson's disease: a view from ethics**. In *Implanted Minds: The Neuroethics of Intracerebral Stem Cell Transplantation and Deep Brain Stimulation*. Edited by Heiner Fangerau, Jörg M. Fegert, Thorsten Trapp. Bielefeld, Germany: Transcript Verlag; 2011:107-122.
- Nuffield Council on Bioethics: **Neural stem cell therapies**. In *Novel Neurotechnologies: Intervening in the Brain*. London: Nuffield Council on Bioethics; 2013:36-39.

***Issues Concerning Pediatric Subjects/Patients:***

- Altavilla A et al.: **Activity of ethics committees in Europe on issues related to clinical trials in paediatrics: results of a survey**. *Pharmaceuticals Policy & Law* 2009, **11**(1/2): 79-87. doi: 10.3233/PPL-2009-0208.
- Ball N, Wolbring G: **Cognitive enhancement: perceptions among parents of children with disabilities**. *Neuroethics* 2014, **7**(3): 345-364. doi: 10.1007/s12152-014-9201-8.
- Battles HT, Manderson L: **The Ashley Treatment: furthering the anthropology of/on disability**. *Med Anthropol* 2008, **27**(3): 219-26. doi: 10.1080/01459740802222690.
- Bell E et al.: **Responding to requests of families for unproven interventions in neurodevelopmental disorders: hyperbaric oxygen ‘treatment’ and stem cell ‘therapy’ in cerebral palsy**. *Dev Disabil Res Rev* 2011, **17**(1): 19-26. doi: 10.1002/ddrr.134.
- Borgelt EL, Buchman DZ, Weiss M, Illes J: **In search of “anything that would help”: parent perspectives on emerging neurotechnologies**. *J Atten Disord* 2014, **18**(5): 395-401. doi: 10.1177/1087054712445781.
- Brody GH et al.: **Using genetically informed, randomized prevention trials to test etiological hypotheses about child and adolescent drug use and psychopathology.***Am J Public Health* 2013, **103**(S1): S19-S24. doi: 10.2105/AJPH.2012.301080.
- Caplan A: **Accepting a helping hand can be the right thing to do***. J Med Ethics* 2013, **39**(6): 367-368. doi: 10.1136/medethics-2012-100879.
- Clausen J: **Ethical brain stimulation – neuroethics of deep brain stimulation in research and clinical practice**. *Eur J Neurosci* 2010, **32**(7): 1152-1162. doi: 10.1111/j.1460-9568.2010.07421.x.
- Coch D: **Neuroimaging research with children: ethical issues and case scenarios**. *J Moral Educ* 2007, **36**(1): 1-18. doi: 10.1080/03057240601185430.
- Cohen Kadosh K, Linden DE, Lau JY: **Plasticity during childhood and adolescence: innovative approaches to investigating neurocognitive development**. *Dev Sci* 2013, **16**(4): 574-583. doi: 10.1111/desc.12054.
- Cole CM, et al.: **Ethical dilemmas in pediatric and adolescent psychogenic nonepileptic seizures**. *Epilepsy Behav* 2014, **37**: 145-150. doi: 10.1016/j.yebeh.2014.06.019.
- Connors CM, Singh I: **What we should really worry about in pediatric functional magnetic resonance imaging (fMRI)**. *AJOB* 2009, **9**(1): 16-18. doi: 10.1080/15265160802617944.
- Cornfield DN, Kahn JP: **Decisions about life-sustaining measures in children: in whose best interests?***Acta Paediatr* 2012, **101**(4): 333-336. doi: 10.1111/j.1651-2227.2011.02531.x.
- Croarkin PE, Wall CA, Lee J: **Applications of transcranial magnetic stimulation (TMS) in child and adolescent psychiatry**. *Int Rev Psychiatry* 2011, **23**(5): 445-453. doi: 10.3109/09540261.2011.623688.
- Davis NJ: **Transcranial stimulation of the developing brain: a plea for extreme caution.***Front Hum Neurosci* 2014, **8**:600. doi: 10.3389/fnhum.2014.00600.
- Denne SC: **Pediatric clinical trial registration and trial results: an urgent need for improvement**. *Pediatrics* 2012, **129**(5):e1320-1321. doi: 10.1542/peds.2012-0621.
- Derivan AT et al.: **The ethical use of placebo in clinical trials involving children**. *J Child Adolesc Psychopharmacol*2004, **14**(2): 169-174. doi:10.1089/1044546041649057.
- DeVeaugh-Geiss J et al.: **Child and adolescent psychopharmacology in the new millennium: a workshop for academia, industry, and government**. *J Am Acad Child Adolesc Psychiatry* 2006, **45**(3): 261-270. doi:10.1097/01.chi.0000194568.70912.ee.
- Di Pietro NC, Illes J: **Disclosing incidental findings in brain research: the rights of minors in decision-making.***J Magn Reson Imaging* 2013, **38**(5): 1009-1013. doi: 10.1002/jmri.24230.
- Downie J, Marshall J: **Pediatric neuroimaging ethics**. *Camb Q Healthc Ethics* 2007, **16**(2): 147-160. doi: 10.1017/S096318010707017X .
- Elger BS, Harding TW: **Should children and adolescents be tested for Huntington’s Disease? attitudes of future lawyers and physicians in Switzerland**. *Bioethics* 2006, **20**(3): 158-167. doi: 10.1111/j.1467-8519.2006.00489.x.
- Fenton A, Meynell L, Baylis F: **Ethical challenges and interpretive difficulties with non-clinical applications of pediatric FMRI.***Am J Bioeth* 2009, **9**(1): 3-13. doi: 10.1080/15265160802617829.
- Focquaert F: **Deep brain stimulation in children: parental authority versus shared decision-making**. *Neuroethics* 2013, **6**(3): 447-455. doi: 10.1007/s12152-011-9098-4.
- Gilbert DL et al.: **Should transcranial magnetic stimulation research in children be considered minimal risk?***Clin Neurophysiol* 2004, **115**(8): 1730-1739. doi:10.1016/j.clinph.2003.10.037.
- Greenhill LL et al.: **Developing methodologies for monitoring long-term safety on psychotropic medications in children: report on the NIMH Conference, September 25, 2000**. *J Am Acad Child Adolesc Psychiatry* 2003, **42**(6): 651-655. doi: 10.1097/01.CHI.0000046842.56865.EC.
- Hardiman M, Rinne L, Gregory E, Yarmolinskaya J: **Neuroethics, neuroeducation, and classroom teaching: where the brain sciences meet pedagogy**. *Neuroethics* 2012, **5**(2): 135-143. doi: 10.1007/s12152-011-9116-6.
- Hinton VJ: **Ethics of neuroimaging in pediatric development**. *Brain Cogn* 2002, **50**(3): 455-468. doi: 10.1016/S0278-2626(02)00521-3.
- Hyman SE: **Might stimulant drugs support moral agency in ADHD children?***J Med Ethics* 2013, **39**(6): 369-370. doi: 10.1136/medethics-2012-100846.
- Illes J, Raffin TA: **No child left without a brain scan? toward a pediatric neuroethics.***Cerebrum* 2005, **7**(3): 33-46.
- Kadosh RC et al.: **The neuroethics of non-invasive brain stimulation.***Curr Bio* 2012, **22**(4): R108-R111. doi: 10.1016/j.cub.2012.01.013.
- Koelch M, Schnoor K, Fegert JM: **Ethical issues in psychopharmacology of children and adolescents**. *Curr Opin Psychiatry* 2008, **21**(6): 598-605. doi:10.1097/YCO.0b013e328314b776. 
- Kölch M et al.: **Safeguarding children's rights in psychopharmacological research: ethical and legal issues**. *Curr Pharm Des* 2010, **16**(22): 2398-2406. doi: 10.2174/138161210791959881.
- Kumra S et al.: **Ethical and practical considerations in the management of incidental findings in pediatric MRI studies**. *J Am Acad Child Adolesc* 2006, **45**(8): 1000-1006. doi: 10.1097/01.chi.0000222786.49477.a8.
- Ladd RE: **Rights of the autistic child**. *Int'l J Child Rts* 2005, **13**(1/2): 87-98. doi: 10.1163/1571818054545303.
- Lantos JD: **Dangerous and expensive screening and treatment for rare childhood diseases: the case of Krabbe disease**. *Dev Disabil Res Rev* 2011. **17**(1): 15-18. doi: 10.1002/ddrr.133.
- Lantos JD: **Ethics for the pediatrician: the evolving ethics of cochlear implants in children**. *Pediatr Rev* 2012, **33**(7):323-326. doi:10.1542/pir.33-7-323.
- Larivière-Bastien D, Racine E: **Ethics in health care services for young persons with neurodevelopmental disabilities: a focus on cerebral palsy**. *J Child Neurol* 2011, **26**(10): 1221-1229. doi: 10.1177/0883073811402074.
- Lefaivre MJ, Chambers CT, Fernandez CV: **Offering parents individualized feedback on the results of psychological testing conducted for research purposes with children: ethical issues and recommendations**. *J Clin Child Adolesc Psychol* 2007, **36**(2): 242-252. doi: 10.1080/15374410701279636.
- Lev O, Wilfond BS, McBride CM: **Enhancing children against unhealthy behaviors—an ethical and policy assessment of using a nicotine vaccine**. *Public Health Ethics* 2013, **6**(2): 197-206. doi: 10.1093/phe/pht006.
- Lucas MS: **Baby steps to superintelligence: neuroprosthetics and children**. *J Evol Technol* 2012, **22**(1):132-145.
- Martin A, Gilliam WS, Bostic JQ, Rey JM: **Child psychopharmacology, effect sizes, and the big bang**. *Am J Psychiatry* 2005, **162**(4): 817. doi: 10.1176/appi.ajp.162.4.817-a.
- Maslen H, Earp BD, Cohen Kadosh R, Savulescu J: **Brain stimulation for treatment and enhancement in children: an ethical analysis**. *Front Hum Neurosci* 2014, **8**: 953. doi: 10.3389/fnhum.2014.00953
- Maxwell B, Racine E: **Does the neuroscience research on early stress justify responsive childcare? examining interwoven epistemological and ethical challenges**. *Neuroethics* 2012, **5**(2): 159-172. doi: 10.1007/s12152-011-9110-z.
- Miziara ID, Miziara CS, Tsuji RK, Bento RF: **Bioethics and medical/legal considerations on cochlear implants in children**. *Braz J Otorhinolaryngol* 2012, **78**(3):70-79. doi:10.1590/S1808-86942012000300013.
- Moran FC et al.: **Effect of home mechanical in-exsufflation on hospitalisation and life-style in neuromuscular disease: a pilot study**. *J Paediatr Child Health* 2013, **49**(3): 233-237. doi: 10.1111/jpc.12111.
- Nelson EL: **Ethical concerns associated with childhood depression.***Bioethics Forum* 2002, **18**(3-4): 55-62.
- Nikolopoulos TP, Dyar D, Gibbin KP: **Assessing candidate children for cochlear implantation with the Nottingham Children's Implant Profile (NChIP): the first 200 children**. *Int J Pediatr Otorhinolaryngol* 2004, **68**(2):127-135. doi:10.1016/j.ijporl.2003.09.019.
- Northoff G: **Brain and self--a neurophilosophical account**. *Child Adolesc Psychiatry Ment Health* 2013, **7**(1): 1-12. doi: 10.1186/1753-2000-7-28.
- Parens E, Johnston J: **Understanding the agreements and controversies surrounding childhood psychopharmacology**. *Child Adolesc Psychiatry Ment Health* 2008, **2**(1):5. doi:10.1186/1753-2000-2-5.
- Post SG: **In defense of myoblast transplantation research in preteens with Duchenne muscular dystrophy**. *Pediatr Transplant* 2010, **14**(7): 809-812. doi: 10.1111/j.1399-3046.2009.01235.x.
- Pumariega AJ, Joshi SV: **Culture and development in children and youth**. *Child Adolesc Psychiatr Clin N Am* 2010, **19**(4): 661-680. doi: 10.1016/j.chc.2010.08.002.
- Racine E et al.: **Ethics challenges of transition from paediatric to adult health care services for young adults with neurodevelopmental disabilities**. *Paediatr Child Health* 2014, **19**(2): 65-68.
- Sach TH, Barton GR: **Interpreting parental proxy reports of (health-related) quality of life for children with unilateral cochlear implants**. *Int J Pediatr Otorhinolaryngol* 2007, **71**(3):435-445. doi: 10.1016/j.ijporl.2006.11.011.
- Seki A et al.: **Incidental findings of brain magnetic resonance imaging study in a pediatric cohort in Japan and recommendation for a model management protocol.** *J Epidemiol* 2010, **20** (Suppl 2): S498-S504. doi: 10.2188/jea.JE20090196.
- Shearer MC, Bermingham SL: **The ethics of paediatric anti-depressant use: erring on the side of caution**. *J Med Ethics* 2008, **34**(10): 710-714. doi:10.1136/jme.2007.023119.
- Shiloff JD, Magwood B, Malisza KL: **MRI research proposals involving child subjects: concerns hindering research ethics boards from approving them and a checklist to help evaluate them**. *Camb Q Healthc Ethics* 2011, **20**(1): 115-129. doi: 10.1017/S096318011000068X.
- Singh I, Kelleher K: **Neuroenhancement in young people: proposal for research, policy, and clinical management**. *AJOB Neurosci* 2010, **1**(1): 3-16. doi: 10.1080/21507740903508591.
- Singh I: **Not robots: children's perspectives on authenticity, moral agency and stimulant drug treatments**. *J Med Ethics* 2013, **39**(6): 359-366. doi:10.1136/medethics-2011-100224.
- Sparks JA, Duncan BL: **The ethics and science of medicating children**. *Ethical Hum Psychol Psychiatry* 2004, **6**(1): 25-39.
- Spetie L, Arnold LE: **Ethical issues in child psychopharmacology research and practice: emphasis on preschoolers**. *Psychopharmacology (Berl)* 2007, **191**(1): 15-26. doi:10.1007/s00213-006-0685-8.
- Tan JO, Koelch M: **The ethics of psychopharmacological research in legal minors**. *Child Adolesc Psychiatry Ment Health* 2008, **2**(1): 39. doi:10.1186/1753-2000-2-39.
- Teagle HF: **Cochlear implantation for children: opening doors to opportunity**. *J Child Neurol* 2012, **27**(6):824-826. doi:10.1177/0883073812442590.
- Thomason ME: **Children in non-clinical functional magnetic resonance imaging (fMRI) studies give the scan experience a “thumbs up”**. *Bioethics* 2009, **9**(1): 25-27. doi: 10.1080/15265160802617928.
- Tusaie KR: **Is the tail wagging the dog in pediatric bipolar disorder?***Arch Psychiatri Nurs* 2010, **24**(6): 438-439. doi:10.1016/j.apnu.2010.07.011.

Books:

- Arden JB, Linford L: *Brain-Based Therapy with Children and Adolescents: Evidence-Based Treatment for Everyday Practice*. Hoboken, N.J.: John Wiley & Sons; 2008.
- Comite Consultatif National d'Ethique pour les Sciences de la Vie et de la Sante (CCNE). *Problèmes Ethiques Posés par des Démarches de Prédiction Fondées sur la Détection de Troubles Précoces du Comportement chez l'Enfant. Avis n° 95*. Paris: CCNE; 2007.
- Wasserman LH, Zambo D: *Early Childhood and Neuroscience – Links to Development and Learning*. Dordrecht: Springer; 2013.

Book Chapters:

- Chugani DC, Sukel K: **Bringing the brain of the child with autism back on track**. In *Cerebrum 2007: Emerging Ideas in Brain Science.* Edited by Cynthia A. Read. New York: Dana Press; 2007: 111-124.
- Farah MJ, Noble KG, Hurt H: **Poverty, privilege, and brain development: empirical findings and ethical implications**. In *Neuroethics: Defining the Issues in Theory, Practice, and Policy*. Edited by Judy Illes. Oxford: Oxford University Press; 2006: 277-287.
- Fegert JM: **Questions on deep brain stimulation on children and juveniles with neuropsychiatric disorders with extremely adverse course**. In *Implanted Minds: The Neuroethics of Intracerebral Stem Cell Transplantation and Deep Brain Stimulation*. Edited by Heiner Fangerau, Jörg Fegert, Thorsten Trapp. Bielefeld, Germany: Transcript Verlag; 2011: 281-289.
- Fisher RL, Fisher S: **Antidepressants for children**. In *An Anthology of Psychiatric Ethics*. Edited by Stephen A. Green, Sidney Bloch. Oxford: Oxford University Press; 2006: 239-241.
- Gomez C: **Children, maldynic pain, and the creation of suffering: toward an ethic of lamentation**. In *Maldynia: Multidisciplinary Perspectives on the Illness of Chronic Pain*. Edited by James Giordano. Boca Raton, FL: CRC Press; 2011: 221-228.
- Hadskis MR, Schmidt MH: **Pediatric neuroimaging research**. In *Oxford Handbook of Neuroethics*. Edited by Judy Illes and Barbara J. Sahakian. Oxford: Oxford University Press; 2011:389-404.
- Johnston J, Parens E: **Neuroethical issues in the diagnosis and treatment of children with mood and behavorial disturbances**. In *Handbook of Neuroethics*. Edited by Jens Clausen, Neil Levy. Dordrecht: Springer; 2014: 1673-1688.
- Larcher V: **Ethics**. In *Oxford Textbook of Palliative Care for Children*. Edited by Ann Goldman, Richard Hain, Stephen Liben. Oxford: oxford University Press; 2006: 42-62.
- Luciana M: **Development of the adolescent brain: neuroethical implications for the understanding of executive function and social cognition**. In *Oxford Handbook of Neuroethics*. Edited by Judy Illes and Barbara J. Sahakian. Oxford: Oxford University Press; 2011: 59-83.
- Moula A, Puddephatt AJ, Mohseni S: **A neuropragmatist framework for childhood education: integrating pragmatism and neuroscience to actualize Article 29 of the UN Child Convention**. In *Neuroscience, Neurophilosophy and Pragmatism: Brains at Work with the World*. Edited by Tibor Solymosi, John R. Shook. New York: Palgrave Macmillan; 2014: 215-239.
- Singh I, Kelleher K: **The case for clinical management of neuroenhancement in young people**. In *Neuroethics in Practice*. Edited by Anjan Chatterjee, Martha J. Farah. New York: Oxford University Press; 2013: 16-34.
- Stein Z, Della Chiesa BE, Hinton C, Fischer KW: **Ethical issues in educational neuroscience: raising children in a brave new world**. In *Oxford Handbook of Neuroethics*. Edited by Judy Illes and Barbara J. Sahakian. Oxford: Oxford University Press; 2011: 803-822.
- Swanson JM, Wigal T, Lakes K, Volkow ND: **Attention deficit hyperactivity disorder: defining a spectrum disorder and considering neuroethical implications**. In *Oxford Handbook of Neuroethics*. Edited by Judy Illes and Barbara J. Sahakian. Oxford: Oxford University Press; 2011:309-340.

***Dual-use neuroscientific research:***

- Canli T et al.: **Neuroethics and national security**. *Am J Bioeth* 2007, **7**(5): 3-13. doi: 10.1080/15265160701290249.
- Coupland RM: **Incapacitating chemical weapons: a year after the Moscow theatre siege**. *Lancet* 2003, **362**(9393): 1346. doi: 10.1016/S0140-6736(03)14684-3.
- Dando M: **Advances in neuroscience and the Biological and Toxin Weapons Convention**. *Biotechnol Res Int* 2011, **2011**:973851. doi: 10.4061/2011/973851.
- Dolin G: **A healer or an executioner? the proper role of a psychiatrist in a criminal justice system**. *J Law Health* 2002-2003, **17**(2): 169-216.
- Douglas T: **The dual-use problem, scientific isolationism and the division of moral labour**. *Monash Bioeth Rev* 2014, **32**(1-2): 86-105.
- Giordano J, Kulkarni A, Farwell J: **Deliver us from evil? the temptation, realities, and neuroethico-legal issues of employing assessment neurotechnologies in public safety initiatives**. *Theor Med Bioeth* 2014, **35**(1):73-89. doi:10.1007/s11017-014-9278-4.
- Latzer B: **Between madness and death: the medicate-to-execute controversy**. *Crim Justice Ethics* 2003, **22**(2): 3-14. doi:10.1080/0731129X.2003.9992146.
- Lev O, Miller FG, Emanuel EJ: **The ethics of research on enhancement interventions**. *Kennedy Inst Ethics J* 2010, **20**(2): 101-113. doi: 10.1353/ken.0.0314.
- Marchant GE, Gulley L: **National security neuroscience and the reverse dual-use dilemma**. *AJOB Neurosci* 2010, **1**(2): 20-22. doi: 10.1080/21507741003699348.
- Marks JH: **Interrogational neuroimaging in counterterrorism: a “no-brainer” or a human rights hazard?***Am J Law Med* 2007, **33**(2-3): 483-500.
- Marks JH: **A neuroskeptic’s guide to neuroethics and national security**. *AJOB Neurosci* 2010, **1**(2): 4-14. doi: 10.1080/21507741003699256.
- Moreno JD: **Dual use and the “moral taint” problem**. *Am J Bioeth* 2005, **5**(2): 52-53. doi: 10.1080/15265160590961013.
- Moreno JD: **Mind wars: brain science and the military**. *Monash Bioeth Rev* 2013*,***31**(2):83-99.
- Murphy TF: **Physicians, medical ethics, and capital punishment**. *J Clin Ethics* 2005, 16(2), 160-169.
- Nagel SK: **Critical perspective on dual-use technologies and a plea for responsibility in science**. *AJOB Neurosci* 2010, **1**(2): 27-28. doi: 10.1080/21507741003699413.
- Resnik DB: **Neuroethics, national security and secrecy**. *Am J Bioeth* 2007, 7(5): 14-15. doi:10.1080/15265160701290264.
- Roedig E**: German perspective: commentary on "recommendations for the ethical use of pharmacologic fatigue countermeasures in the U.S. military.”***Aviat Space Environ Med* 2007, **78**(5): B136-B137.
- Rose N: **The Human Brain Project: social and ethical challenges**. *Neuron* 2014, **82**(6): 1212-1215. doi: 10.1016/j.neuron.2014.06.001.
- Sehm B, Ragert P: **Why non-invasive brain stimulation should not be used in military and security services**. *Front.Hum Neurosci* 2013, **7**:553. doi: 10.3389/fnhum.2013.00553.
- Tennison MN, Moreno JD: **Neuroscience, ethics, and national security: the state of the art**. *PLoS Biol* 2012, **10**(3):e1001289. doi: 10.1371/journal.pbio.1001289.
- Voarino N: **Reconsidering the concept of ‘dual-use’ in the context of neuroscience research**. *BioéthiqueOnline* 2014, **3**/16: 1-6.
- Walsh C: **Youth justice and neuroscience: a dual-use dilemma**. *Br J Criminol* 2011, **51**(1): 21-29. doi: 10.1093/bjc/azq061.
- Wheelis M, Dando M: **Neurobiology: a case study on the imminent militarization of biology**. *Revue Internationale de la Croix-Rouge/International Review of the Red Cross* 2005, 87(859): 563-571. doi: 10.1017/S1816383100184383.
- Zimmerman E, Racine E. **Ethical issues in the translation of social neuroscience: a policy analysis of current guidelines for public dialogue in human research**. *Account Res* 2012, **19**(1): 27-46. doi: 10.1080/08989621.2012.650949.
- Zonana H: **Physicians must honor refusal of treatment to restore competency by non-dangerous inmates on death row**. *J Law Med Ethics* 2010, **38**(4): 764-773. doi:10.1111/j.1748-720X.2010.00530.x.

Books:

- Committee on Military and Intelligence Methodology for Emergent Neurophysiology and Cognitive/Neural Science Research in the Next Two Decades. *Emerging Cognitive Neuroscience and Related Technologies*. Washington, DC: National Academies Press; 2008.
- Ezzedine B, Adineh M, Satter M, Mantil JC: *Advanced Neuroscience Interface Research*. Kettering OH: Wallace-Kettering Neuroscience Institute, and Ft. Belvoir, VA: Ft. Belvoir Defense Technical Information Center; 2002.
- Giordano JJ: (Ed): *Neurotechnology in National Security and Defense: Practical Considerations, Neuroethical Concerns*. Boca Raton: CRC Press; 2014, 2015.
- Moreno JD: *Mind Wars: Brain Research and National Defense*. New York: Dana Press; 2006.
- Moreno JD: *Mind Wars: Brain Science and the Military in the Twenty-First Century*. New York: Bellevue Literary Press; 2012.

Book chapters:

- Abney K, Lin P, Mehlman M: **Military neuroenhancement and risk assessment**. In *Neurotechnology in National Security and Defense: Practical Considerations, Neuroethical Concerns*. Edited by James Giordano. Boca Raton: CRC Press, Taylor & Francis Group; 2014, 2015: 239-248.
- Balaban CD: **Neurotechnology and operational medicine**. In *Neurotechnology in National Security and Defense: Practical Considerations, Neuroethical Concerns*. Edited by James Giordano. Boca Raton: CRC Press, Taylor & Francis Group; 2014, 2015: 65-78.
- Bartolucci V, Dando M: **What does neuroethics have to say about the problem of dual use?** In *On the Dual Uses of Science and Ethics: Principles, Practices, and Prospects*. Edited by Brian Rappert, Michael J. Selgelid. Canberra, Australia: ANU Press; 2013: 29-44.
- Bell C: **Why neuroscientists should take the pledge: a collective approach to the misuse of neuroscience**. In *Neurotechnology in National Security and Defense: Practical Considerations, Neuroethical Concerns*. Edited by James Giordano. Boca Raton: CRC Press, Taylor & Francis Group; 2014, 2015: 227-238.
- Benanti P: **Between neuroskepticism and neurogullibility: the key role of neuroethics in the regulation and mitigation of neurotechnology in national security and defense**. In *Neurotechnology in National Security and Defense: Practical Considerations, Neuroethical Concerns*. Edited by James Giordano. Boca Raton: CRC Press, Taylor & Francis Group; 2014, 2015: 217-225.
- Casebeer WD: **Postscript: a neuroscience and national security normative framework for the twenty-first century**. In *Neurotechnology in National Security and Defense: Practical Considerations, Neuroethical Concerns*. Edited by James Giordano. Boca Raton: CRC Press, Taylor & Francis Group; 2014, 2015: 279-283.
- Dando M: **Neuroscience advances and future warfare**. In *Handbook of Neuroethics*. Edited by Jens Clausen, Neil Levy. Dordrecht: Springer; 2014, 2015: 1785-1800.
- Ganis G: **Investigating deception and deception detection with brain stimulation methods**. In *Detecting Deception: Current Challenges and Cognitive Approaches*. Edited by Pär Anders Granhag, Aldert Vrij, Bruno Verschuere. Hoboken, NJ: John Wiley & Sons; 2014, 2015: 253-268.
- Giordano J: **Neurotechnology, global relations, and national security: shifting contexts and neuroethical demands**. In *Neurotechnology in National Security and Defense: Practical Considerations, Neuroethical Concerns*. Edited by James Giordano. Boca Raton: CRC Press, Taylor & Francis Group; 2014, 2015: 1-10.
- Farwell JP: **Issues of law raised by developments and use of neuroscience and neurotechnology in national security and defense**. In *Neurotechnology in National Security and Defense: Practical Considerations, Neuroethical Concerns*. Edited by James Giordano. Boca Raton: CRC Press, Taylor & Francis Group; 2014, 2015: 133-165.
- Marchant GE, Gaudet LM: **Neuroscience, national security, and the reverse dual-use dilemma**. In *Neurotechnology in National Security and Defense: Practical Considerations, Neuroethical Concerns*. Edited by James J. Giordano. Boca Raton: CRC Press, Taylor & Francis Group; 2014, 2015: 167-178.
- Marks JH: **Neuroskepticism: rethinking the ethics of neuroscience and national security**. In *Neurotechnology in National Security and Defense: Practical Considerations, Neuroethical Concerns*. Edited by James Giordano. Boca Raton: CRC Press, Taylor & Francis Group; 2014, 2015: 179-198.
- McCreight R: **Brain brinksmanship: devising neuroweapons looking at battlespace, doctrine, and strategy**. In *Neurotechnology in National Security and Defense: Practical Considerations, Neuroethical Concerns*. Edited by James Giordano. Boca Raton: CRC Press, Taylor & Francis Group; 2014, 2015: 115-132.
- Murray S, Yanagi MA: **Transitioning brain research: from bench to battlefield**. In *Neurotechnology in National Security and Defense: Practical Considerations, Neuroethical Concerns*. Edited by James J. Giordano. Boca Raton: CRC Press, Taylor & Francis Group; 2014, 2015: 11-22.
- Nuffield Council on Bioethics. **Non-therapeutic applications**. In its *Novel Neurotechnologies: Intervening in the Brain*. London: Nuffield Council on Bioethics; 2013: 162-190.
- Oie KS, McDowell K: **Neurocognitive engineering for systems’ development**. In *Neurotechnology in National Security and Defense: Practical Considerations, Neuroethical Concerns*. Edited by James J. Giordano. Boca Raton: CRC Press, Taylor & Francis Group; 2014, 2015: 33-50.
- Paulus MP et al.: **Neural mechanisms as putative targets for warfighter resilience and optimal performance**. In *Neurotechnology in National Security and Defense: Practical Considerations, Neuroethical Concerns*. Edited by James J. Giordano. Boca Raton: CRC Press, Taylor & Francis Group; 2014, 2015: 51-63.
- Stanney KM et al.: **Neural systems in intelligence and training applications**. In *Neurotechnology in National Security and Defense: Practical Considerations, Neuroethical Concerns*. Edited by James J. Giordano. Boca Raton: CRC Press, Taylor & Francis Group; 2014, 2015: 23-32.
- Tabery J: **Can (and should) we regulate neurosecurity? lessons from history**. In *Neurotechnology in National Security and Defense: Practical Considerations, Neuroethical Concerns*. Edited by James Giordano. Boca Raton: CRC Press, Taylor & Francis Group; 2014, 2015: 249-258.
- Thomsen K: **Prison camp or “prison clinic?”: biopolitics, neuroethics, and national security**. In *Neurotechnology in National Security and Defense: Practical Considerations, Neuroethical Concerns*. Edited by James Giordano. Boca Raton: CRC Press, Taylor & Francis Group; 2014, 2015: 199-216.
- Tractenberg RE, FitzGerald KT, Giordano J: **Engaging neuroethical issues generated by the use of neurotechnology in national security and defense: toward process, methods, and paradigm**. In *Neurotechnology in National Security and Defense: Practical Considerations, Neuroethical Concerns*. Edited by James Giordano. Boca Raton: CRC Press, Taylor & Francis Group; 2014, 2015: 259-277.
- Wurzman R, Giordano J: **“NEURINT” and neuroweapons: neurotechnologies in national intelligence and defense**. In *Neurotechnology in National Security and Defense: Practical Considerations, Neuroethical Concerns*. Edited by James Giordano. Boca Raton: CRC Press, Taylor & Francis Group; 2014, 2015: 79-113.

## Discussion and conclusions

As demonstrated by this bibliography, the breadth and depth of the literature addressing the “ethics of neuroscience” is extensive. Indeed, many of the ethical issues fostered by brain science affect the foci, scope and conduct of clinical care, as much of neuroscientific and neurotechnological research is oriented and being explicitly directed toward translational applications in medicine. In the clinical milieu, the use of various neuroscientific approaches and tools evokes additional, and frequently more provocative, if not contentious issues, questions, problems and debates, and calls forth possible solutions that are specific to patient care, and the execution and sustainability of both (neuro)science and medicine as viable and valuable public good(s). Literature addressing such applications of brain science will be presented in Part 4 of this bibliographic series.
